# Exchange, promiscuity, and orthogonality in *de novo* designed coiled-coil peptide assemblies†

**DOI:** 10.1039/d4sc06329e

**Published:** 2024-12-09

**Authors:** Kathleen W. Kurgan, Freddie J. O. Martin, William M. Dawson, Thomas Brunnock, Andrew J. Orr-Ewing, Derek N. Woolfson

**Affiliations:** a School of Chemistry, University of Bristol Cantock's Close Bristol BS8 1TS UK d.n.woolfson@bristol.ac.uk; b Max Planck-Bristol Centre for Minimal Biology, University of Bristol Cantock's Close Bristol BS8 1TS UK; c School of Biochemistry, University of Bristol, University Walk Medical Sciences Building Bristol BS8 1TD UK; d Bristol BioDesign Institute, School of Chemistry, University of Bristol Cantock's Close Bristol BS8 1TS UK

## Abstract

*De novo* protein design is delivering new peptide and protein structures at a rapid pace. Many of these synthetic polypeptides form well-defined and hyperthermal-stable structures. Generally, however, less is known about the dynamic properties of the *de novo* designed structures. Here, we explore one aspect of dynamics in a series of *de novo* coiled-coil peptide assemblies: namely, peptide exchange within and between different oligomers from dimers through to heptamers. First, we develop a fluorescence-based reporter assay for peptide exchange that is straightforward to implement, and, thus, would be useful to others examining similar systems. We apply this assay to explore both homotypic exchange within single species, and heterotypic exchange between coiled coils of different oligomeric states. For the former, we provide a detailed study for a dimeric coiled coil, CC-Di, finding a half-life for exchange of 4.2 ± 0.3 minutes at a peptide concentration of 200 μM. Interestingly, more broadly when assessing exchange across all of the oligomeric states, we find that some of the designs are faithful and only undergo homotypic strand exchange, whereas others are promiscuous and exchange to form unexpected hetero-oligomers. Finally, we develop two design strategies to improve the orthogonality of the different oligomers: (i) using alternate positioning of salt bridge interactions; and (ii) incorporating non-canonical repeats into the designed sequences. In so doing, we reconcile the promiscuity and deliver a set of faithful homo-oligomeric *de novo* coiled-coil peptides. Our findings have implications for the application of these and other coiled coils as modules in chemical and synthetic biology.

## Abstract 2

Dynamics and exchange between coiled-coil assemblies are evaluated by a fluorescence-based assay. *De novo* designed homo-dimers to heptamers are tested for faithful and promiscuous interactions, and re-designed to deliver an orthogonal set.

## Graphical abstract

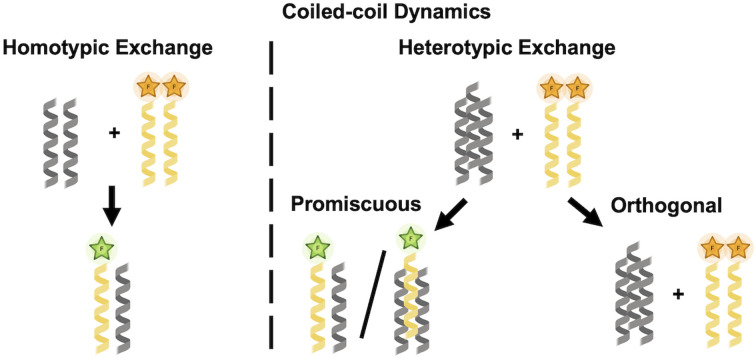

## Introduction

*De novo* protein design, defined here as designing proteins “from scratch” without starting from natural protein sequences or structures, has become a wide-spread pursuit.^1–4^ Indeed, *de novo* peptides and proteins are increasingly being used in cell biology, synthetic biology, nanotechnology, and materials science.^5–10^ Now, computational protein design is accelerating success rates for *de novo* proteins designed with atomic-level accuracy, and the application of AI methods is beginning to democratize protein design making it accessible to experts and non-experts alike.^11–13^ Nonetheless, many challenges remain, and harnessing *de novo* protein design to achieve new and effective functionalities is far from routine. A major limitation to computational methods lies in designing proteins with complex energy landscapes similar to those of native proteins.^1–4^ For instance, understanding and controlling the dynamics of *de novo* peptide assemblies and proteins would greatly aid designing functionality and expanding the current boundaries of the field.

While protein design is increasingly relying on computational and AI-based methods,^1–4,11–13^ rational peptide and protein design has also contributed considerable advances to the field.^2,4,10^*De novo* coiled coils (CCs) are particularly appealing modules for designing self-assembling systems as they are short, mutable, and sequence-to-structure relationships governing their folding and assembly are well established.^14–16^ As a result, many groups have delivered robust *de novo* CC systems, which have been characterized through to atomic structures and used in a wide variety of applications in cell and synthetic biology and in biotechnology.^10,14–16^ For example, recent in-cell studies have highlighted the potential use of *de novo* CC designs for targeting natural protein–protein interactions^17^ and effecting allosteric activation of these.^18,19^ CC dynamics and specificity are important factors to consider when applying these systems in cells, where there is an abundance of native CC structures and assemblies.^20,21^ The application of peptide-PAINT (points accumulation for imaging in nanoscale topography)^22,23^ and peptide nucleic acid (PNA)^24^ technology to facilitate fluorescence-based imaging of proteins in cells further highlights the potential of using dynamic CCs in cell biology. Orthogonal dimeric peptides allow specific labelling or transient association of fluorophores potentially with multiple proteins of interest, and optimization of these platforms has benefited from the ability to tune association of the peptides by altering peptide length and sequence.^22–24^ Yet, beyond some examples of studies of dimers^25–27^ and trimers,^28,29^ the dynamics *de novo* CCs have not been examined in detail.

Our lab has helped elucidate sequence-to-structure relationships to define oligomeric state in a fleet of *de novo* CCs from dimer to nonomer (Table 1).^30–33^ And along with others,^10,18,34–46^ we have developed rules for controlling topology: parallel *vs.* antiparallel assemblies,^17,47,48^ and hetero-peptide association.^17,47,49–51^ Whilst such sequence-to-structure relationships—or design rules—for defining oligomeric state, topology and partnering of discrete CC assemblies are now well established, the dynamics of these, and CC systems in general, are less-well explored and understood. The association of parallel heterodimer pairs has been rigorously characterized.^25–27^ However, prior to the work presented here, we have not systematically evaluated the dynamics of exchange and specificity of our *de novo* designs across the whole range of oligomeric states. Therefore, we chose to assess strand exchange between CC assemblies using fluorescence as a read out. Wendt *et al.* have described a fluorescence-based method to follow the exchange kinetics of designed α-helical leucine-zipper peptides.^52,53^ In this method, peptides are appended with an N-terminal carboxyfluorescein (FAM) moiety. In solution, the peptides dimerize and FAM self-quenches resulting in low fluorescence emission. When treated with denaturing reagents or unlabelled variants of the peptides, self-quenching is reduced and fluorescence emission increases.^52^ This method allows the observation of kinetics of CC unfolding and strand exchange.

**Table 1:** CC basis set peptide sequences

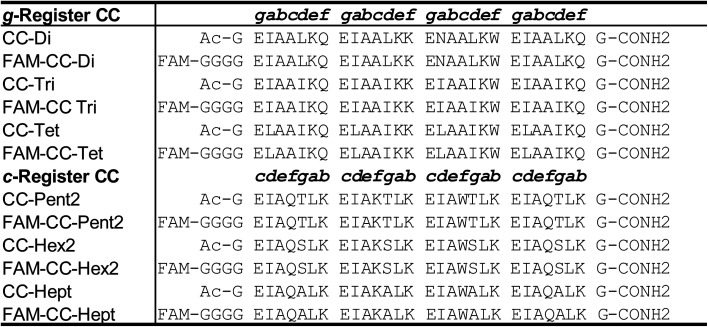

Here, we set out to understand the dynamics and specificity of homo- and heterotypic strand exchange in a broader set of *de novo* designed CC peptides. First, we adapt the aforementioned method of Wendt *et al.*^52^ to develop a fluorescence-based reporter assay for our own systems. Then, we apply this to study the kinetics and orthogonality of exchange in our published “Basis Set” of *de novo* CCs (Table 1).^30–33^ Surprisingly, we find several instances of promiscuous exchange between peptides of different oligomeric state. With this new knowledge, next we generate a set of orthogonal CCs ranging from dimer to heptamer. We use two strategies to increase selectivity in the original CC basis set: (i) strategic placement of salt-bridge interactions; and (ii) incorporation of non-canonical, hendecad repeats, in selected designed sequences. In these ways, we identify a set of CCs that show little to no heterotypic strand exchange. On this basis, we call these peptides an “Orthogonal CC Basis Set”. The intention is that these can be used in concert with each other (mixed and matched) to drive complex specific protein assemblies whilst minimizing off-target interactions for applications in chemical and synthetic biology.^10,16,18^

## Results and discussion

### A reporter system for coiled-coil strand exchange

**Fig. 1:**
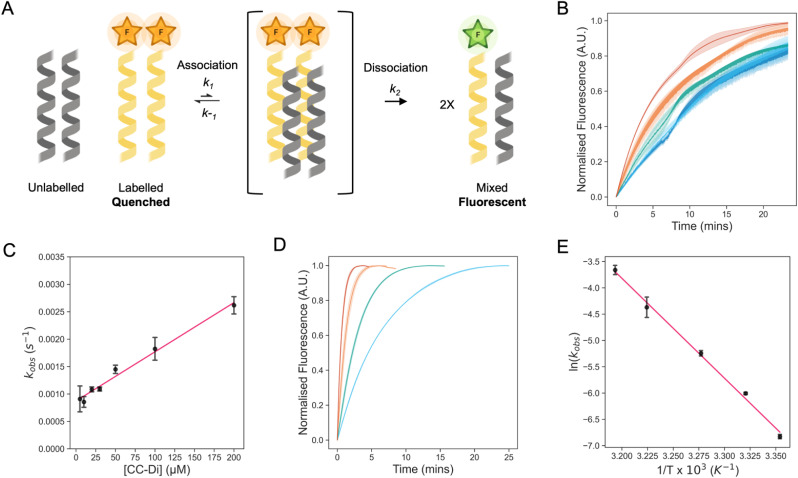
Assessing strand exchange of CC-Di using fluorescence-based measurements. (A) One possible scheme of the exchange between a quenched labelled and an unlabelled CC dimer, *via* a tetrameric steady-state intermediate, to form the fluorescent mixed species. The scheme was created with https://www.biorender.com/. (B) Normalised fluorescence time course plots for the exchange of CC-Di at different concentrations of unlabelled CC-Di. The experiments were carried out at 2 μM of labelled CC-Di. The plots are coloured by the concentration of CC-Di: blue, 20 μM; cyan, 30 μM; green, 50 μM; orange, 100 μM; and red, 200 μM. (C) Plots of the observed pseudo-first-order rate constant (*k*_obs_) for exchange at different concentrations of unlabelled CC-Di (2–200 μM). Data points are shown as the average of 3 independent replicates, error bars are for 1 standard deviation, and the line of best fit is shown in red. (D) Normalised fluorescence time course plots for the exchange of CC-Di at different temperatures (28, 32, 37, and 40 °C). The experiments were carried out at 2 μM of labelled CC-Di and 20 μM of unlabelled CC-Di. The plots are coloured by the temperature: blue, 28 °C; green, 32 °C; orange, 37 °C; and red, 40 °C. (E) Arrhenius plot for the temperature dependence of the rate constants for exchange of CC-Di. Values determined from fits to data shown in Fig. S13–S16 and Table S1.† Errors are shown to one standard deviation of independent triplicate measurements. All experiments were carried out at 25 °C in phosphate buffered saline (PBS) at pH 7.4 unless otherwise stated. Observed rate constants (*k*_obs_) were determined by fitting the normalised fluorescence time course profiles to an exponential rise (ESI eqn (2)†).

To explore the dynamics of strand exchange in and between *de novo* coiled-coil peptide assemblies, we sought a minimally invasive assay that could be performed in medium-to-high throughput. We chose fluorescence measurements, and the strategy depicted in Fig. 1A. Using a similar experimental design to Wendt *et al.*,^52^ CC peptides were synthesized in two forms: one with an N-terminal 5(6)-carboxyfluorescein (FAM) moiety (the ‘labelled’ peptide) and the other unlabelled. FAM self-quenches at concentrations above 10 mM.^54^ We reasoned that parallel homo-oligomerization of labelled peptides would bring two or more FAM moieties to within ≈1 nm, increasing the FAM effective local concentration to ≈1 M and thus quenching the fluorescence. However, if peptides can freely exchange between assembled coiled coils, the addition of an unlabelled peptide in excess should result in hetero-complexes of the labelled and unlabelled peptides and, so, reduce self-quenching leading to a fluorescence signal measured over time (Fig. 1A).^52^

To test this approach, we selected our simplest *de novo* CC, the parallel homodimer, CC-Di,^31^ synthesizing it in the two forms (Table 1). Previously, Wendt *et al.* have determined dissociation constants (*K*_D_) of similarly labelled and unlabelled CC dimers using fluorescence, chemical denaturation, and circular dichroism (CD) spectroscopy measurements. The resulting *K*_D_ values are consistent across the different methods, suggesting that association/dissociation are not perturbed significantly by labelling with FAM.^52^ Nonetheless, fluorescein has been observed to promote aggregation or enhance the stability of coiled-coil assemblies.^55^ Therefore, we tested for any influence of the fluorophore on CC association in our system by assessing different linkers between the FAM and the CC sequence.^56^ We found four Gly residues to be the simplest linker that gave consistent results in the following kinetic experiments.^56^ Next, we explored various ratios of labelled and unlabelled peptides to achieve high fluorescence values upon exchange, settling on a 1 : 10 ratio of the assembled species, as opposed to the previously used 1 : 1 ratio.^52^ For instance, for CC-Di, this was 2 μM of labelled and 20 μM of unlabelled peptide, or 1 : 10 μM of the dimeric assemblies. Under these conditions, we observed a rapid increase in fluorescence upon mixing the two peptides, Fig. 1B, indicating rapid strand exchange between the folded labelled and unlabelled species.

Subsequently, we performed kinetic experiments varying labelled to unlabelled peptide ratios to probe the mechanism of exchange for CC-Di. First, we kept the concentration of unlabelled peptide constant (200 μM) and varied the labelled peptide concentration (1–20 μM), Fig. S17.† Fitting the raw fluorescence data to single-exponential functions revealed that the observed pseudo-first order rate constant (*k*_obs_) changed little. Under these conditions, with the unlabelled peptide in excess, we expected and observed no correlation between the change in the rate constant and change in concentration of labelled peptide (see ESI† for details). Second, we kept the labelled peptide constant (2 μM) and varied the unlabelled peptide (2–200 μM), Fig. 1B and C. In this case, the rate constants increased linearly with concentration of the unlabelled peptide. This is consistent with the pseudo-first order kinetics expected with an excess of unlabelled peptide over labelled peptide. While the mechanism of exchange of FAM-CC-Di and CC-Di cannot be elucidated from these data, we propose some possible mechanisms of exchange (Fig. 1A, Schemes S3 and S4†). For instance, Fig. 1A shows one mechanism where: (1) association of labelled and unlabelled dimers form a tetrameric steady-state intermediate; which is followed by (2) dissociation of the intermediate to form fluorescent dimers composed of one copy of FAM-CC-Di and one copy of CC-Di. We posit that this is more likely to occur than the other proposed mechanisms, which are initiated by dimer dissociation, as the folded FAM-CC-Di and CC-Di dimers will be in great excess compared to dissociated monomeric variants (Tables S1 and S2†). This is because the experiments are conducted at μM peptide concentrations, but the dissociation constant of CC-Di is sub-nM.^31^ Based on conditions of pseudo-first-order reaction kinetics, we fitted the kinetic transients to single-exponential curves to obtain rate constants and calculate half-lives for the strand exchange (*t*_1/2_ = ln 2/*k*_obs_). The calculated half-life for strand exchange in 25 °C conditions where the CC-Di concentration is 200 μM was *t*_1/2_ = 4.2 ± 0.3 minutes. This is longer than for recently examined heterodimeric *de novo* CCs, with *t*_1/2_ ≈ 7–70 seconds,^27^ though these have a range of *K*_D_ values (8.1 × 10^−9^ to 3.8 × 10^−5^ M) that is weaker than CC-Di and consistent with the faster exchange. Finally, as expected, the exchange rate constant increased with temperature, Fig. 1D, and an Arrhenius analysis returned an activation enthalpy of 37.9 ± 1.9 kcal (mol of dimer)^−1^, Fig. 1E. This is comparable to the activation energy determined for the unfolding of the GCN4-p1 leucine-zipper dimer, 30.8 kcal (mol of dimer)^−1^.^57^

We conducted similar kinetic strand-exchange experiments for the other *de novo* designed CC basis set peptides, *i.e.*, CC-Tri through CC-Hept.^16,30–33^ Although these all showed increases in fluorescence consistent with strand exchange to produce mixed labelled/unlabelled species, the kinetic mechanisms for exchange in these higher-order oligomers are more complicated than those shown in Fig. 1, Schemes S3 and S4† for CC-Di.^56^ As such, these data could not be fitted using simple rate equations. We propose that this is because, rather than one dominant transient species and a single mixed fluorescent equilibrium species, many species with different numbers of labelled (l) and unlabelled (u) peptides are possible, and the number of combinations increases with increasing oligomer state. For instance, for CC-Tri there are 4 assembled parallel species alone: u-u-u, l-l-l, u-u-l, and u-l-l. This led us to conclude that, for the larger oligomer states, rather than following and quantifying the kinetic traces directly, we needed to focus on the endpoints in the exchange experiments.

### Homotypic and heterotypic strand exchange for the CC basis set

**Fig. 2:**
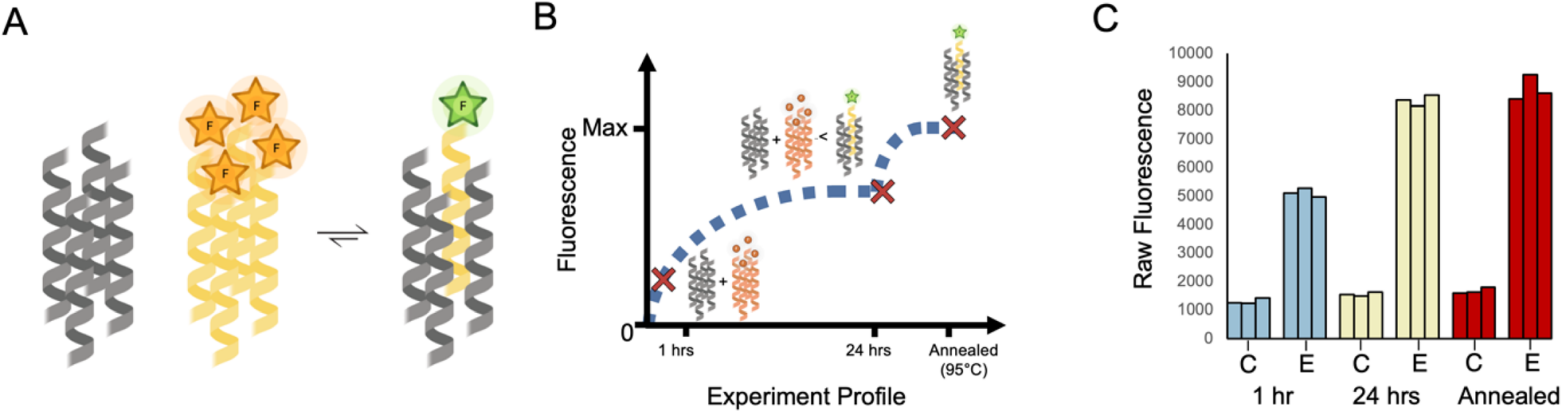
Homotypic exchange of CC-Tet. (A) Cartoon for the assumed equilibrium reached when labelled and unlabelled variants of CC-Tet are mixed in a 1 : 10 ratio. For simplicity, excesses of the unlabelled peptide and any intermediate species are omitted. This cartoon was created with https://www.biorender.com/. (B) Cartoon for the anticipated changes in population of the quenched and free FAM-CC-Tet over time where the major species are represented at different time points. (C) Raw data from experiments following the protocol outlined in the text and depicted in panel B. (See ESI† for experimental details.) The data were collected for two different samples: (1) a control sample, represented as “C,” which corresponds to 2 μM FAM-CC-Tet in PBS and (2) an exchange sample, represented as “E,” which corresponds to 2 μM FAM-CC-Tet + 20 μM CC-Tet in PBS. The fluorescence measurements were collected at three different time points (1 h, 24 h, and after annealing) and repeated 3 times. All fluorescence data of FAM-CC-Tet mixtures were subsequently normalised against the data collected for the annealed samples where the averaged observed fluorescence of FAM-CC-Tet in buffer was set to zero and the averaged observed fluorescence of 1 : 10 ratio of FAM-CC-Tet to CC-Tet was set to one.

With the above in mind, we adopted the approach of monitoring the extent of fluorescence (again, as a proxy for exchange) at three points as shown in Fig. 2A. This involved mixing the labelled and unlabelled peptides (with the assumed assemblies at 1 μM and 10 μM, respectively) under folded conditions incubated at 25 °C. Aliquots were taken for fluorescence measurements at 1 h and 24 h time points. Then, the remaining samples were heated to 95 °C for 5 minutes and cooled to 25 °C over 2 hours. Regardless of differences in kinetics, this final annealing step aimed to facilitate any further possible strand exchange prior to recording the final fluorescence signal.

Taking the CC-Tet homomeric exchange as an example, we anticipated exchange between labelled and unlabelled variants of CC-Tet and, with an excess of unlabelled peptide, the equilibrium should shift toward a population of unquenched FAM (Fig. 2A). Depending on the rate of exchange, it would take time for the mixture to reach this equilibrium. Earlier time points would still have a high population of unmixed FAM-labelled peptide, corresponding to lower fluorescence values. Indeed equilibrium, and consequently high fluorescence values, may only be reached after annealing (Fig. 2B). This anticipated behaviour was apparent in the fluorescence data (Fig. 2C). For the FAM-labelled peptide alone in buffer, the fluorescence changed little over time (Fig. 2C). However, the mixture of labelled and unlabelled CC-Tet gave comparatively high fluorescence values at the 1 h and 24 h time points, with the latter comparable to the signal after annealing. These data illustrate the utility of the endpoint method for assessing strand exchange in CC systems.

**Fig. 3:**
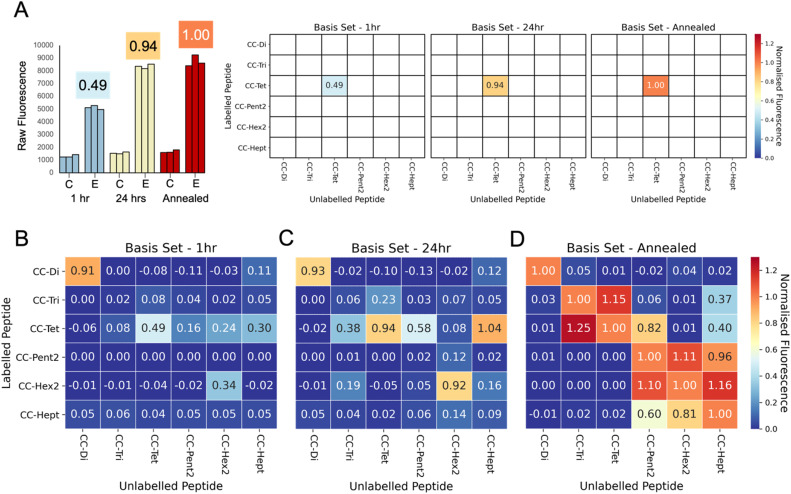
Summary of exchange across the whole CC basis set. Heat maps for the normalised fluorescence values for all pairs of peptide mixtures. (A) The raw fluorescence data observed in the homotypic exchange of CC-Tet are shown with the values calculated after normalisation in the bar graph on the left. For these data, the annealed control (2 μM FAM-CC-Tet in PBS) intensity was set to zero, and the annealed exchange (2 μM FAM-CC-Tet + 20 μM CC-Tet in PBS) intensity was set to one. These normalised values are the shown in the dummy heatmaps on the right where the labelled CC-Tet position on the *y*-axis intersects with unlabelled CC-Tet position on the *x*-axis. (B–D) complete heat maps showing measurements taken at 1 h (B) and 24 h (C) after mixing, and after a subsequent annealing step (D). With a few exceptions, the post-annealed values are much higher than those observed after 1 h and 24 h. The post-annealed data show exchange within the homotypic mixtures (diagonal values), between CC-Tri and CC-Tet, and between type II coiled coils (CC-Pent 2, CC-Hex 2, and CC-Hept).

Note: in comparison to homotypic exchange, it is less clear what the composition of the heterotypic peptide mixtures, the oligomeric state and the stoichiometry of potential mixed assemblies, will be at equilibrium. The mechanism of exchange is also less obvious. For these reasons, we cannot expect to see the same trends (increasing fluorescence over time followed by maximum fluorescence after annealing), that we observe in the homotypic exchange experiments. As annealing should allow the systems to reach a thermodynamic minimum, we argue that these values best represent the possible full level of exchange.

With this method in hand, we assessed all combinations of labelled and unlabelled peptides for the whole CC basis set (Table 1). We applied min–max scaling to the raw fluorescence data using the averaged fluorescence value of the pure annealed labelled peptide as the minimum, and that for the annealed 1 : 10 mixture of the homotypic exchange corresponding to the labelled peptide as the maximum, Fig. 3. To illustrate this procedure, the raw fluorescence values from the homotypic exchange of CC-Tet from Fig. 2C are shown next to the normalised values at the corresponding positions of dummy heat maps in Fig. 3A. Completed heat maps are used throughout the rest of this paper to summarise the full datasets. In these, high fluorescence values, *i.e.* high amounts of exchange, are shown in red, and low fluorescence/exchange in blue.

Focusing on the diagonal in Fig. 3B, after a 1 hour incubation at 25 °C, CC-Di had undergone significant homotypic exchange and CC-Tet and CC-Hex2 had partly exchanged with their labelled variants, whereas, CC-Tri, CC-Pent2, and CC-Hept had exchanged little. The CC-Di data are consistent with the known thermal stability of this assembly relative to the others: CC-Di has the lowest *T*_M_ of the CC basis set (78 °C at 50 μM peptide), whereas the others are hyper-thermostable and not completely unfold upon heating to 90 °C.^30–33^ Moreover, from the off-diagonal cells, there were signs of heterotypic exchange; for instance, between labelled CC-Tet and the unlabelled higher-order CC-Pent2, CC-Hex2, and CC-Hept. After 24 hours at 25 °C (Fig. 3C), these trends were accentuated, although for some peptides (*e.g.* CC-Tri, CC-Pent2, and CC-Hept) little homotypic exchange had occurred. As expected, the heating-and-cooling step increased exchange across the whole set of combinations, and further highlighted the potential for ‘promiscuous’ heterotypic exchange (Fig. 3D). For example, CC-Tri and CC-Tet exchanged with each other, as did the higher-order CCs, CC-Pent2, CC-Hex2, and CC-Hept. These two classes—*i.e.*, CC-Tri plus CC-Tet, and CC-Pent2, CC-Hex2 plus CC-Hept—are structurally distinct: the trimer and tetramer have consolidated hydrophobic cores, whilst the others have central channels and are α-helical barrels.

The key results and our interpretations from these experiments on mixing the original CC basis set peptides are as follows. (1) CC-Di only undergoes homotypic exchange; *i.e.*, it is a faithful design that is orthogonal to the other *de novo* CCs (see topmost row and leftmost column of Fig. 3D). This is likely because it is the only design with a buried polar residue—an Asn at the central *a* site, Table 1—incorporated to specify the parallel dimer.^31^ Thus, exchange with any other CC, which have exclusively hydrophobic cores, would be energetically unfavourable. (2) CC-Tri and CC-Tet exchange with each other, but less so with the higher-order CCs, (although labelled CC-Tri and CC-Tet do exchange with unlabelled CC-Pent2 and CC-Hept, and with CC-Hept, respectively, to some extent). We propose that this is because CC-Tri and CC-Tet have similar heptad repeats, E*a*AAIKX in *g* →*f* register, with only the residues at *a* being different (*a* = Ile in CC Tri, and Leu in CC Tet).^31^ This opens possible CC-Tri : CC-Tet heterotypic interactions that we had not considered before conducting these exchange experiments. Finally, (3) the higher-order CCs, CC-Pent2, CC-Hex2, and CC-Hept, also interact with each other. However, this is to differing degrees and is only significant after heating and cooling (compare the bottom-right quadrants of Fig. 3B–D). Again, the peptide sequences are key to understanding this. These peptides are type-II CCs in which residues at *g*, *a*, *d*, and *e* sites engage in helix–helix interactions.^16,30,32,33,58^ Moreover, the *g* → *f* heptad repeats are similar, *i.e. g*LKEIAX with *g* = Thr for CC-Pent2, Ser for CC-Hex2, and Ala for CC-Hept. Whilst these subtle changes demonstrably give oligomer-state specificity to the homomers,^30,32,33^ there is unforeseen potential for heterotypic interactions.

### Establishing orthogonal trimeric and tetrameric coiled coils

**Fig. 4:**
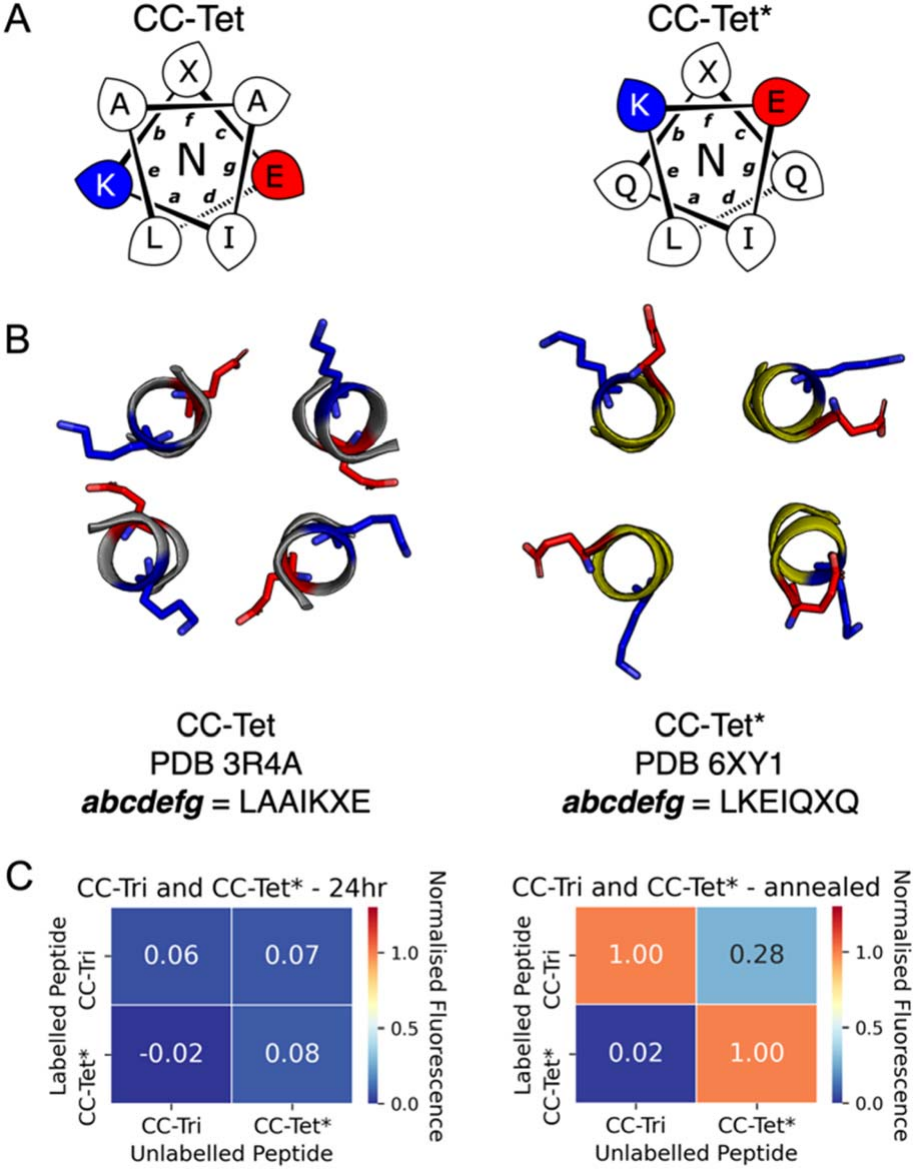
Improved orthogonality between CC-Tri and CC-Tet*. (A) Helical-wheel representations of the heptad-repeat sequences of CC-Tet and CC-Tet*. In CC-Tet, residues that promote interhelical salt-bridge interactions (lysine and glutamic acid) are positioned at the *e* and *g* positions, whereas in CC-Tet* these are at *b* and *c*. (B) Slices through the X-ray crystal structures of CC-Tet and CC-Tet* show the structural consequences of the different placements of lysine and glutamic acid residues. Except for these, side chains are omitted for clarity. (C) Normalised fluorescence date for exchange between CC-Tri and CC-Tet*. After annealing, values for the hetero-mixtures (off diagonal) are lower than those for the homomers, indicating orthogonality over the CC-Tri/CC-Tet combination (Fig. 3C).

Given these new insights into the CC basis set from the fluorescence-based exchange experiments and the need for orthogonal components for applications in chemical and synthetic biology, we sought to improve the orthogonality of the more-promiscuous CCs using rational redesign. Our aim was to make minimal mutations that would not compromise the CC oligomer state or stability. We started with the CC-Tri and CC-Tet sequences, reasoning that salt-bridge interactions could be exploited to achieve orthogonality between these trimeric and tetrameric CCs. This was prompted by a variant of the latter, CC-Tet*,^59^ where interhelical salt-bridging lysine and glutamate residues are moved to the *b* and *c* positions, compared to the *e* and *g* positions in the original CC-Tri and CC-Tet sequences (Fig. 4A and B). CC-Tet* is more robust as a parallel tetramer than the original CC-Tet design; minor variations to the latter can result in trimers.^31,59^ Therefore, we tested the exchange between CC-Tri and CC-Tet* (Fig. 4C). We observed lower fluorescence values for the mixed heterotypic exchange in comparison to high fluorescence values of the homotypic exchange and the CC-Tri + CC-Tet experiments (compare Fig. 3 and 4C). These data show that orthogonality can be driven by strategic positioning of salt-bridge interactions.

### Designing orthogonal higher-order coiled coils using alternative sequence repeats

For the α-helical barrels, CC-Pent2, CC-Hex2, and CC-Hept, designing orthogonal variants was more challenging. Shifting the salt-bridge interactions was not an option as these were already at *b* and *c* in all sequences (Table 1). Additionally, apart from pentamers, where glutamine can be introduced into the *a*/*d* sites,^56,60–62^ there are few examples to guide the rational design of polar layers within these higher-order CCs. Therefore, we took a different approach of incorporating non-heptad sequence repeats into these peptide sequences. In canonical heptad repeats, predominantly hydrophobic side chains are spaced 3 and 4 residues apart at the *a* and *d* sites of the *a* → *g* heptad repeats (Fig. 4A and S38†). This generates amphipathic helices, and the *a*/*d* hydrophobic seams of these drive helix associated and CC formation. Variations of this 3-4 spacing can lead to alternative CC sequences and structures. For instance, in 11-amino acid, hendecad repeats core-forming residues are spaced 3-4-4 apart at the *a*, *d*, and *h* sites of *a* → *k* repeats (Fig. S38†).^16,63–67^ Consequently, while heptad repeats drive association of left-handed CCs, hendecads lead to right-handed quaternary structures (Fig. S38†).^16,63–67^ We reasoned that replacing heptad with hendecad repeats at different locations in the pentamer, hexamer, and heptamer sequences should alter the hydrophobic seams and, in turn, might confer homo-specificity in the resulting sequences.

**Fig. 5:**
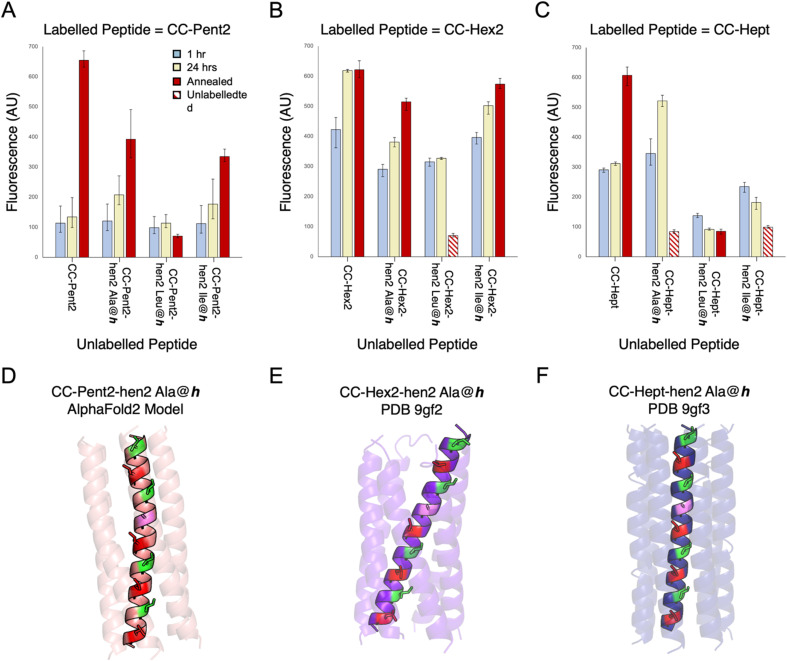
Mixing heptad and hendecad repeats to improve orthogonality. (A–C) Fluorescence-based orthogonality screens of α-helical-barrel variants incorporating hendecad repeats, CC-Pent2-hen2, CC-Hex2-hen2, and CC-Hept-hen2, where the *h* positions were Ala, Leu, or Ile. This initial screen was performed against FAM-labelled variants of the parent, heptad-based peptides. Compared with homotypic controls (*i.e.* FAM-CC-Pent2 + CC-Pent2, FAM-CC-Hept + CC-Hept), the CC-Pent2-hen2 and CC-Hept-hen2 variants showed marked decreases in fluorescence indicating less exchange and thus improved orthogonality (D and F). However, the CC-Hex2 variants still showed some cross-exchange and promiscuity (E). Note: some of the hen2 variants were not stable up to 95 °C and precipitated from solution after annealing as indicated by the striped data columns. (D–F) An AlphaFold2 (ref. 68) model and X-ray crystal structures of CC-Pent2-hen2, CC-Hex2-hen2, and CC-Hept-hen2 (all with Ala at *h*) respectively are shown with the *a* positions coloured red, *d* in green, and *h* in lilac.

Initially, we made several variants of CC-Pent2, CC-Hex2, and CC-Hept that extended the second heptads into hendecad repeats (Table 2), with the new designs denoted by the suffix “-hen2”. In these, the original *a-g* sequences were maintained, and the new *i-k* sites were all made Ala. What to place at the core-forming *h* sites was less clear. So, we tested Ala, Ile, and Leu in each of the three designs. These were tested in the endpoint fluorescence assay against their FAM-labelled parent, heptad-based peptides (Fig. 5A–C). We reasoned that any orthogonality in these experiments would indicate potential orthogonality with the other oligomeric states. Generally, for the CC-Pent2 and CC-Hept designs, these experiments revealed less exchange between the heptad and hendecad variants compared to the homotypic exchange of the parents, indicating that the strategy had increased orthogonality. However, the experiments with CC-Hex2 showed considerable exchange and, therefore, promiscuity between all pairings.

**Table 2:** Hendecad incorporated variants of the α-helical barrels, CC-Pent2, CCHex2, and CC-Hept^a^

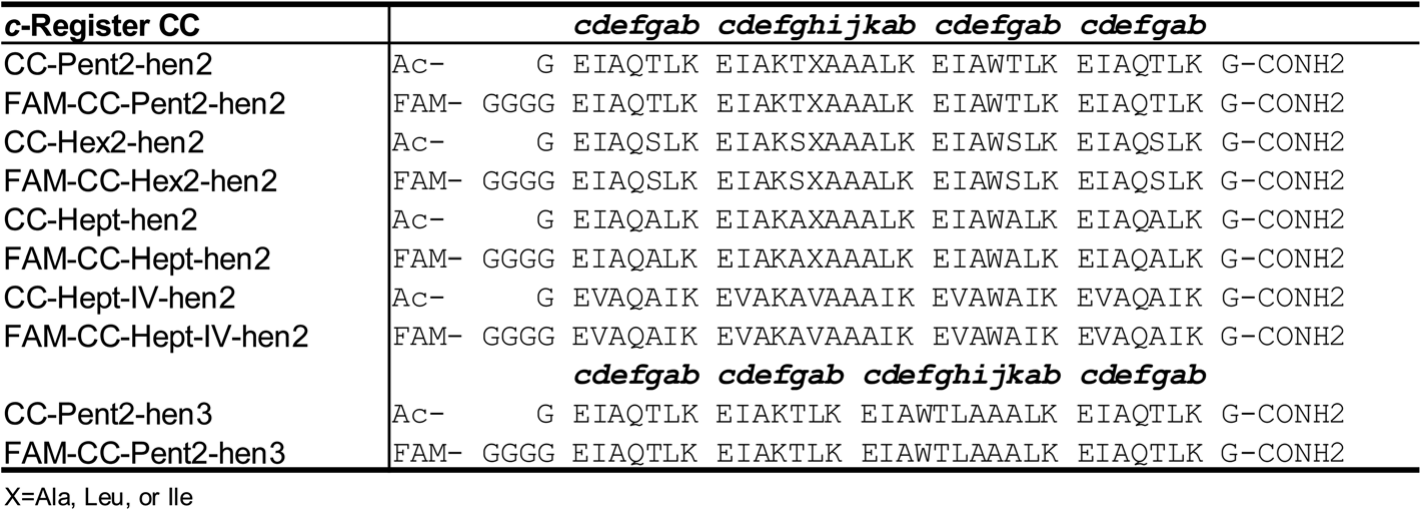

a X=Ala, Leu, or Ile.

To investigate this and test our hypothesis of altering the hydrophobic seams, we compared model and experimental structures of CC-Pent2-hen2, CC-Hex2-hen2, and CC-Hept-hen2 with Ala at the *h* position. An AlphaFold2 (ref. 68) model of CC-Pent-hen2 indicated a marked twist in the monomeric helix (Fig. 5D), though an X-ray crystal structure obtained for CC-Hept-hen2 showed a more subtle change (Fig. 5F). These features are consistent with combining left- and right-handed CC repeats, and the observed orthogonality to the parent peptides. However, an X-ray crystal structure of CC-Hex2-hen2 with Ala at *h* revealed a similar supercoiling to the parent despite the incorporation of the hendecad repeat (Fig. 5E), possibly explaining the continued promiscuity observed between the CC-Hex2 variants.

### An orthogonal coiled-coil basis set

**Fig. 6:**
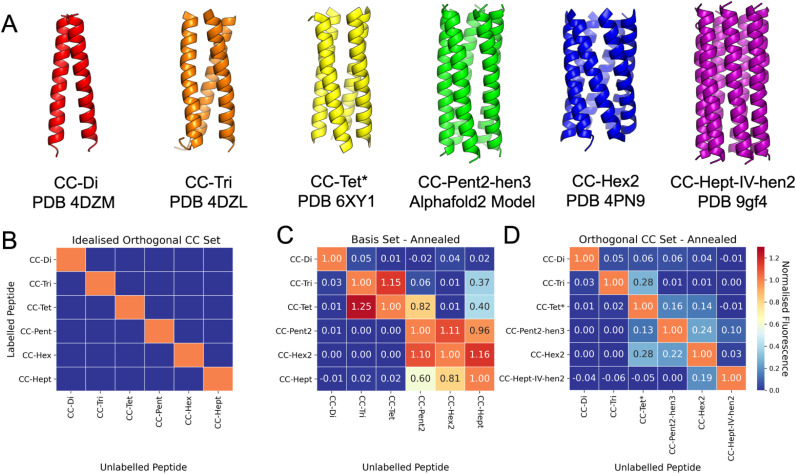
An Orthogonal CC basis set. (A) X-ray crystal structures and an AlphaFold2 (ref. 68) model for the assembled peptides in this set. The pentamer and heptamer were designed in this study, the other peptides have been published elsewhere.^30–33,59^ The heat map shown in panel B represents the ideal case where all peptides are orthogonal to one another, *i.e.* only homotypic exchange is observed. Heat maps from the post-annealing fluorescence exchange data for the CC basis set (C) and the Orthogonal CC basis set (D) are shown side-by-side for comparison. The values in panel D indicate considerable orthogonality across the new set: the homotypic exchange (diagonal) values are all higher than those for any of the heterotypic exchange experiments (off-diagonal). All mixtures containing CC-Hept-IV-hen2 and/or FAM-CCHept-IV-hen2 were annealed to 75 °C instead of 95 °C as these peptides were not stable (with respect to precipitation) at the higher temperature (see Fig. S47†).

Our final goal was to achieve as much orthogonality across a revised CC basis set as possible. Based on the above experiments collectively, we reasoned that the original CC-Di, CC-Tri, and CC-Hex2 sequences could be kept, and that CC-Tet* could be substituted for CC-Tet. For CC-Pent2 and CC-Hept, initially, we replaced the third and second heptads, respectively, with hendecad repeats to give CC-Pent2-hen3 and CC-Hept-hen2, and using the Leu-at-*h* variants for both (Table 2). Unfortunately, FAM-CC-Hept-hen2 was not soluble in PBS buffer, so we switched to a heptamer sequence with Ile in the *a* sites and Val at *d* and *h* (CC-Hept-IV-hen2, Table 2). This proved more soluble and retained orthogonality. CC-Pent-hen3 was confirmed as a pentamer by analytical ultracentrifugation (AUC) experiments (Table S9 and Fig. S48†). CC-Hept-IV-hen2 sedimented as a hexamer in AUC (Table S9 and Fig. S49†), but an X-ray crystal structure revealed a heptamer (Fig. 6A and Table S8†). Finally, we tested all the proposed revised basis set, denoted Orthogonal CC basis set, in the endpoint fluorescence assay (Fig. 6D and S51†). The normalised fluorescence data from the mixed peptides showed association for some pairings at the 1 h and 24 h time points (Fig. S51†); specifically, some promiscuity remained between the tetra-, penta- and hexametric CCs after annealing, Fig. 6D. However, overall, this revised set of peptides showed significant improvements in orthogonality across the whole set compared with the original basis set going into this study (Fig. 6C). As described above, CC-Tet* gave little strand exchange with CC-Tri and showed a marked decrease in exchange with CC-Pent2-hen3 compared to exchange between CC-Tet and CC-Pent2. We had also observed promiscuity between all of the α-helical barrels in the CC basis set. However, for the revised Orthogonal CC basis set, this exchange was vastly reduced.

## Conclusions

Here, we have developed a method for assessing association between coiled-coil (CC) peptides using a fluorescence-reporter strategy. This is applied to assess homo- and heterotypic strand exchange between a published set of *de novo* CCs ranging in oligomeric state from dimer to heptamer.^30–33^ We observe that while the dimer, CC-Di, is completely orthogonal, CC designs with related helix–helix interfaces but different oligomeric states can exchange promiscuously. For example, the trimer, CC-Tri, and the tetramer, CC-Tet, which have Type I interfaces (meaning that the *g*, *a* and *d* residues of the *a* → *g* heptad sequence repeats contribute to the helix–helix interactions), exchange with another. Similarly, peptides with Type II interfaces where the *g*, *a*, *d*, and *e* residues all engage in helix–helix interactions—namely the pentameric, hexameric, and heptameric α-helical barrels, CC-Pent2, CC-Hex2 and CC-Hept—also exchange with each other. For certain applications of the original CC basis set where the designed peptides are used on their own, or paired with orthogonal designs (*e.g.*, CC-Di with CC-Tri), this will not be an issue. However, we wanted to achieve a basis set that is as orthogonal as possible and, from which, peptides can be used in many combinations. To address this, using the insight from the data and analysis presented here, we have tested two different strategies for increasing specificity of coiled coils: (i) alternate placement of salt bridge-promoting residues, lysine and glutamate to discriminate the tetramer from the trimer; and (ii) incorporation of hendecad repeats to separate the α-helical barrels. The first strategy has been successful as demonstrated by the low heterotypic exchange between CC-Tri (with lysine and glutamate at the *e* and *g*, respectively) and CC-Tet* (where lysine and glutamate are moved to *b* and *c*, respectively).^59^ The second strategy differentiates the α-helical barrels, CC-Pent2, CC-Hex2, and CC-Hept, by introducing hendecad repeats in a pentamer and heptamer to give CC-Pent2-hen3 and CC-Hept-IV-hen2. A search for hendecad repeats in structurally validated CC assemblies of the CC + database gave only one example of a pentameric assembly and no examples of heptameric assemblies.^69^ However, sequence analysis and model predictions suggest that higher oligomeric CC assemblies with hendecad repeats do occur in nature.^67^ Fluorescence measurements show minimal exchange between a revised set of CC peptides: CC-Di, CC-Tri, CC-Tet*, CC-Pent2-hen3, CC-Hex2, and CC-Hept-IV-hen2. We have denoted this group of peptides as the Orthogonal Coiled-coil basis set. Orthogonal CC dimers have been used to design macromolecular assemblies,^70–74^ protein–protein interactions in cells,^17–19^ and scaffolds for specific fluorophore labelling of proteins of interest.^22–24^ In a similar vein, we envision this wider set as building blocks will be useful in protein design and applications in chemical and synthetic biology.

## Data availability

Details of the experimental methods and additional experimental data are provided in the ESI.† Crystallographic data for CC-Hex-hen2 Ala@h, CC-Hept-hen2 Ala@h, and CC-Hept-IV-hen2 have been deposited to the PDB and given the accession codes 9gf2, 9gf3, and 9gf4 respectively. Fluorescence data was analysed using a code which is available on the Woolfson lab github (https://github.com/woolfson-group/CC_exchange).

## Author contributions

KWK, FJOM, WMD, and DNW conceptualized the study, developed the methodology, and designed the peptides and experiments. KWK, FJOM, and TB contributed to investigation (synthesized the peptides, conducted the experiments, determined the peptide X-ray crystal structures). KWK and FJOM performed formal analysis of the data with advice from AJO. KWK, FJOM, and DNW wrote the paper. All authors have read and contributed to the preparation of the manuscript.

## Conflicts of interest

There are no conflicts to declare.

## Supplementary Material

SC-016-D4SC06329E-s001
