# Supplementary material for: Exchange, promiscuity, and orthogonality in de novo designed coiled-coil peptide assemblies
Source: Chem Sci. 2024 Dec 9;16(4):1826–36. doi: 10.1039/d4sc06329e (PMC11664599; doi:10.1039/d4sc06329e)
Supplement: SC-016-D4SC06329E-s001 [file SC-016-D4SC06329E-s001.pdf]

Supplementary Information (SI) for Chemical Science.
This journal is © The Royal Society of Chemistry 2024

Supporting Information for: Exchange, promiscuity, and orthogonality in *de novo* designed coiled-coil peptide assemblies

Kathleen W. Kurgan,[1,†] Freddie J. O. Martin,[1,†] William M. Dawson,[1] Tom Brunnock,[1] Andrew J. Orr-Ewing,[1] and Derek N. Woolfson[1,2,3,4*]

[1]School of Chemistry, University of Bristol, Cantock's Close, Bristol BS8 1TS, UK

[2]School of Biochemistry, University of Bristol, Medical Sciences Building, University Walk, Bristol BS8 1TD, UK

[3]Bristol BioDesign Institute, School of Chemistry, University of Bristol, Cantock's Close, Bristol BS8 1TS, UK

[4]Max Planck-Bristol Centre for Minimal Biology, University of Bristol, Cantock's Close, Bristol BS8 1TS, UK

[†]Contributed equally to this work.
*To whom correspondence should be addressed: D.N.Woolfson@bristol.ac.uk

Methods……S2-S6
- **General**……S2
- **Peptide Synthesis and Purification**……S2-S3
- **Biophysical Characterization**……S3-S4
- **Fluorescence Measurements**……S4-S6
- **X-Ray Structure Determination**……S6

CC-Di Kinetics Data……S7-S22
CC Basis Set Data……S23-S35
- **Sequences**……S23
- **MALDI and AHPLC**……S23-S27
- **CD Characterization**……S27-S29
- **Fluorescence Measurements**……S30-S35

CC-Tri and CCTet* Fluorescence Measurements……S36
Orthogonal CC Set Data……S36-S49
- **Sequences**……S36
- **MALDI and AHPLC**……S36-S38
- **CD Characterization**……S38-S39
- **X-Ray Structures**……S40-S41
- **Analytical Ultracentrifugation Data**……S42
- **Fluorescence Measurements**……S43-S49

References……S50-S51

Methods

General

All solvents, chemicals, and reagents were purchased from commercial sources and used without further purification. Fluorenylmethoxycarbonyl(Fmoc)-α-L-amino acids, Rink amide MBHA resin for solid-phase peptide synthesis (SPPS) and N,N-dimethylformamide (DMF) were purchased from Sigma-Aldrich and Cambridge Reagents. Coupling reagents Oxyma Pure and diisopropylcarbodiimide (DIC) were purchased from Fluorochem. Morpholine, and trifluoroacetic acid (TFA) were purchased from Sigma-Aldrich; pyridine was from Thermo Fisher; triisopropylsilane (TIPS) was from Acros Organics. All other chemicals were reagent grade and purchased from Sigma-Aldrich. Peptide biophysical characterization data were recorded in phosphate buffered saline (PBS; 8.2 mM sodium phosphate dibasic, 1.8 mM potassium phosphate monobasic, 137 mM NaCl, 2.4 mM KCl), pH 7.4. Peptide characterisation data for CC-Di, CC-Tri, CC-Tet, CC-Tet*, CC-Pent2, CC-Hex2, and CC-Hept have been published previously.[1-3]

Peptide Synthesis

Peptides were prepared by standard Fmoc microwave-assisted solid-phase peptide synthesis using a Liberty Blue™ automatic synthesizer (CEM) with inline UV monitoring. Pre-swelled Rink amide MBHA resin (100 µmol) was added to the sample loader. Fmoc-protected amino acids were coupled to the resin via addition of 2.5 mL of 0.2 M Fmoc-amino acid (5 equiv.) in DMF, 1.0 mL of 1 M *N,N'*-diisopropylcarbodiimide in DMF (10 equiv.) and 1.0 mL of 0.5 M ethyl cyano(hydroxyamino)acetate (5 equiv.) in DMF, as recommended by CEM, to the reaction vessel. Deprotection of the Fmoc group was performed by addition of 20% (v/v) morpholine in DMF into the reaction vessel and heating the reaction vessel to 90 ºC for 1 min (125 W 30 s, 32 W 60 s).

Following the deprotection of the N-terminal Gly residue, the resin was transferred to a fritted syringe. Unlabelled peptides were acetyl capped by addition of 0.5 mL acetic anhydride and 0.25 mL of pyridine in excess DMF. The resulting mixture was rocked at RT for 20 min. Carboxy-fluorescein (5 equiv.) was coupled onto the N-terminus to generate labelled peptides using the coupling conditions described above and rocked at RT for 2 hrs. Acetyl and FAM-capped peptides were washed 4X with DMF and 4X with DCM prior to cleavage. Peptides were then cleaved from the resin by addition of 8 mL of a cleavage cocktail consisting of 2.5 % H_2O, 2.5% triisopropylsilane and 95% trifluoroacetic acid (TFA). The solution was rocked for 2 hours and subsequently filtered through the fritted syringe. The resin was washed two more times with TFA, and the filtered TFA solutions were combined. The TFA in the combined solution was evaporated under a stream of nitrogen. To the resulting brown oil was added chilled diethyl ether, which caused precipitation. Crude peptide was isolated using centrifugation, re-dissolved in 1:1 MeCN:H_2O and lyophilized to yield a white powder.

Peptide Purification

Crude products of peptide syntheses were purified via RP-HPLC using a Jasco system consisting of a UV-4075 UV/Vis detector, PU-4180 HPLC Pump, LC-Net II/ADC computer-instrument interface, and co-2060 Plus HPLC column thermostat. The purification was conducted with Luna® C18 (Phenomenex) column (150 mm x 10 mm, 5 µm particle size, 100 Å pore size) using a gradient elution of 20-80%, 30-90%, or 40-100% B solvent over 30 minutes. Solvent A is 0.1% TFA in MilliQ H_2O, and solvent B is 0.1% TFA in HPLC-grade acetonitrile. When required, the column was heated to 50 ºC to assist peptide elution. Chromatograms were monitored at 220 and 280 nm wavelengths. The presence of the desired peptide was established via matrix-assisted laser desorption ionization-time of flight (MALDI-TOF) mass spectroscopy attained via use of a Bruker ULTRAFLEX™ instrument in positive-ion reflector mode. Peptides were spotted on a ground steel target plate using α-cyano-4-hydroxycinnamic acid dissolved in 1:1 MeCN/H_2O as the matrix. Expected monoisotopic masses of the singly protonated species of each peptide are listed in Tables S2 and S5. Purity was quantified utilizing a Jasco 2000 HPLC and a Phenomenex Kinetex C18 (100 x 4.6 mm, 5 µm particle size, 100 Å pore size) column. Chromatograms were monitored at 220 and 280 nm wavelengths. The linear gradient was 20 – 100% MeCN in water (each containing 0.1% TFA) over 25 min at a flow rate of 1 mL/min.

Determining Concentration of FAM-labelled Peptides

The concentration of purified lyophilized peptide dissolved in MilliQ water was determined by measuring the absorbance of the sample at 280 nm using a ThermoScientific 2000 UV-Vis spectrometer. For all non-labelled peptides, the ε_{280nm} = 5500 $cm^{-1}M^{-1}$ as each contains a single tryptophan. The protocol for determining the concentration of the FAM-labelled peptides follows the one described in "Exploring the Dynamic and Conformational Landscape of α-Helical Peptide Assemblies."[4] Peptide samples were diluted in 25 mM Tris pH 8, 1% SDS buffer and their absorbance measured at 495 nm. The extension coefficient used for FAM-labelled peptides in 25 mM Tris pH 8, 100 mM NaCl, 1% SDS buffer was previously measured as ε_{495nm} = 89000 $cm^{-1}M^{-1}$.

Circular Dichroism

Circular dichroism (CD) data were collected on a JASCO J-810 spectropolarimeter fitted with a Peltier temperature controller. Peptide samples were made up as 50 or 25 µM peptide solutions in phosphate buffered saline (PBS; 8.2 mM sodium phosphate dibasic, 1.8 mM potassium phosphate monobasic, 137 mM NaCl, 2.4 mM KCl), pH 7.4. Data were collected in a 1 mm quartz cuvette between 190 and 600 nm with the instrument set as follows: band width 1 nm, data pitch 1 nm, scanning speed 100 nm/min, 1 s response time at 5 °C. Each CD spectrum was obtained by averaging 8 scans and subtracting the background signal

of buffer and cuvette. For thermal response experiments, the CD signal at 222 nm wavelength was monitored over the temperature range 5 – 95 °C at a ramp rate of 60 °C per hour, with the same settings and peptide concentrations given above. The spectra were converted from ellipticities (mdeg) to mean residue ellipticities (MRE, (deg×cm^2×dmol^{-1}×res^{-1})) by normalising for the concentration of peptide bonds and the cell path length using equation 1:

$$MRE = \frac{\theta x 10^6}{cxlxn}$$

Equation 1: where the variable θ is the measured difference in absorbed circularly polarized light in millidegrees, c is the µM concentration of the compound, l is the path length of the cuvette in mm, and n is the number of amide bonds in the polypeptide.

Analytical Ultracentrifugation

Analytical ultracentrifugation (AUC) was performed on a Beckman Optima X-LA or X-LI analytical ultracentrifuge with an An-50-Ti or An-60-Ti rotor (Beckman-Coulter). Buffer densities, viscosities and peptide and protein partial specific volumes ($\overline{\nu}$) were calculated using SEDNTERP (http://rasmb.org/sednterp/). For sedimentation velocity (SV) experiments, peptide samples were prepared in PBS at 75 µM peptide concentration and placed in a sedimentation velocity cell with 2-channel centrepiece and quartz windows. The samples were centrifuged at 50 krpm at 20 °C, with absorbance scans taken over a radial range of 5.8 – 7.3 cm at 5 min intervals to a total of 120 scans. Data from a single run were fitted to a continuous c(s) distribution model using SEDFIT[5] at a 95% confidence level. Residuals for sedimentation velocity experiments are shown as a bitmap in which the grayscale shade indicates the difference between the fit and raw data (residuals < -0.05 black, > 0.05 white). Good fits are uniformly grey without major dark or light streaks. Sedimentation equilibrium (SE) experiments were performed at 50 µM peptide concentration in 110 µL at 20 °C. The experiment was run in triplicate in a six-channel centrepiece. The samples were centrifuged at speeds in the range of 20 – 45 krpm and scans at each recorded speed were duplicated after equilibration for 8 hours. Data were fitted using SEDPHAT[6] to a single species model. Monte Carlo analysis was performed to give 95% confidence limits.

Fluorescence Measurements – CC-Di Kinetics

Kinetic traces of CC-Di exchange were measured using a Jasco FP-6500 spectrofluorometer with a Julabo F12 temperature controller set at the desired temperature. Fluorescence was measured at an excitation wavelength of 495 nm (± 2 nm) and an emission wavelength of 550 nm (± 2 nm). Time-course measurements were carried out for up to 10,000 seconds or until an end to the rise in fluorescence had been reached where possible. Stocks of labelled and unlabelled peptides were maintained at the experiment temperature in a Grant-bio thermo-shaker. 100 µL of the stock of labelled peptide was added to a quartz cuvette and allowed to equilibrate; at this point, the fluorescence measurement was started. Once the

fluorescence of the labelled stock had equilibrated, an equal volume of the unlabelled peptide stock was added to the solution and mixed thoroughly in the cuvette; after this, the lid was closed, and the fluorescence time course measurement resumed. The curves were fit to either a single exponent (equation 1).

$$f(t)=A(1-\exp(k_{obs}\cdot t))$$

Equation 1: where t is time, A is the pre-exponential factor and k_{obs} is the observed rate.

Our preferred method to interpret the data used single-exponential fitting. We recognise that this is a choice of kinetic model that may not account for the full complexity of the exchange pathways. And, we propose a mechanism for exchange that is consistent with this model. To test the validity of our single-exponential fits, we plotted the residual errors of the fitted data. These do reveal some small deviations from the fitted functions (see examples below for some of the most significant deviations). However, because the deviations of the residuals from the mean are small overall, the amplitude of an additional exponential term in bi-exponential fits would only be a few percent of the main exponential decay term; including an extra fit function would not change the extracted kinetics much. Therefore, we chose to use the minimum number of exponential fit functions that give a good account of the experimental data.

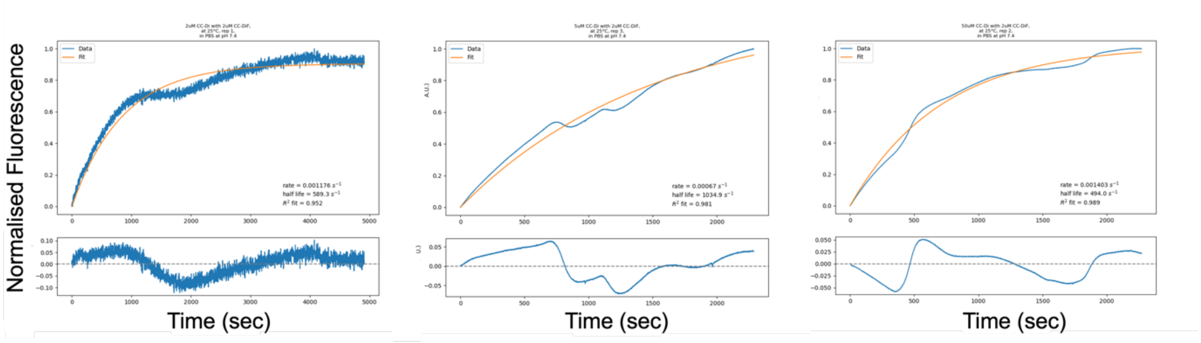

Activation energy of CC-Di was calculated using Equation 2.

$$k=A\cdot exp(^{-E_A}/_{RT})$$

Equation 2: The Arrhenius equation, where *k* is the rate constant, A is the pre-exponential factor, E_A(J mol^{-1}) is the activation energy, R is the molar gas constant (J $mol^{-1}K^{-1}$), and T is the temperature (K).

All measurements were replicated 3 times.

Data were processed and fit to equation 2 using the code in a Jupyter notebook. This code is available on the Woolfson lab github (http://github.com/woolfson-group/CC_exchange)

Fluorescence Measurements – Screen of Hendecad-Incorporated Peptides

Samples of 1:10 FAM-labelled peptide assembly:unlabelled peptide assembly were prepared in PBS buffer. The mixtures were kept in the dark and incubated at 25 °C. At 1hr, 24hr, and post-annealing time points, 200 µL of sample was transferred to 1 cm quartz

cuvettes and fluorescence measurements were conducted using a Jasco FP-6500 spectrofluorometer with a Julabo F12 temperature controller set to 25 °C. Samples were excited at 495 nm (bandwidth = 1 nm) and emission was measured from 510-700 nm (bandwidth = 3 nm) with a data pitch of 1 nm. Response time was set to 1 sec, PMT Voltage was set to 500 V, and scanning speed was set to 200 nm/min. Three 510-700 nm scans were acquired for each sample and averaged. Each measurement was performed three times.

Fluorescence Measurements – Plate Reader

Samples of 1:10 FAM-labelled peptide assembly:unlabelled peptide assembly were prepared in PBS buffer. The mixtures were kept in the dark and incubated at 25 °C. At 1hr, 24hr, and post-annealing time points, 100 µL of sample was transferred to black 96-well plates suitable for fluorescence measurements. Fluorescence was measured using a BMG Labtech (Aylesbury, UK) Clariostar plate reader set to 25 °C. Samples were excited at 483 (+/- 14 nm) and emission measured at 530 (+/- 30 nm) with dichroic filter 502.5. For all measurements the gain was set to 500 and the focal height to 5.7 mm. All experiments were performed 3 times.

All data were normalised to the values corresponding to the FAM-labelled peptide where the annealed FAM-labelled peptide in buffer is set to zero and the annealed homotypic exchange of the FAM-labelled peptide set to one. For example, all data acquired of samples prepared with FAM-CC-Di and unlabelled peptide were normalised against the averaged fluorescence values of annealed FAM-CC-Di in buffer, set to zero, and the averaged fluorescence values of annealed FAM-CC-Di+CC-Di, set to one.

Peptide Crystallization

Diffraction-quality peptide crystals were grown using a sitting- drop vapor-diffusion method. Commercially available sparse matrix screens were used (Morpheus®, JCSG-plus™, Structure Screen 1 and 2, Pact Premier™, ProPlex™; Molecular Dimensions), and the drops were dispensed using a robot (Oryx8; Douglas Instruments). For each well of an MRC 2 drop plate, 0.3 µL of peptide (8 mg/mL) and 0.3 µL of reservoir solution in parallel with 0.4 µL of the peptide and 0.2 µL of reservoir solution were mixed and the plate was incubated at 20 °C. Crystals of CC-Hex2-hen2, Ala at ***h***, grown in 0.3 M magnesium formate dihydrate and 0.1 M BIS_TRIS pH 5.5, CC-Hept-hen2, Ala at ***h***, grown in 1 M sodium citrate, pH 5.5 and 20 % w/v PEG 3000, and CC-Hept-IV-hen2 grown in 1.5 M ammonium sulphate and 0.1 M sodium acetate, pH 5.0 were looped and soaked in a reservoir containing 25% (v/v) glycerol as a cryoprotectant.

X-Ray Data Collection and Structure determination.

Diffraction data for the crystals were obtained at the Diamond Light Source (Didcot, UK) on beamlines I04 and I24. Data were processed using the automated pipelines: Xia2 pipelines[7], which ports data through DIALS[8] or MOSFLM[9] to POINTLESS and AIMLESS[10] as implemented in the CCP4 suite[11], or XDS to XSCALE[12]; or the AUTOPROC pipelines, which

use the same integrating and data reduction software in addition to STARANISO.[13] The data acquired for the CC-Hex2-hen2, Ala at ***h***, were phased using either *ab initio* phasing using ARCIMBOLDO_LITE[14,15] to generate a model for molecular replacement using PHASER.[16] A phenylalanine version of the AlphaFold2[17] model of CC-Hept-IV-hen2 was used as the search model to phase the CC-Hept-IV-hen2 data using molecular replacement using PHASER.[16] Initial models were built from the initial solution using BUCCANEER.[18] Final structures were obtained after iterative rounds of model building with COOT[19] and refinement with REFMAC5.[20]

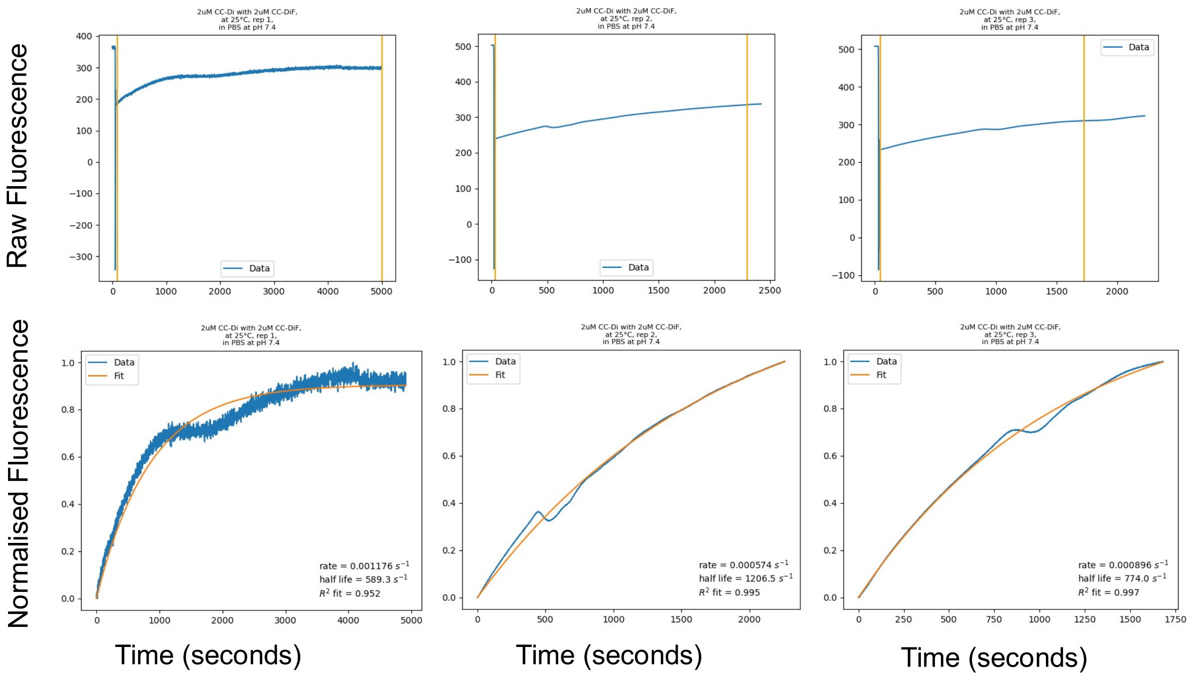

Figure S1. Time course data and fits for the exchange of CC-Di (2 μM) and labelled CC-Di (2 μM), replicates 1-3. (Top) Raw fluorescence (A.U.) time course data for the exchange with orange lines showing where the raw time course was trimmed for the fit to the single exponent. (Bottom) Normalised fluorescence data (A.U.) (blue) and single-exponential fits (orange).

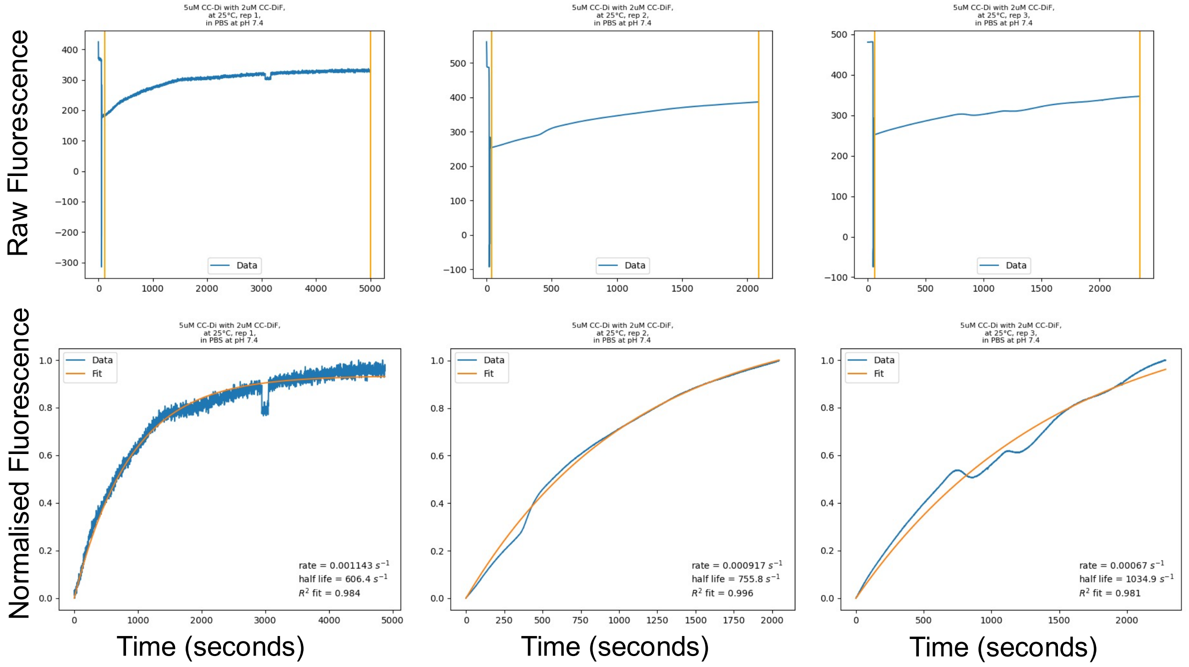

Figure S2. Time course data and fits for the exchange of CC-Di (5 μM) and labelled CC-Di (2 μM), replicates 1-3. (Top) Raw fluorescence (A.U.) time course data for the exchange with orange lines showing where the raw time course was trimmed for the fit to the single exponent. (Bottom) Normalised fluorescence data (A.U.) (blue) and single-exponential fits (orange).

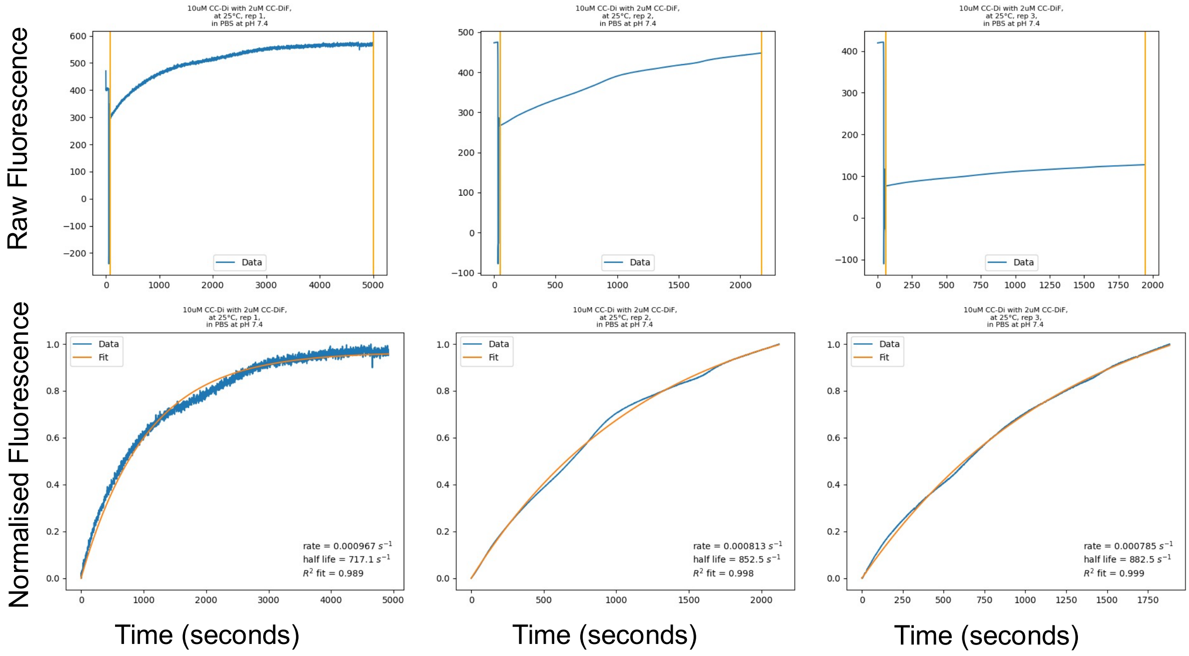

Figure S3. Time course data and fits for the exchange of CC-Di (10 μM) and labelled CC-Di (2 μM), replicates 1-3. (Top) Raw fluorescence (A.U.) time course data for the exchange with orange lines showing where the raw time course was trimmed for the fit to the single exponent. (Bottom) Normalised fluorescence data (A.U.) (blue) and single-exponential fits (orange).

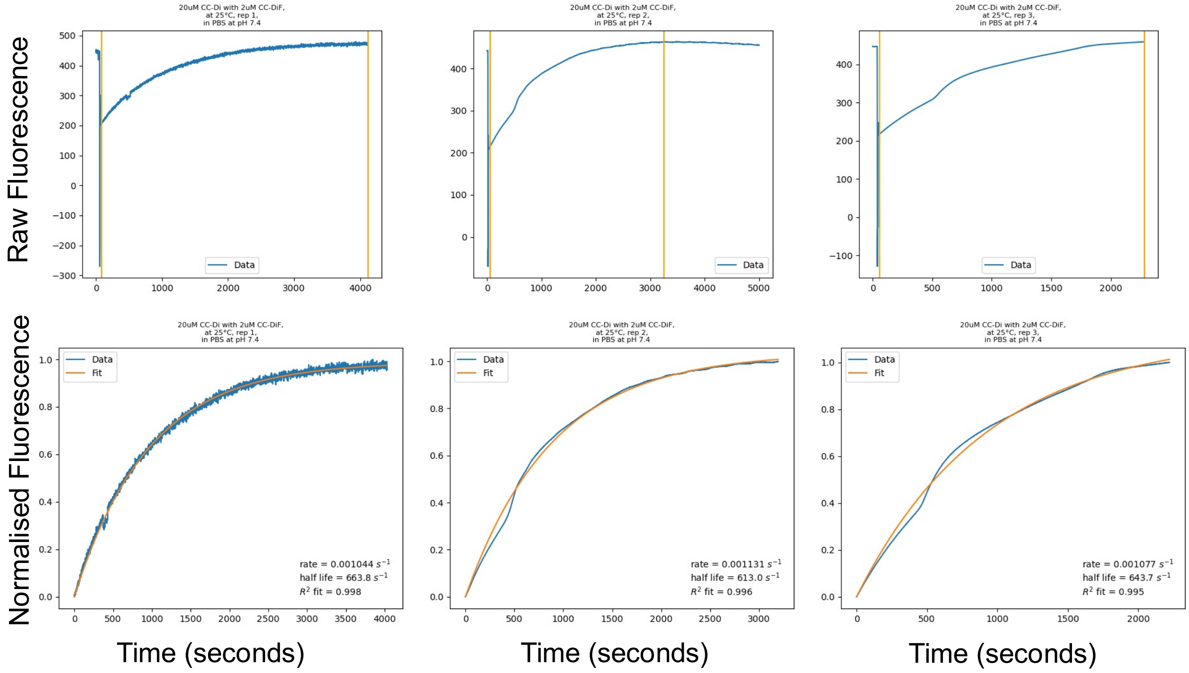

Figure S4. Time course data and fits for the exchange of CC-Di (20 μM) and labelled CC-Di (2 μM), replicates 1-3. (Top) Raw fluorescence (A.U.) time course data for the exchange with orange lines showing where the raw time course was trimmed for the fit to the single exponent. (Bottom) Normalised fluorescence data (A.U.) (blue) and single-exponential fits (orange).

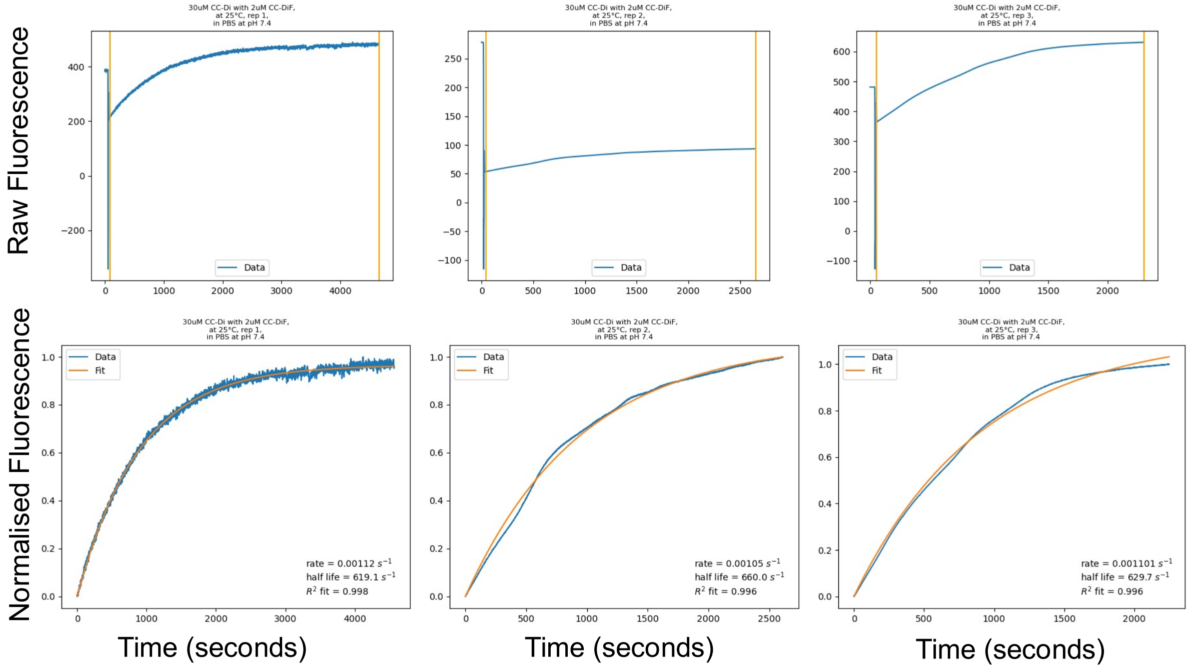

Figure S5. Time course data and fits for the exchange of CC-Di (30 μM) and labelled CC-Di (2 μM), replicates 1-3. (Top) Raw fluorescence (A.U.) time course data for the exchange with orange lines showing where the raw time course was trimmed for the fit to the single exponent. (Bottom) Normalised fluorescence data (A.U.) (blue) and single-exponential fits (orange).

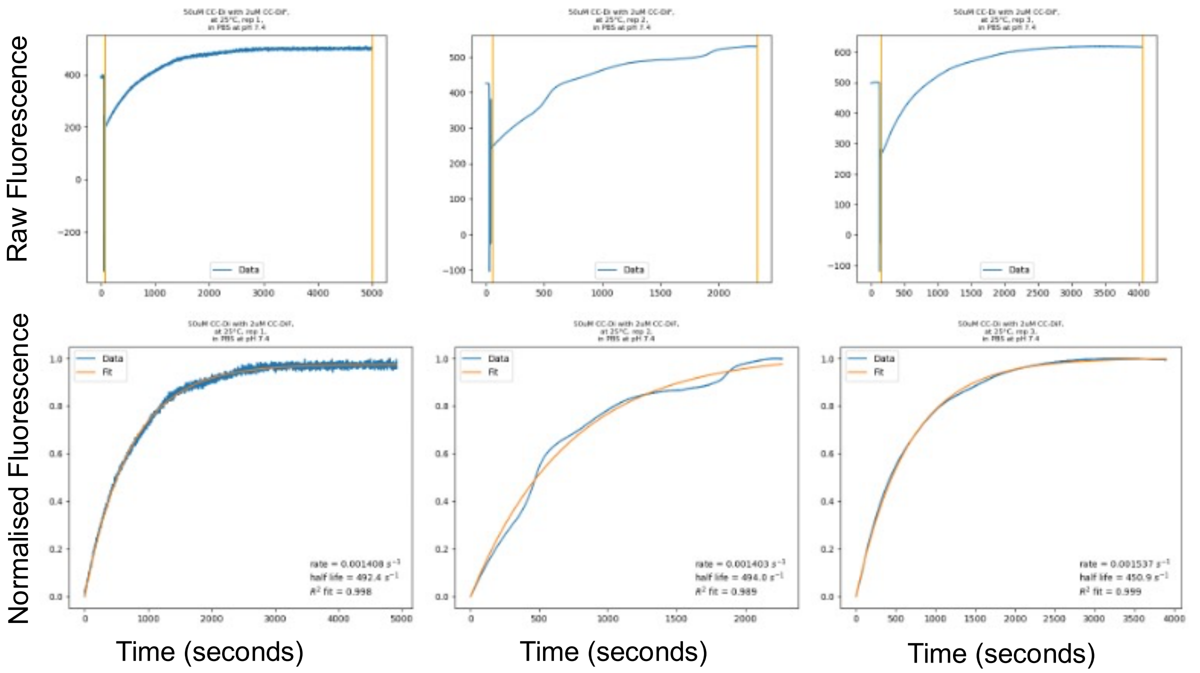

Figure S6. Time course data and fits for the exchange of CC-Di (50 µM) and labelled CC-Di (2 µM), replicates 1-3. (Top) Raw fluorescence (A.U.) time course data for the exchange with orange lines showing where the raw time course was trimmed for the fit to the single exponent. (Bottom) Normalised fluorescence data (A.U.) (blue) and single-exponential fits (orange).

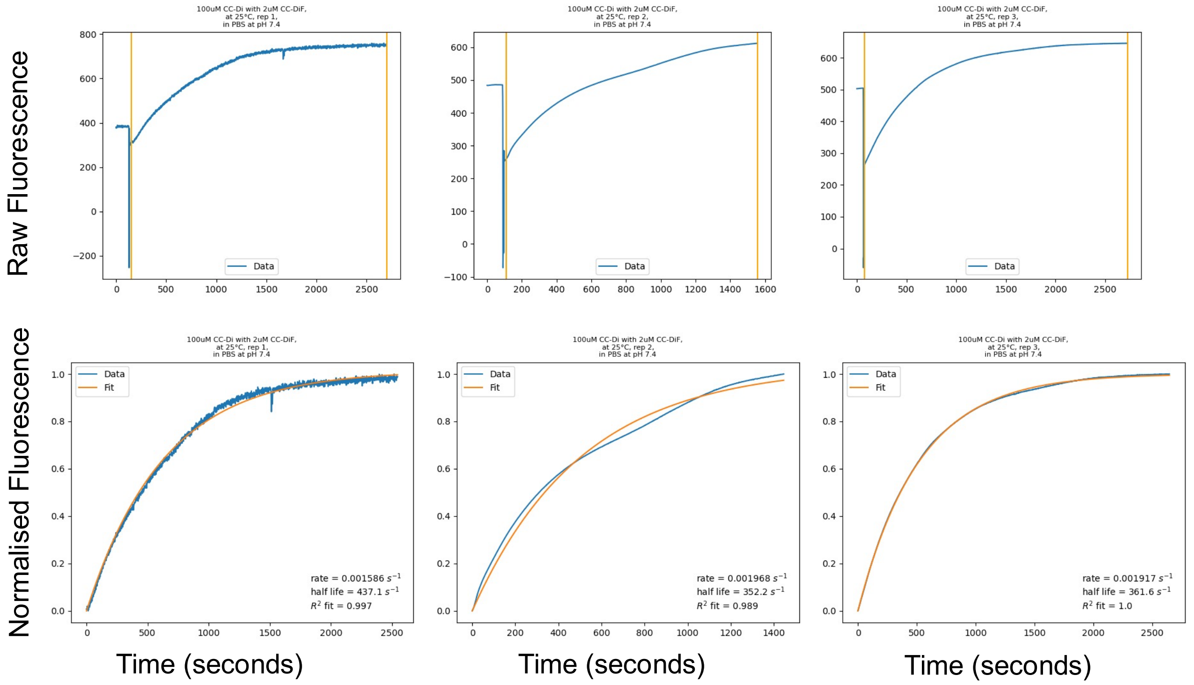

Figure S7. Time course data and fits for the exchange of CC-Di (100 µM) and labelled CC-Di (2 µM), replicates 1-3. (Top) Raw fluorescence (A.U.) time course data for the exchange with orange lines showing where the raw time course was trimmed for the fit to the single exponent. (Bottom) Normalised fluorescence data (A.U.) (blue) and single-exponential fits (orange).

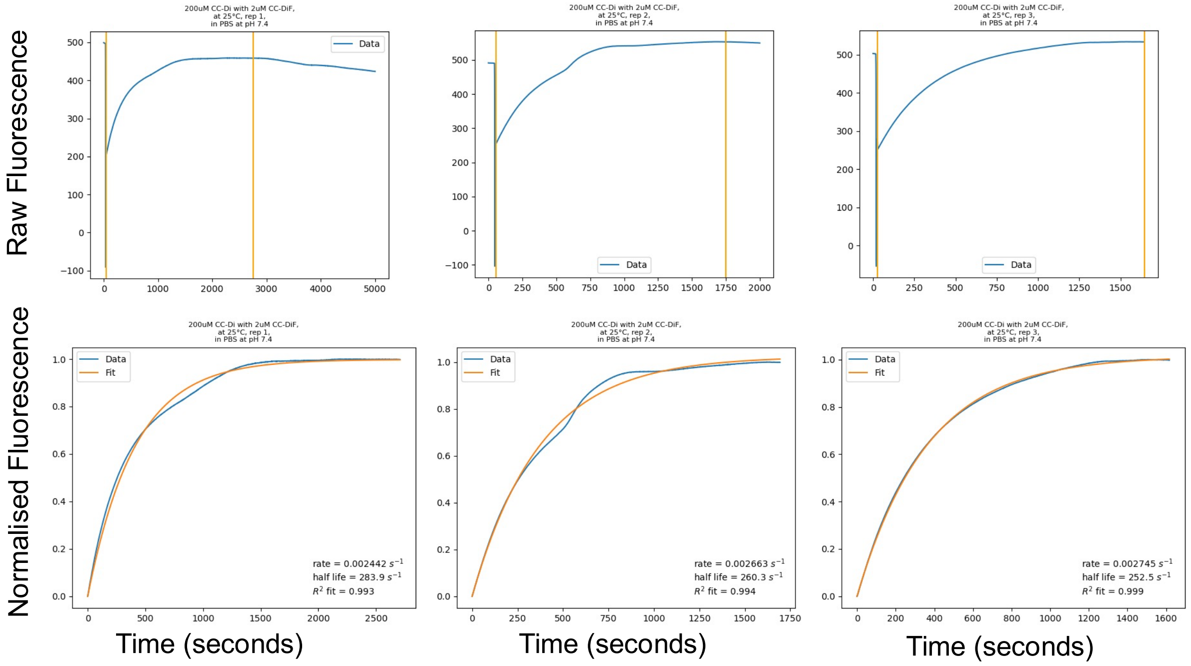

Figure S8. Time course data and fits for the exchange of CC-Di (200 µM) and labelled CC-Di (2 µM), replicates 1-3. (Top) Raw fluorescence (A.U.) time course data for the exchange with orange lines showing where the raw time course was trimmed for the fit to the single exponent. (Bottom) Normalised fluorescence data (A.U.) (blue) and single-exponential fits (orange).

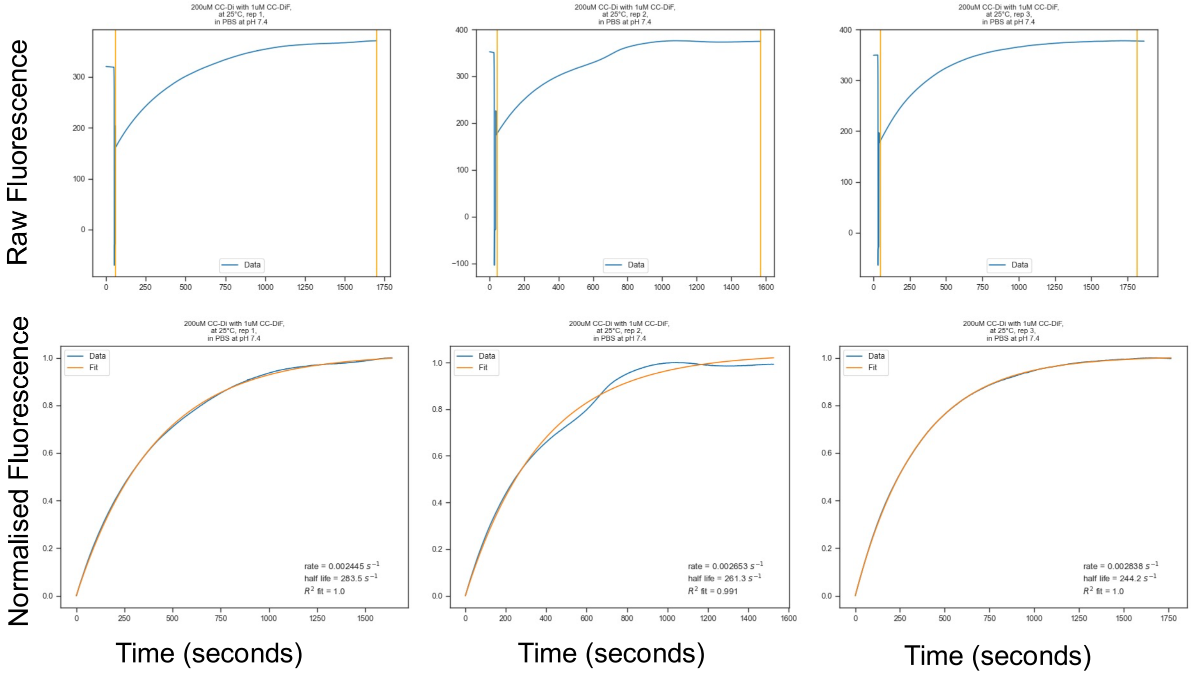

Figure S9. Time course data and fits for the exchange of CC-Di (200 µM) and labelled CC-Di (1 µM), replicates 1-3. (Top) Raw fluorescence (A.U.) time course data for the exchange with orange lines showing where the raw time course was trimmed for the fit to the single exponent. (Bottom) Normalised fluorescence data (A.U.) (blue) and single-exponential fits (orange).

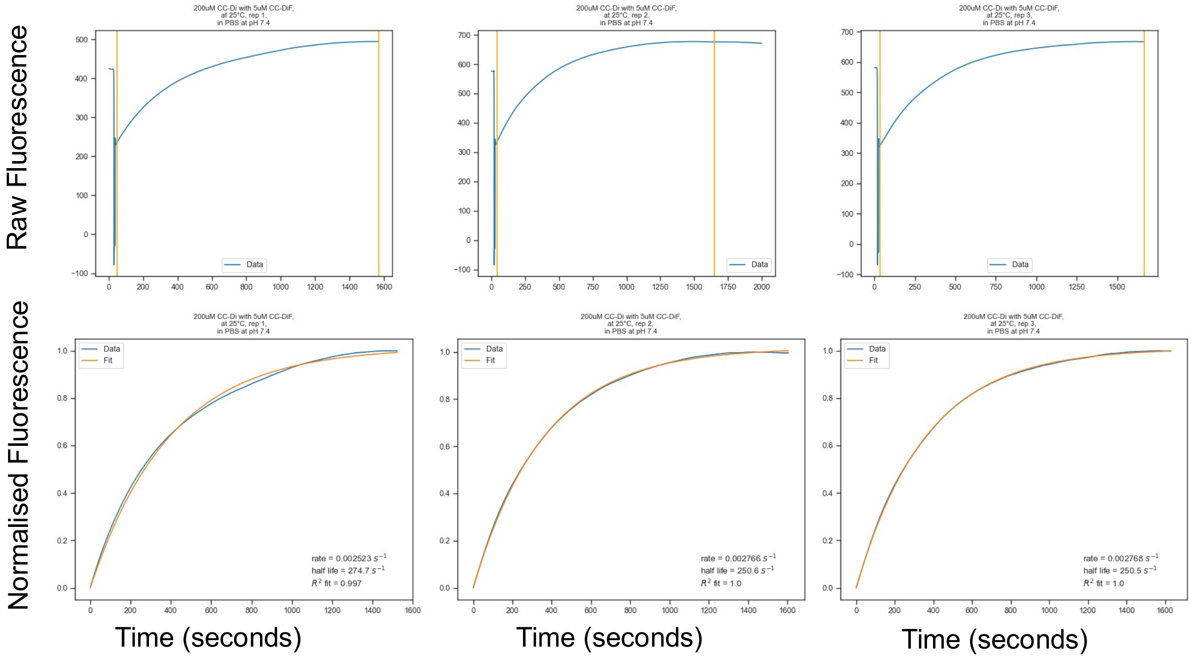

Figure S10. Time course data and fits for the exchange of CC-Di (200 µM) and labelled CC-Di (5 µM), replicates 1-3. (Top) Raw fluorescence (A.U.) time course data for the exchange with orange lines showing where the raw time course was trimmed for the fit to the single exponent. (Bottom) Normalised fluorescence data (A.U.) (blue) and single-exponential fits (orange).

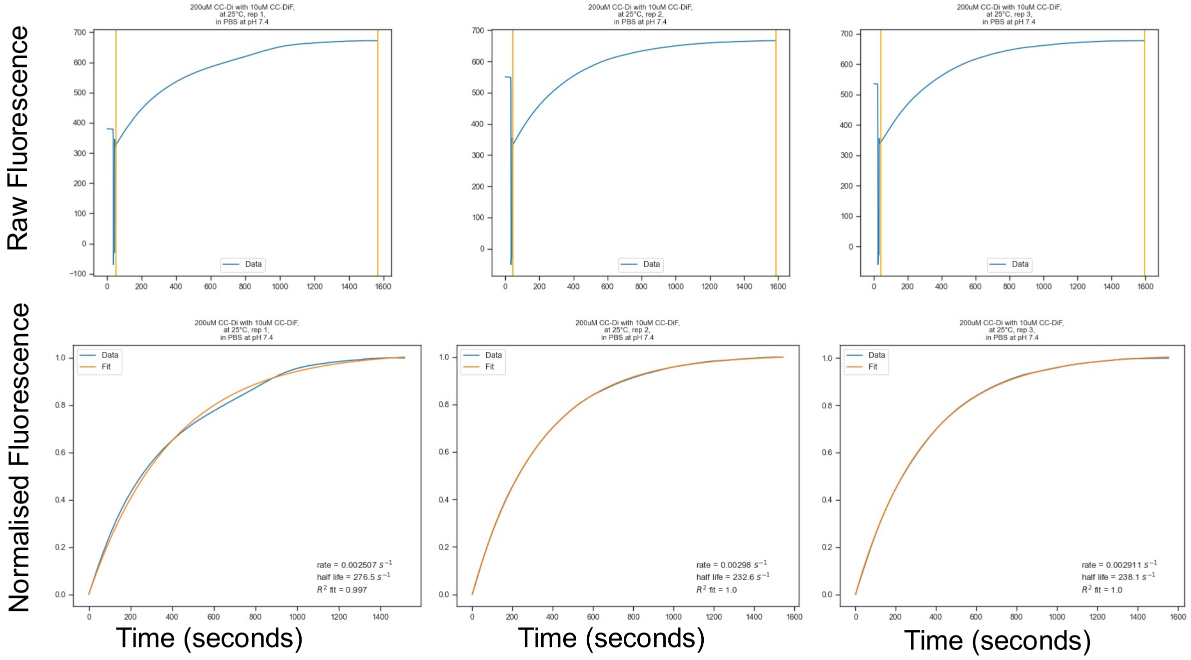

Figure S11. Time course data and fits for the exchange of CC-Di (200 µM) and labelled CC-Di (10 µM), replicates 1-3. (Top) Raw fluorescence (A.U.) time course data for the exchange with orange lines showing where the raw time course was trimmed for the fit to the single exponent. (Bottom) Normalised fluorescence data (A.U.) (blue) and single-exponential fits (orange).

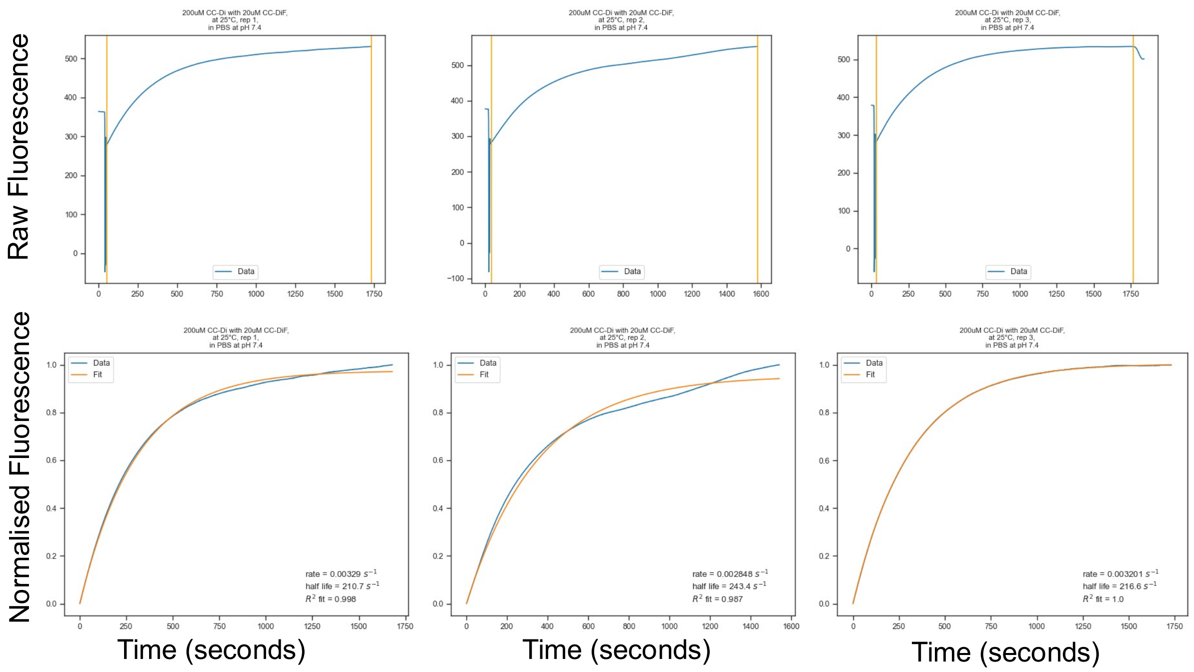

Figure S12. Time course data and fits for the exchange of CC-Di (200 μM) and labelled CC-Di (20 μM), replicates 1-3. (Top) Raw fluorescence (A.U.) time course data for the exchange with orange lines showing where the raw time course was trimmed for the fit to the single exponent. (Bottom) Normalised fluorescence data (A.U.) (blue) single-exponential fits (orange).

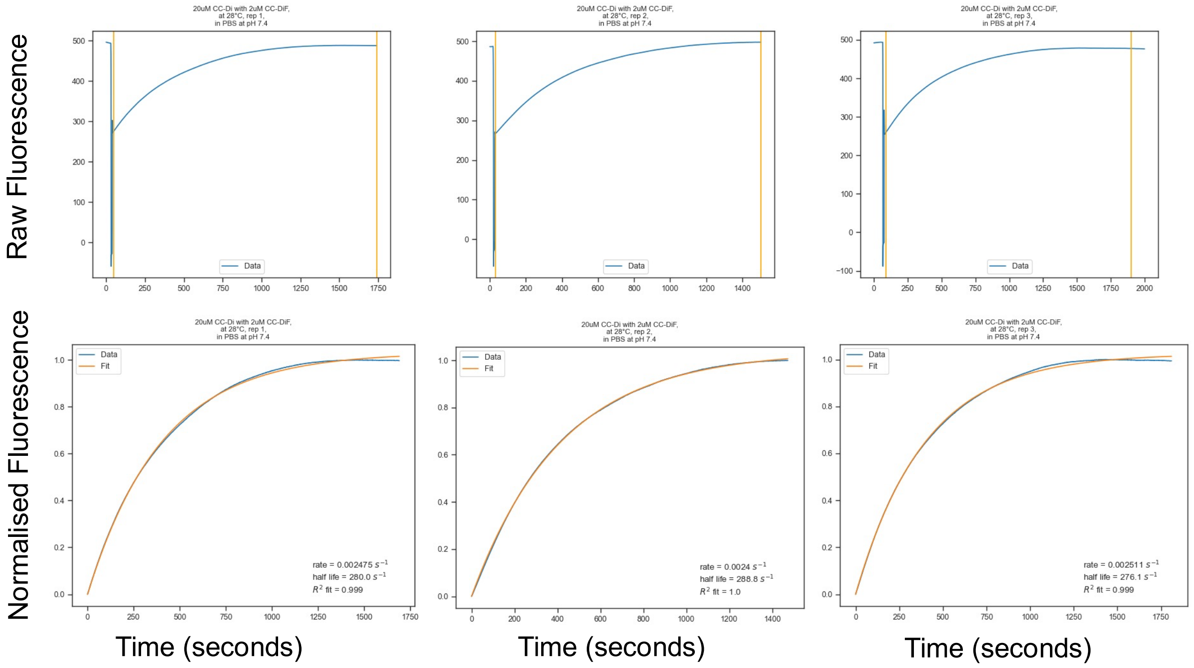

Figure S13. Time course data and fits for the exchange of CC-Di (20 μM) and labelled CC-Di (2 μM), replicates 1-3 at 28 °C. (Top) Raw fluorescence (A.U.) time course data for the exchange with orange lines showing where the raw time course was trimmed for the fit to the single exponent. (Bottom) Normalised fluorescence data (A.U.) (blue) and single-exponential fits (orange).

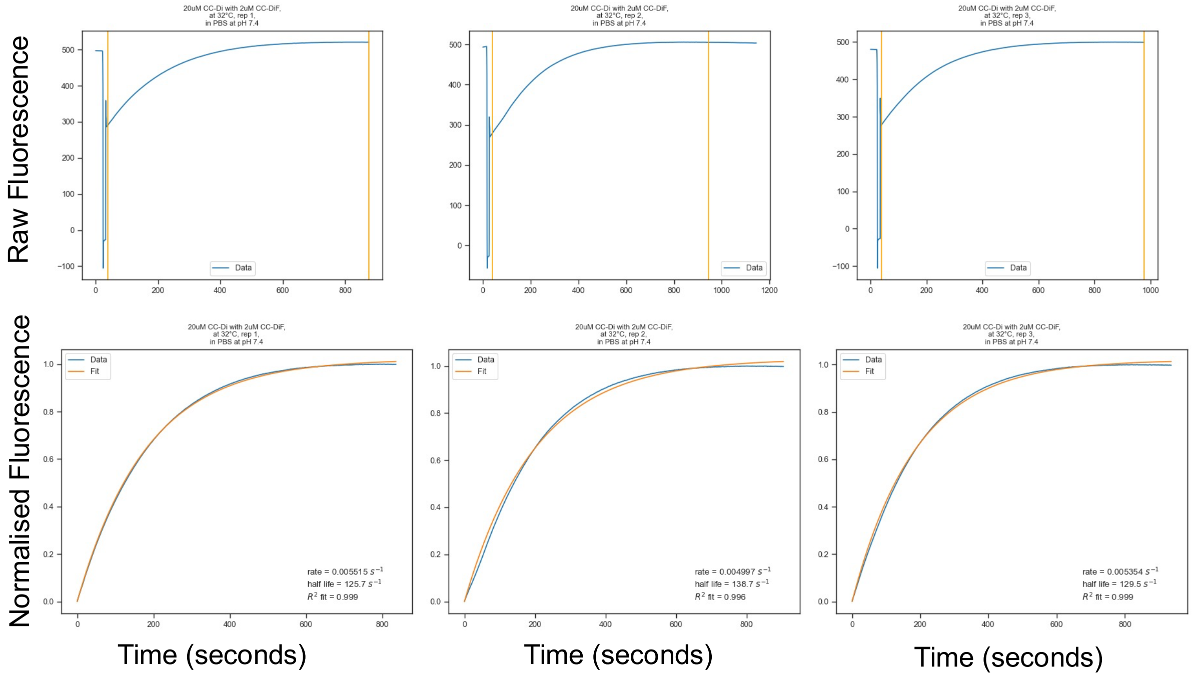

Figure S14. Time course data and fits for the exchange of CC-Di (20 µM) and labelled CC-Di (2 µM), replicates 1-3 at 32 °C. (Top) Raw fluorescence (A.U.) time course data for the exchange with orange lines showing where the raw time course was trimmed for the fit to the single exponent. (Bottom) Normalised fluorescence data (A.U.) (blue) and single-exponential fits (orange).

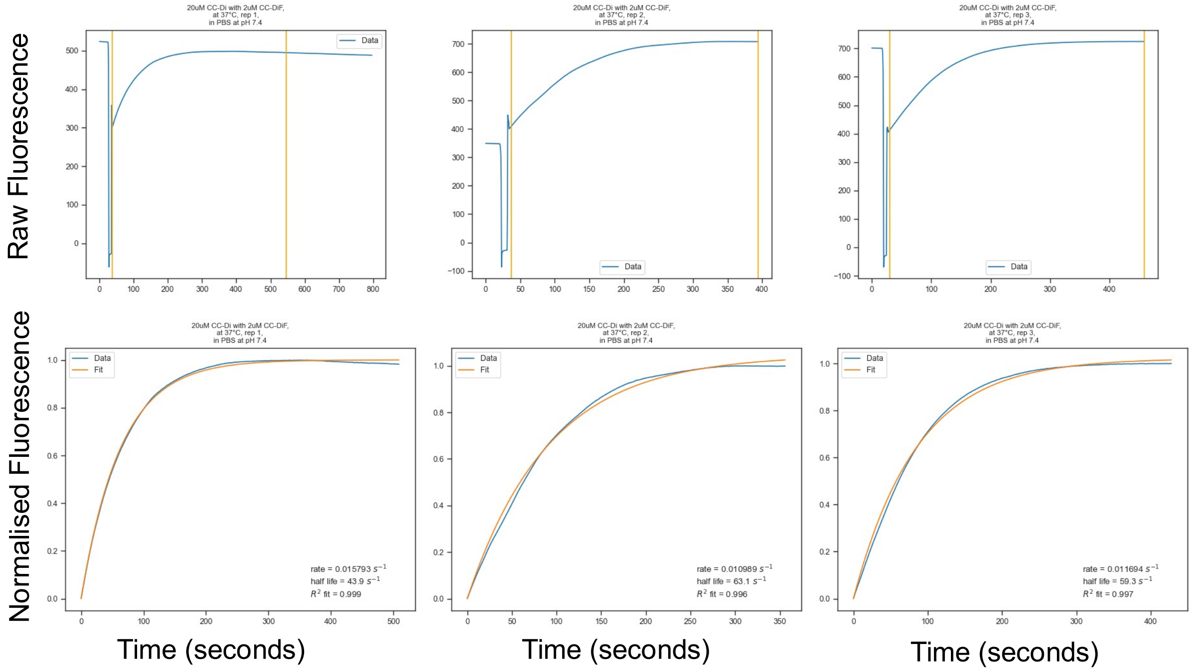

Figure S15. Time course data and fits for the exchange of CC-Di (20 µM) and labelled CC-Di (2 µM), replicates 1-3 at 37 °C. (Top) Raw fluorescence (A.U.) time course data for the exchange with orange lines showing where the raw time course was trimmed for the fit to the single exponent. (Bottom) Normalised fluorescence data (A.U.) (blue) and single-exponential fits (orange).

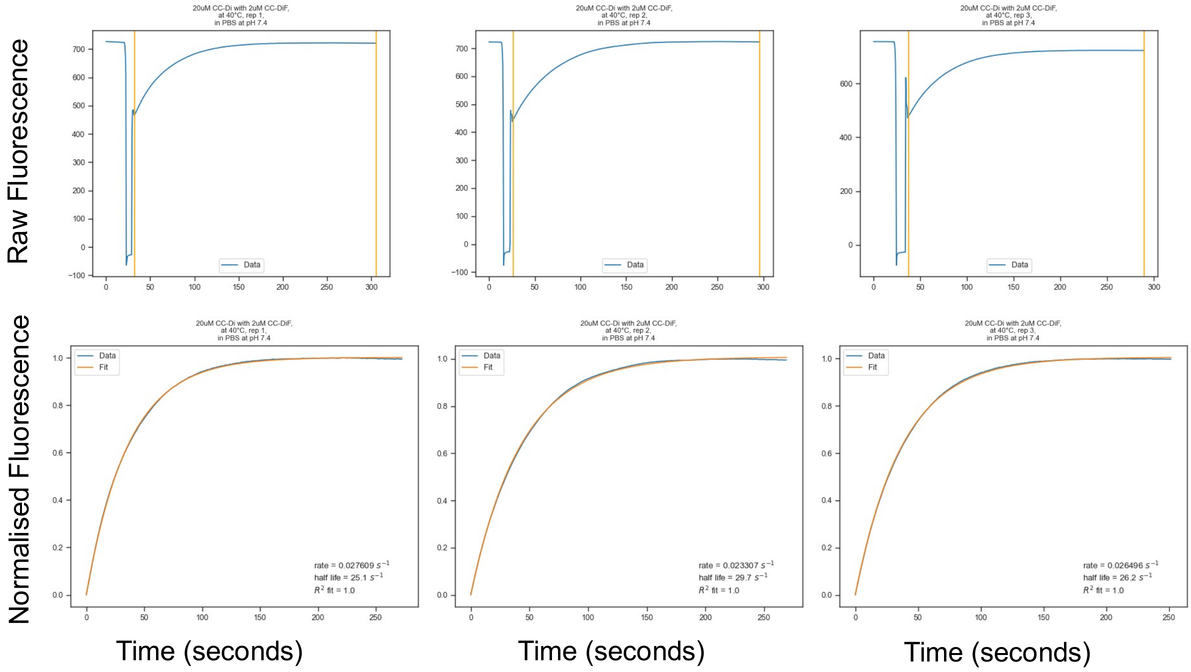

Figure S16. Time course data and fits for the exchange of CC-Di (20 µM) and labelled CC-Di (2 µM), replicates 1-3 at 40 °C. (Top) Raw fluorescence (A.U.) time course data for the exchange with orange lines showing where the raw time course was trimmed for the fit to the single exponent. (Bottom) Normalised fluorescence data (A.U.) (blue) and single-exponential fits (orange).

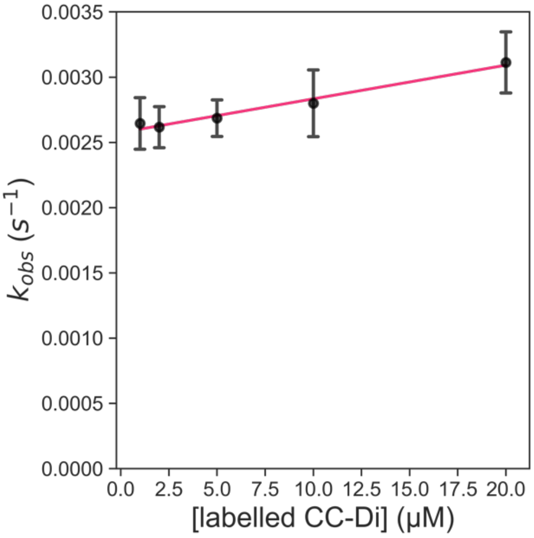

Figure S17. Plot of the rate (k_{obs}) of exchange of 200 µM unlabelled CC-Di with variable concentrations of labelled CC-Di (1-20 µM**).** Data points are shown as the average of 3 independent replicates, error bars are shown to 1 standard deviation, and the line of best fit is also shown.

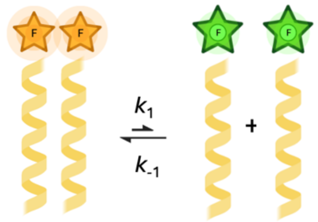

Scheme S1. Scheme of FAM-CC-Di dissociation.

If $[L]$ is defined as the concentration of dissociated, monomeric FAM-CC-Di and $[LL]$ is defined as the concentration of folded dimeric FAM-CC-Di at equilibrium and the overall concentration of FAM-CC-Di is 20 µM then:
At equilibrium, $K = \frac{[L]^2}{[LL]}$

**Total Concentration FAM-CC-Di (µM)**	1	2	5	10	20
**Total Concentration CC-Di (µM)**	200	200	200	200	200
$[L]$ (µM)	$8.13\text{x}10^{-3}$	$1.15\text{x}10^{-2}$	$1.82\text{x}10^{-2}$	$2.58\text{x}10^{-2}$	$3.65\text{x}10^{-2}$
$[LL]$ (µM)	0.992	1.99	4.98	9.97	20.0
$[U]$ (µM)	$1.15\text{x}10^{-1}$	$1.15\text{x}10^{-1}$	$1.15\text{x}10^{-1}$	$1.15\text{x}10^{-1}$	$1.15\text{x}10^{-1}$
$[UU]$ (µM)	200	200	200	200	200

K = 6.67 x10^{-11},[1] from which the degree of dissociation is deduced to be 0.00182 at 20 µM concentration, therefore $[L]$ = 0.0365 µM and $[LL]$ = 20.0 µM

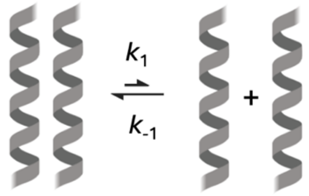

Scheme S2. Scheme of CC-Di dissociation.

If $[U]$ is defined as the concentration of dissociated, monomeric CC-Di and $[UU]$ is defined as the concentration of folded dimeric CC-Di at equilibrium, and the overall concentration of CC-Di is 200 µM then then:
At equilibrium, $K = \frac{[U]^2}{[UU]}$
K = 6.67 x10^{-11}, from which the degree of dissociation is deduced to be 0.00058 at 200 µM concentration, therefore $[U]$ = 0.115 µM and $[UU]$ =200 µM

Table S1. Peptide concentration of dissociated monomeric ($[L]$, $[U]$) and folded dimeric ($[LL]$, $[UU]$) FAM-CC-Di and CC-Di at experimental conditions where the total concentration of unlabelled peptide was constant and the concentration of labelled peptide varied.

Table S2. Peptide concentration of dissociated monomeric ($[L]$, $[U]$) and folded dimeric ($[LL]$, $[UU]$) FAM-CC-Di and CC-Di at experimental conditions where the total concentration of labelled peptide was constant and the concentration of unlabelled peptide varied.

**Total Concentration**	2	2	2	2	2	2	2	20

**FAM-CC-Di (µM)**								
**Total Concentration CC-Di (µM)**	2	5	10	20	30	50	100	200
[*L*] (µM)	$1.15x10^{-2}$	$1.15x10^{-2}$	$1.15x10^{-2}$	$1.15x10^{-2}$	$1.15x10^{-2}$	$1.15x10^{-2}$	$1.15x10^{-2}$	$3.65x10^{-2}$
[*LL*] (µM)	1.99	1.99	1.99	1.99	1.99	1.99	1.988	20.0
[*U*] (µM)	$1.15x10^{-2}$	$1.82x10^{-2}$	$2.58x10^{-2}$	$3.65x10^{-2}$	$4.47x10^{-2}$	$5.77x10^{-2}$	$8.16x10^{-2}$	$1.15x10^{-1}$
[*UU*] (µM)	1.99	4.98	9.97	20.0	30.0	59.9	99.9	200

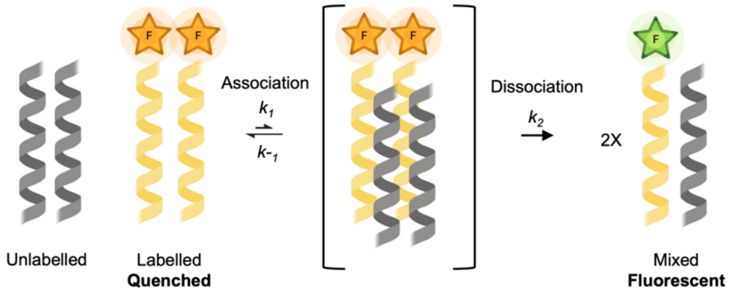

Scheme S3. Proposed mechanism 1 of exchange of FAM-CC-Di and CC-Di.

The rate of growth of fluorescence is the same as the rate of growth of dimers consisting of 1 copy of FAM-CC-Di and one copy of CC-Di (LU).

$$\text{Rate} = \frac{d[UL]}{dt} = 2k_2[UULL]$$

Applying the steady-state approximation to the tetramer:

$$\frac{d[UULL]}{dt} = k_1[UU][LL] - k_{-1}[UULL] - k_2[UULL] = 0$$

So

$$[UULL] = \frac{k_1}{k_{-1}+k_2}[UU][LL]$$

And

$$\text{Rate} = \frac{d[UL]}{dt} = \frac{2k_2k_1}{k_{-1}+k_2}[UU][LL]$$

If $[UU]$ is much greater than $[LL]$ then pseudo-first-order kinetics apply and we can write:

$$\text{Rate} = \frac{d[UL]}{dt} = k_{obs}[LL]$$

Where the pseudo-first-order rate constant k_{obs} is:

$$k_{obs} = \frac{2k_2k_1}{k_{-1}+k_2}[UU]$$

Hence a plot of k_{obs} vs $[UU]$ will be linear with gradient $\frac{2k_2k_1}{k_{-1}+k_2}$.

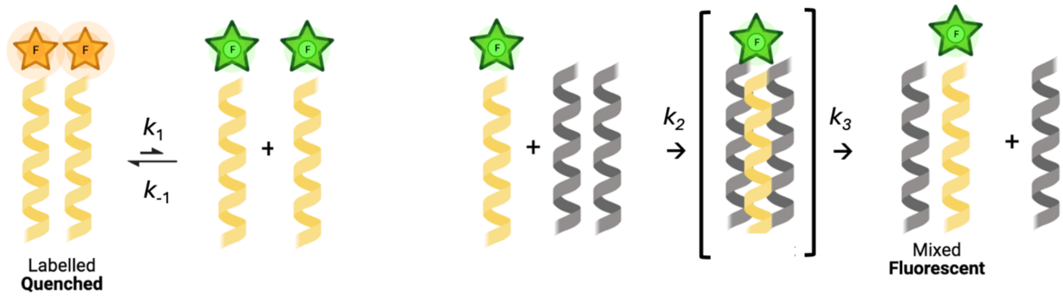

Scheme S4. Proposed mechanism 2 of exchange of FAM-CC-Di and CC-Di.

The rate of growth of fluorescence is the same as the rate of growth of dimers consisting of 1 copy of FAM-CC-Di and one copy of CC-Di (LU) if fluorescence from dissociated monomeric FAM-CC-Di and from the trimeric steady-state intermediate is negligible. This is likely accurate as the monomeric and trimeric species are unstable and only present at low concentrations at any given time.

$$\text{Rate} = \frac{d[LU]}{dt} = k_3[LUU]$$

We can also write

$$\frac{d[LUU]}{dt} = k_2[L][UU] - k_3[LUU]$$

and apply the steady state approximation to the intermediate so

$$\frac{d[LUU]}{dt} = 0$$

Hence $k_2[L][UU] = k_3[LUU]$ and

$$\text{Rate} = \frac{d[LU]}{dt} = k_2[L][UU]$$

Which is first order in $[UU]$ and $[L]$.

If we assume the dissociation of dimeric FAM-CC-Di is at equilibrium, then the equilibrium constant is $K = [L]^2/[LL]$ and hence $[L] = K^{1/2}\,[LL]^{1/2}$. Substituting to the rate expression gives:

$$\text{Rate} = \frac{d[LU]}{dt} = k_2\,K^{1/2}[LL]^{1/2}\,[UU]$$

Under experimental conditions in which $[UU] >> [LL]$, we can write:

$$\text{Rate} = \frac{d[LU]}{dt} = k_{obs}\,[LL]^{1/2}$$

Where $k_{obs} = k_2 K^{1/2}\,[UU]$. Hence a plot of k_{obs} vs $[UU]$ should be linear (Figure 1C) with gradient $k_2 K^{1/2}$.

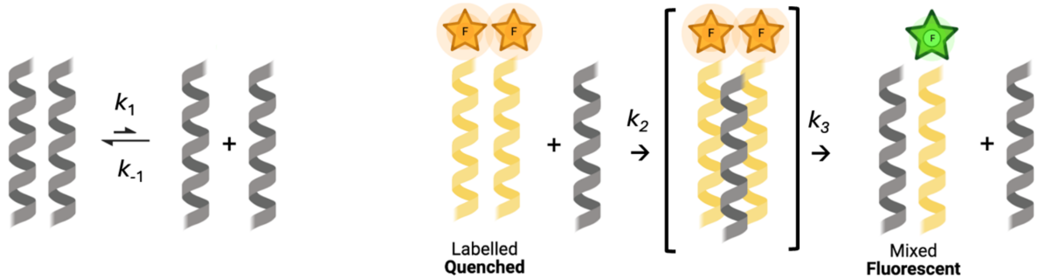

Scheme S5. Proposed mechanism 3 of exchange of FAM-CC-Di and CC-Di.

The rate of growth of fluorescence is the same as the rate of growth of dimers consisting of 1 copy of FAM-CC-Di and one copy of CC-Di (LU).

$$\text{Rate} = \frac{d[LU]}{dt} = k_3[LLU]$$

We can also write

$$\frac{d[LLU]}{dt} = k_2[LL][U] - k_3[LLU]$$

and apply the steady state approximation to the intermediate so

$$\frac{d[LLU]}{dt} = 0$$

Hence $k_2[LL][U] = k_3[LLU]$ and

$$\text{Rate} = \frac{d[LU]}{dt} = k_2[LL][U]$$

Which is first order in $[UU]$ and $[L]$.

If we assume the dissociation of dimeric CC-Di is at equilibrium, then the equilibrium constant is $K = [U]^2/[UU]$ and hence $[U] = K^{1/2}\,[UU]^{1/2}$. Substituting to the rate expression gives:

$$\text{Rate} = \frac{d[LU]}{dt} = k_2\,K^{1/2}[UU]^{1/2}\,[LL]$$

Under experimental conditions in which $[UU] >> [LL]$, we can write:

$$\text{Rate} = \frac{d[LU]}{dt} = k_{obs}\,[LL]$$

Where $k_{obs} = k_2 K^{1/2} [UU]^{1/2}$. Hence a plot of k_{obs} vs $[UU]^{1/2}$ should be linear with gradient $k_2 K^{1/2}$.

Table S3. Data for the exponential fits for the exchange of CC-Di

Temperature (°C)	[CC-Di] Unlabelled (μM)	[CC-Di] Labelled (μM)	Replicate #	Rate (k_{obs}, s^{-1})	Half-life ($t_{1/2}$, s)	R^2 score of the fit
Data for constant [CC-Di] Unlabelled						
25	200	1	1	0.00244	284	0.999
25	200	1	2	0.00265	261	0.991
25	200	1	3	0.00284	244	0.999
25	200	2	1	0.00244	284	0.993
25	200	2	2	0.00266	260	0.993
25	200	2	3	0.00275	253	0.999
25	200	5	1	0.00252	275	0.997
25	200	5	2	0.00277	251	0.999
25	200	5	3	0.00277	250	0.999
25	200	10	1	0.00251	276	0.996
25	200	10	2	0.00298	233	0.999
25	200	10	3	0.00291	238	0.999
25	200	20	1	0.00329	211	0.997
25	200	20	2	0.00285	243	0.987
25	200	20	3	0.00320	217	0.999
Data for constant [CC-Di] Labelled						
25	2	2	1	0.00118	589	0.952
25	2	2	2	0.000574	1207	0.995
25	2	2	3	0.000896	774	0.997
25	5	2	1	0.00114	606	0.984
25	5	2	2	0.000917	756	0.996
25	5	2	3	0.000670	1035	0.981
25	10	2	1	0.000967	717	0.989
25	10	2	2	0.000813	852	0.998
25	10	2	3	0.000785	883	0.999

25	20	2	1	0.00104	664	0.998
25	20	2	2	0.00113	613	0.996
25	20	2	3	0.00108	644	0.995
25	30	2	1	0.00112	619	0.998
25	30	2	2	0.00105	660	0.996
25	30	2	3	0.00110	630	0.996
25	50	2	1	0.00141	492	0.998
25	50	2	2	0.00140	494	0.989
25	50	2	3	0.00154	451	0.999
25	100	2	1	0.00159	437	0.997
25	100	2	2	0.00197	352	0.989
25	100	2	3	0.00192	362	0.99
25	200	2	1	0.00244	284	0.993
25	200	2	2	0.00266	260	0.994
25	200	2	3	0.00275	253	0.999
Data for the Arrhenius plot						
25	20	2	1	0.00104	664	0.998
25	20	2	2	0.00113	613	0.996
25	20	2	3	0.00108	644	0.995
28	20	2	1	0.00248	280	0.999
28	20	2	2	0.00240	289	0.999
28	20	2	3	0.00251	276	0.999
32	20	2	1	0.00551	126	0.999
32	20	2	2	0.00500	139	0.996
32	20	2	3	0.00535	129	0.999
37	20	2	1	0.0158	43.9	0.999
37	20	2	2	0.0110	63.1	0.996
37	20	2	3	0.0117	59.3	0.997

40	20	2	1	0.0276	25.1	0.999
40	20	2	2	0.0233	29.7	0.999
40	20	2	3	0.0265	26.2	0.999

Table S4. Data for the fit to the Arrhenius equation and subsequent activation energy calculation.

**Temperature (°C)**	**Rate (k_{obs}, s^{-1}) (average of 3 replicates)**	**1/T (K^{-1})**	**ln(k_{obs})**
25	0.00108	0.00335	-6.83
28	0.00246	0.00332	-6.01
32	0.00529	0.00328	-5.24
37	0.0128	0.00322	-4.36
40	0.0258	0.00319	-3.66
Slope (Ea/R)	-19036		
Intercept (lnA)	57.105		
R (molar gas constant) (J K^{-1} mol^{-1})	8.3144598		
Activation Energy (J mol^{-1})	158274		
Activation Energy (kJ mol^{-1})	158.274		
Activation Energy (kcal mol^{-1})	37.8		
R^2 of linear regression	0.996		

Table S5. CC Basis Set Sequences

***g*-Register CC**	***gabcdef gabcdef gabcdef gabcdef***	**Monoistopic Mass**
CC-Di	Ac-G EIAALKQ EIAALKK ENAALKW EIAALKQ G-CONH2	3246.9
FAM-CC-Di	FAM-GGGG EIAALKQ EIAALKK ENAALKW EIAALKQ G-CONH2	3737.0
CC-Tri	Ac-G EIAAIKQ EIAAIKK EIAAIKW EIAAIKQ G-CONH2	3245.9
FAM-CC Tri	FAM-GGGG EIAAIKQ EIAAIKK EIAAIKW EIAAIKQ G-CONH2	3735.0
CC-Tet	Ac-G ELAAIKQ ELAAIKK ELAAIKW ELAAIKQ G-CONH2	3245.9
FAM-CC-Tet	FAM-GGGG ELAAIKQ ELAAIKK ELAAIKW ELAAIKQ G-CONH2	3735.0
***c*-Register CC**	***cdefgab cdefgab cdefgab cdefgab***	
CC-Pent2	Ac-G EIAQTLK EIAKTLK EIAWTLK EIAQTLK G-CONH2	3365.9
FAM-CC-Pent2	FAM-GGGG EIAQTLK EIAKTLK EIAWTLK EIAQTLK G-CONH2	3855.0
CC-Hex2	Ac-G EIAQSLK EIAKSLK EIAWSLK EIAQSLK G-CONH2	3309.9
FAM-CC-Hex2	FAM-GGGG EIAQSLK EIAKSLK EIAWSLK EIAQSLK G-CONH2	3800.0
CC-Hept	Ac-G EIAQALK EIAKALK EIAWALK EIAQALK G-CONH2	3245.9
FAM-CC-Hept	FAM-GGGG EIAQALK EIAKALK EIAWALK EIAQALK G-CONH2	3735.0

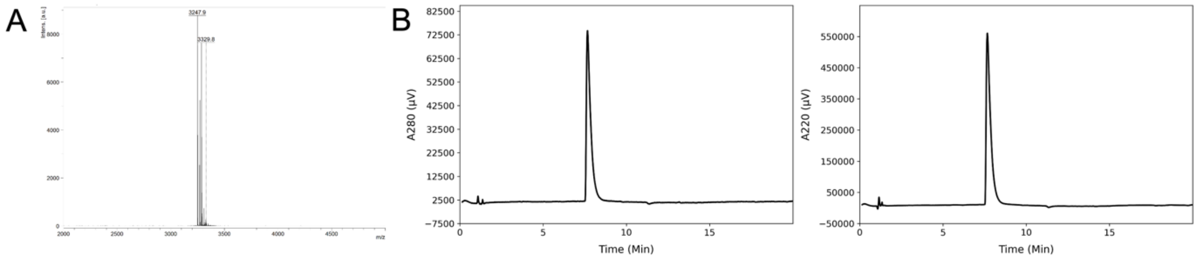

Figure S18. CC-Di MALDI and AHPLC data. (A) MALDI-TOF MS analysis of purified CC-Di. Monoisotopic [M+H] peaks were observed at 3247.9; calculated m/z is 3246.9. (B) Analytical HPLC trace of purified CC-Di. Chromatograms were monitored at 280 (left) and 220 (right) nm wavelengths.

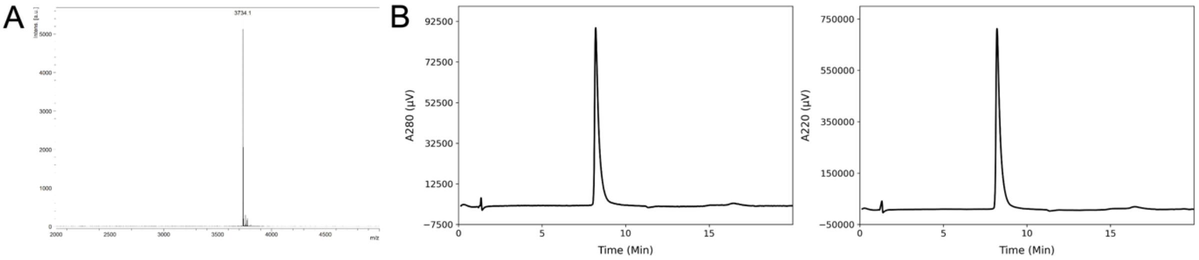

Figure S19. FAM-CC-Di MALDI and AHPLC data. (A) MALDI-TOF MS analysis of purified FAM-CC-Di. Monoisotopic [M+H] peaks were observed at 3734.1; calculated m/z is 3737.0. (B) Analytical HPLC trace of purified FAM-CC-Di. Chromatograms were monitored at 280 (left) and 220 (right) nm wavelengths.

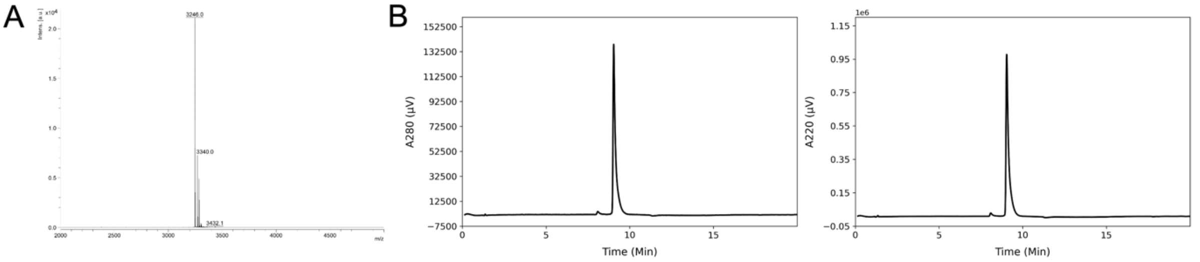

Figure S20. CC-Tri MALDI and AHPLC data. (A) MALDI-TOF MS analysis of purified CC-Tri. Monoisotopic [M+H] peaks were observed at 3246.0; calculated m/z is 3245.9. (B)

Analytical HPLC trace of purified CC-Tri. Chromatograms were monitored at 280 (left) and 220 (right) nm wavelengths

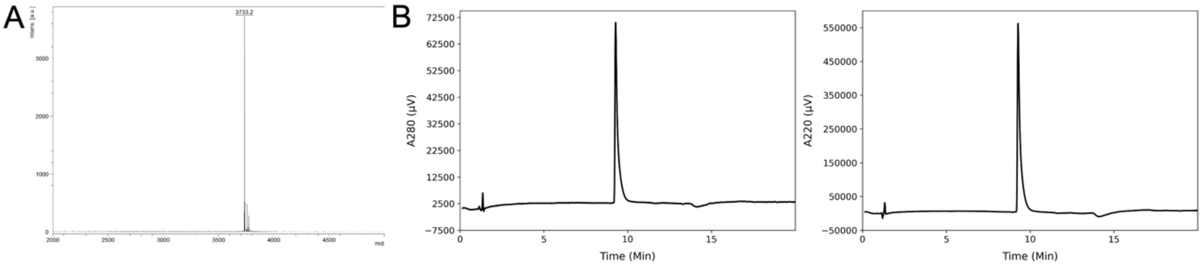

Figure S21. FAM-CC-Tri MALDI and AHPLC data. (A) MALDI-TOF MS analysis of purified FAM-CC-Tri. Monoisotopic [M+H] peaks were observed at 3733.2; calculated m/z is 3735.0. (B) Analytical HPLC trace of purified FAM-CC-Tri. Chromatograms were monitored at 280 (left) and 220 (right) nm wavelengths.

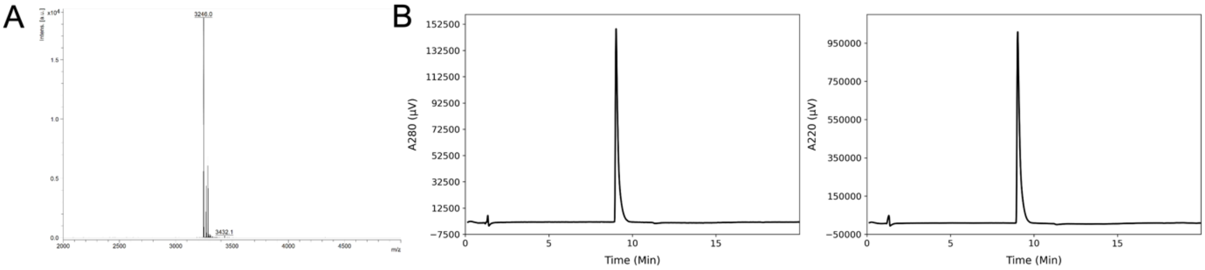

Figure 22. CC-Tet MALDI and AHPLC data. (A) MALDI-TOF MS analysis of purified CC-Tet. Monoisotopic [M+H] peaks were observed at 3246.0; calculated m/z is 3245.9. (B) Analytical HPLC trace of purified CC-Tet. Chromatograms were monitored at 280 (left) and 220 (right) nm wavelengths.

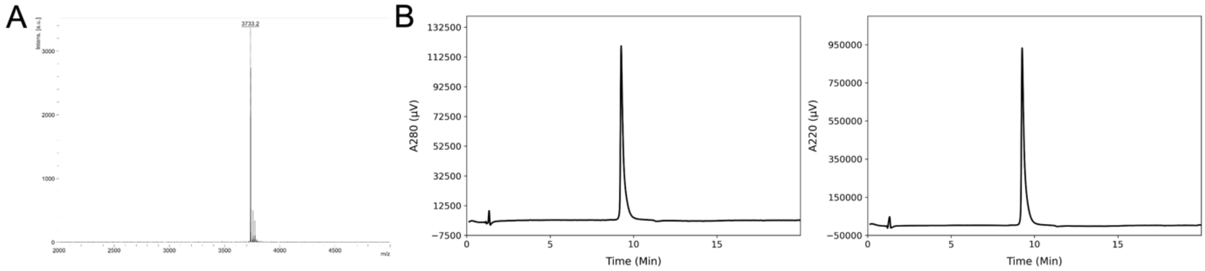

Figure S23. FAM-CC-Tet MALDI and AHPLC data. (A) MALDI-TOF MS analysis of purified FAM-CC-Tet. Monoisotopic [M+H] peaks were observed at 3733.2; calculated m/z is 3735.0. (B) Analytical HPLC trace of purified FAM-CC-Tet. Chromatograms were monitored at 280 (left) and 220 (right) nm wavelengths.

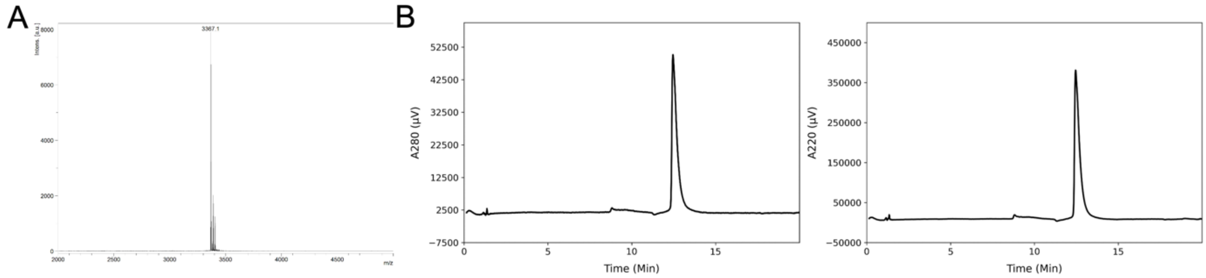

Figure S24. CC-Pent2 MALDI and AHPLC data. (A) MALDI-TOF MS analysis of purified CC-Pent2. Monoisotopic [M+H] peaks were observed at 3367.1; calculated m/z is 3365.9. (B)

Analytical HPLC trace of purified CC-Pent2. Chromatograms were monitored at 280 (left) and 220 (right) nm wavelengths.

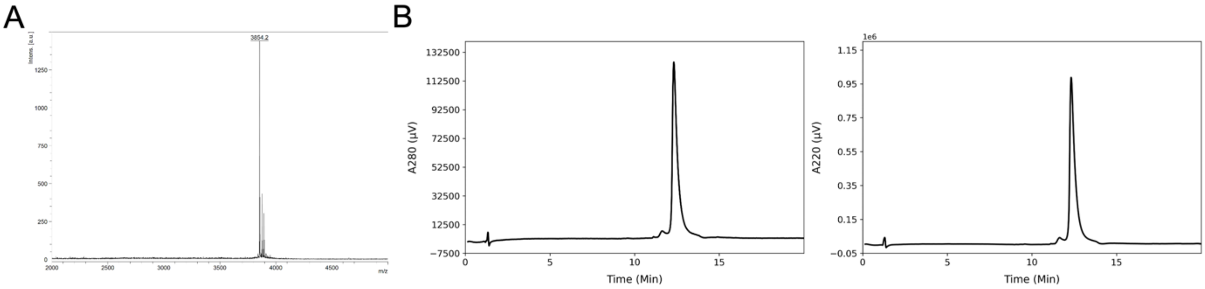

Figure S25. FAM-CC-Pent2 MALDI and AHPLC data. (A) MALDI-TOF MS analysis of purified FAM-CC-Pent2. Monoisotopic [M+H] peaks were observed at 3854.2; calculated m/z is 3855.0. (B) Analytical HPLC trace of purified FAM-CC-Pent2. Chromatograms were monitored at 280 (left) and 220 (right) nm wavelengths.

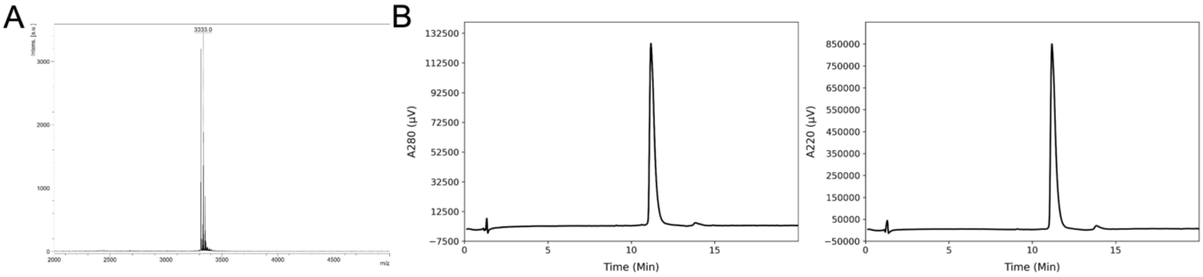

Figure S26. CC-Hex2 MALDI and AHPLC data. (A) MALDI-TOF MS analysis of purified CC-Hex2. Sodium adduct [M+Na] peaks were observed at 3333.0; calculated m/z is 3332.9. (B) Analytical HPLC trace of purified CC-Hex2. Chromatograms were monitored at 280 (left) and 220 (right) nm wavelengths.

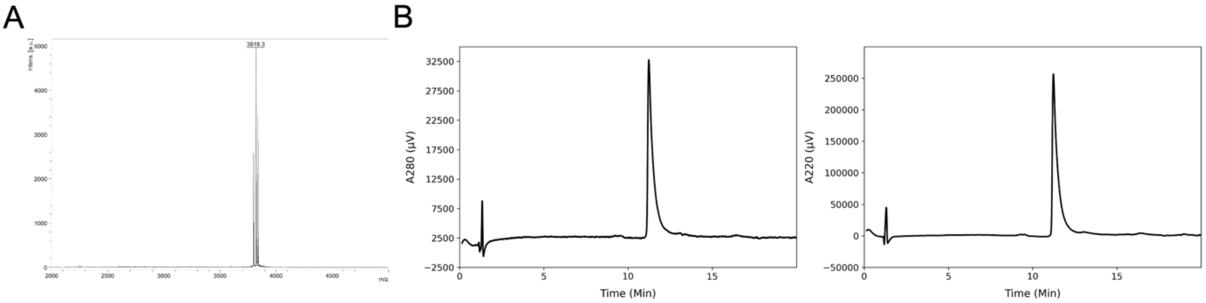

Figure S27. FAM-CC-Hex2 MALDI and AHPLC data. (A) MALDI-TOF MS analysis of purified CC-Hex2. Sodium adduct [M+Na] peaks were observed at 3819.3; calculated m/z is 3823.0. (B) Analytical HPLC trace of purified CC-Hex2. Chromatograms were monitored at 280 (left) and 220 (right) nm wavelengths.

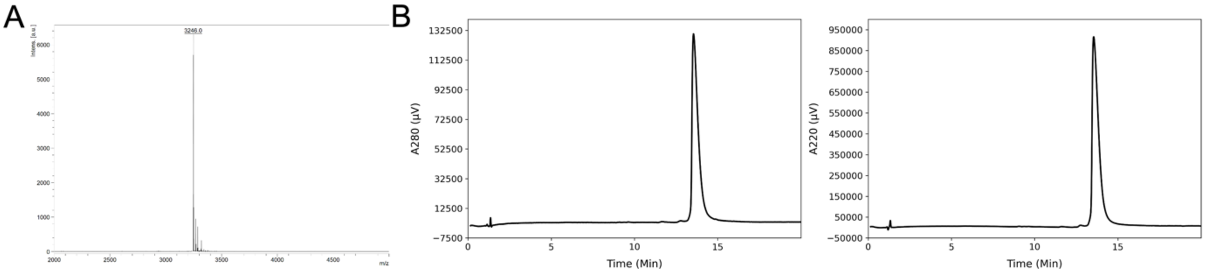

Figure S28. CC-Hept MALDI and AHPLC data. (A) MALDI-TOF MS analysis of purified CC-Hept. Monoisotopic [M+H] peaks were observed at 3246.0; calculated m/z is 3245.9. (B)

Analytical HPLC trace of purified CC-Hept. Chromatograms were monitored at 280 (left) and 220 (right) nm wavelengths.

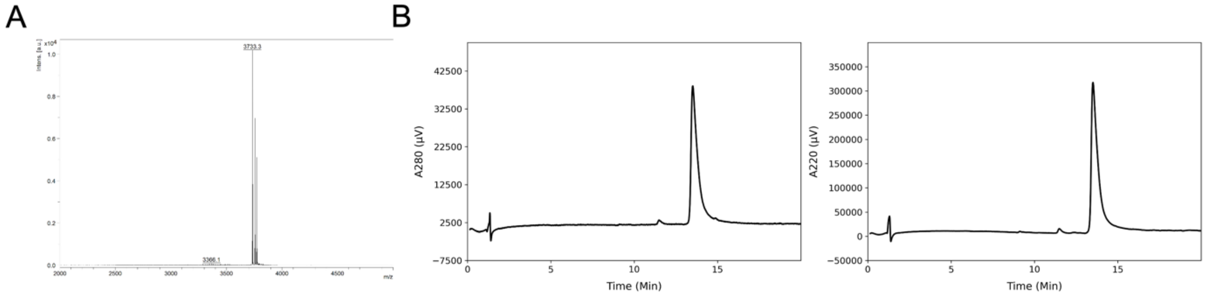

Figure S29. FAM-CC-Hept MALDI and AHPLC data. (A) MALDI-TOF MS analysis of purified FAM-CC-Hept. Monoisotopic [M+H] peaks were observed at 3733.3; calculated m/z is 3735.0. (B) Analytical HPLC trace of purified FAM-CC-Hept. Chromatograms were monitored at 280 (left) and 220 (right) nm wavelengths.

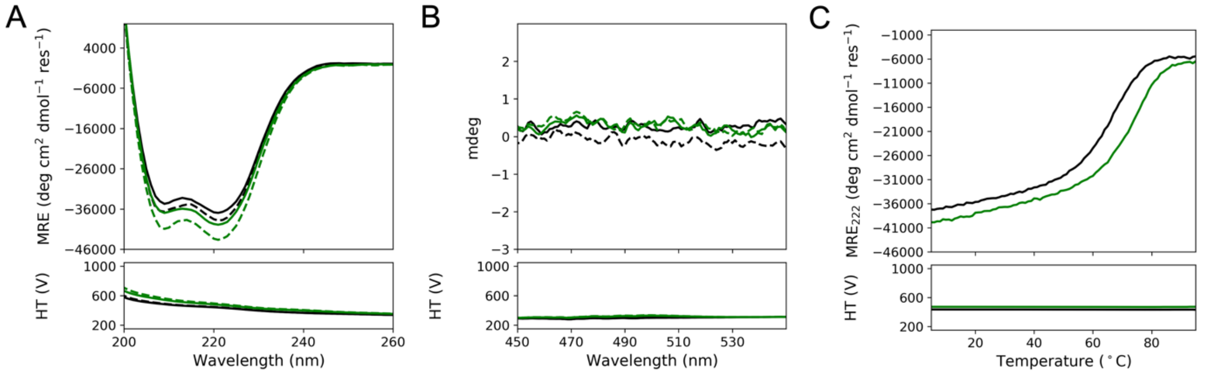

Figure S30. CD Characterization of CC-Di and FAM-CC-Di. CD spectra of 50 μM CC-Di (black) and FAM-CC-Di (green) in PBS at 5 °C are shown in (A) and (B). Samples were analysed prior to melting (solid lines) and post-melting (dotted lines). (A) shows the spectra between 260-200 nm and (B) shows the spectra between 550-450 nm. (C) shows the thermal melt of the two peptides from 5-95 °C. There is not a noticeable difference in the CD characterization of the unlabelled and labelled variants of CC-Di.

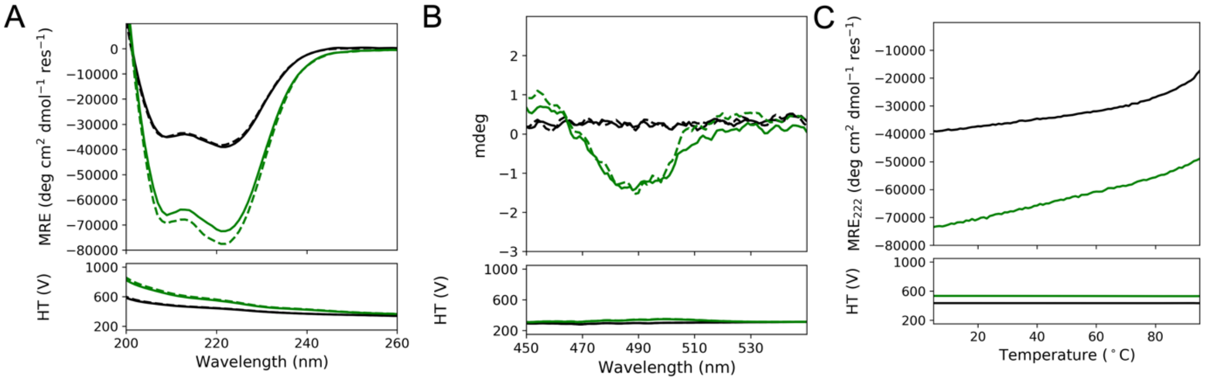

Figure S31. CD Characterization of CC-Tri and FAM-CC-Tri. CD spectra of 50 μM CC-Tri (black) and FAM-CC-Tri (green) in PBS at 5 °C are shown in (A) and (B). Samples were analysed prior to melting (solid lines) and post-melting (dotted lines). (A) shows the spectra between 260-200 nm and (B) shows the spectra between 550-450 nm. (C) shows the thermal melt of the two peptides from 5-9 5°C. The labelled peptide shows an increase in CD signal (A) as well as evidence of the Cotton effect (B). However, the thermal melts are consistent between the samples, indicative that the FAM-labelled peptide has the same thermodynamic properties as the unlabelled peptide.

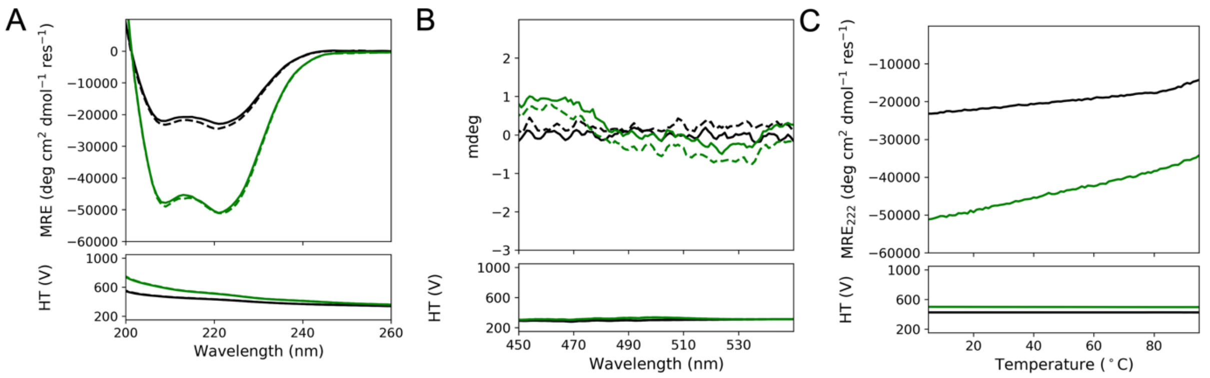

Figure S32. CD Characterization of CC-Tet and FAM-CC-Tet. CD spectra of 50 μM CC-Tet (black) and FAM-CC-Tet (green) in PBS at 5 °C are shown in (A) and (B). Samples were analysed prior to melting (solid lines) and post-melting (dotted lines). (A) shows the spectra between 260-200 nm and (B) shows the spectra between 550-450 nm. (C) shows the thermal melt of the two peptides from 5-95 °C. The labelled peptide shows an increase in CD signal (A). However, the thermal melts are consistent between the samples, indicative that the FAM-labelled peptide has the same thermodynamic properties as the unlabelled peptide.

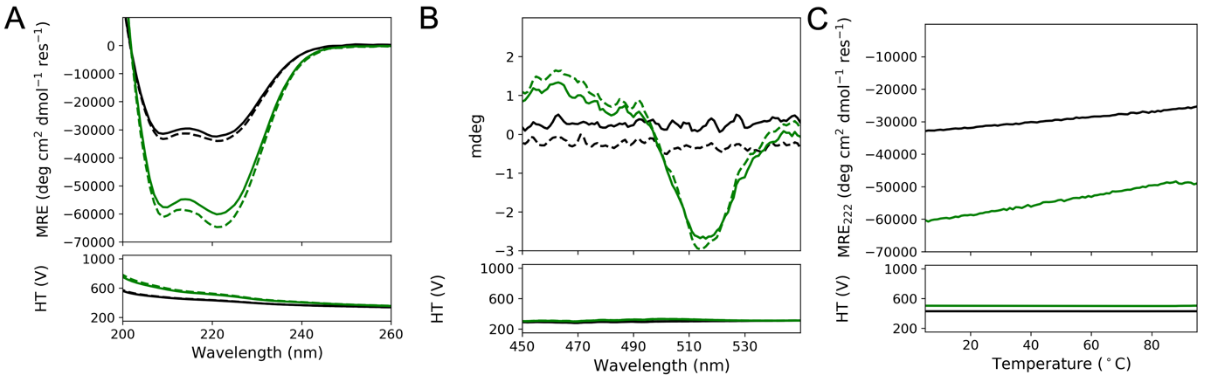

Figure S33. CD Characterization of CC-Pent2 and FAM-CC-Pent2. CD spectra of 50 μM CC-Pent2 (black) and FAM-CC-Pent2 (green) in PBS at 5 °C are shown in (A) and (B). Samples were analysed prior to melting (solid lines) and post-melting (dotted lines). (A) shows the spectra between 260-200 nm and (B) shows the spectra between 550-450 nm. (C) shows the thermal melt of the two peptides from 5-95 °C. The labelled peptide shows an increase in CD signal (A) as well as evidence of Cotton effect (B). However, the thermal melts are consistent between the samples, indicative that the FAM-labelled peptide has the same thermodynamic properties as the unlabelled peptide.

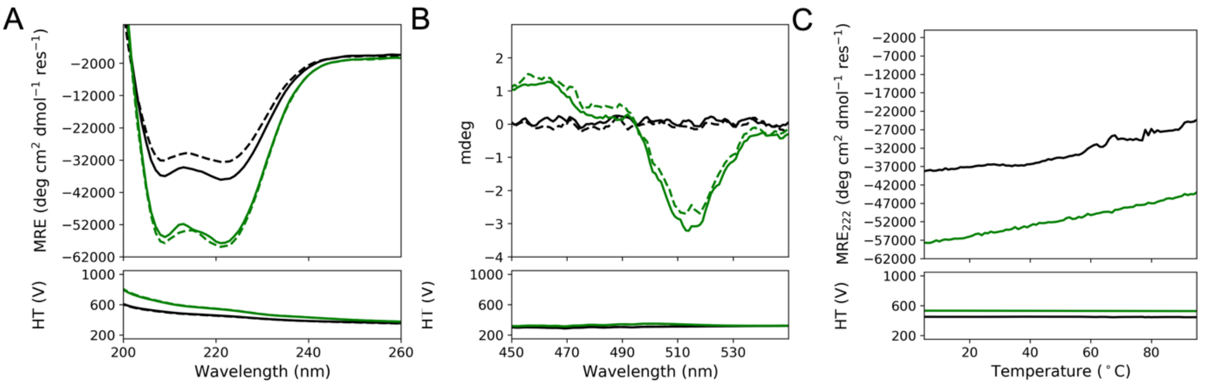

Figure S34. CD Characterization of CC-Hex2 and FAM-CC-Hex2. CD spectra of 50 µM CC-Hex2 (black) and FAM-CC-Hex2 (green) in PBS at 5 °C are shown in (A) and (B). Samples were analysed prior to melting (solid lines) and post-melting (dotted lines). (A) shows the spectra between 260-200 nm and (B) shows the spectra between 550-450 nm. (C) shows the thermal melt of the two peptides from 5-95 °C. The labelled peptide shows an increase in CD signal (A) as well as evidence of Cotton effect (B). However, the thermal melts are consistent between the samples, indicative that the FAM-labelled peptide has the same thermodynamic properties as the unlabelled peptide.

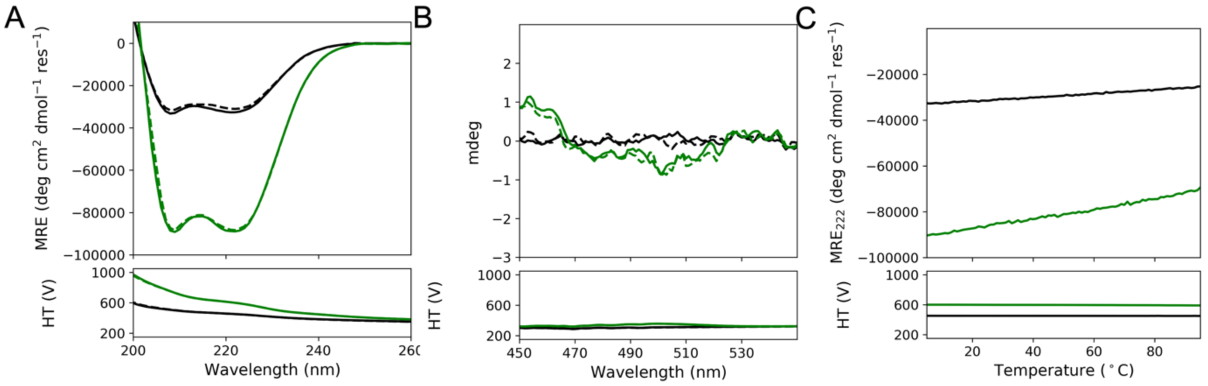

Figure S35. CD Characterization of CC-Hept and FAM-CC-Hept. CD spectra of 50 µM CC-Hept (black) and FAM-CC-Hept (green) in PBS at 5 °C are shown in (A) and (B). Samples were analysed prior to melting (solid lines) and post-melting (dotted lines). (A) shows the spectra between 260-200 nm and (B) shows the spectra between 550-450 nm. (C) shows the thermal melt of the two peptides from 5-95 °C. The labelled peptide shows an increase in CD signal (A). However, the thermal melts are consistent between the samples, indicative that the FAM-labelled peptide has the same thermodynamic properties as the unlabelled peptide.

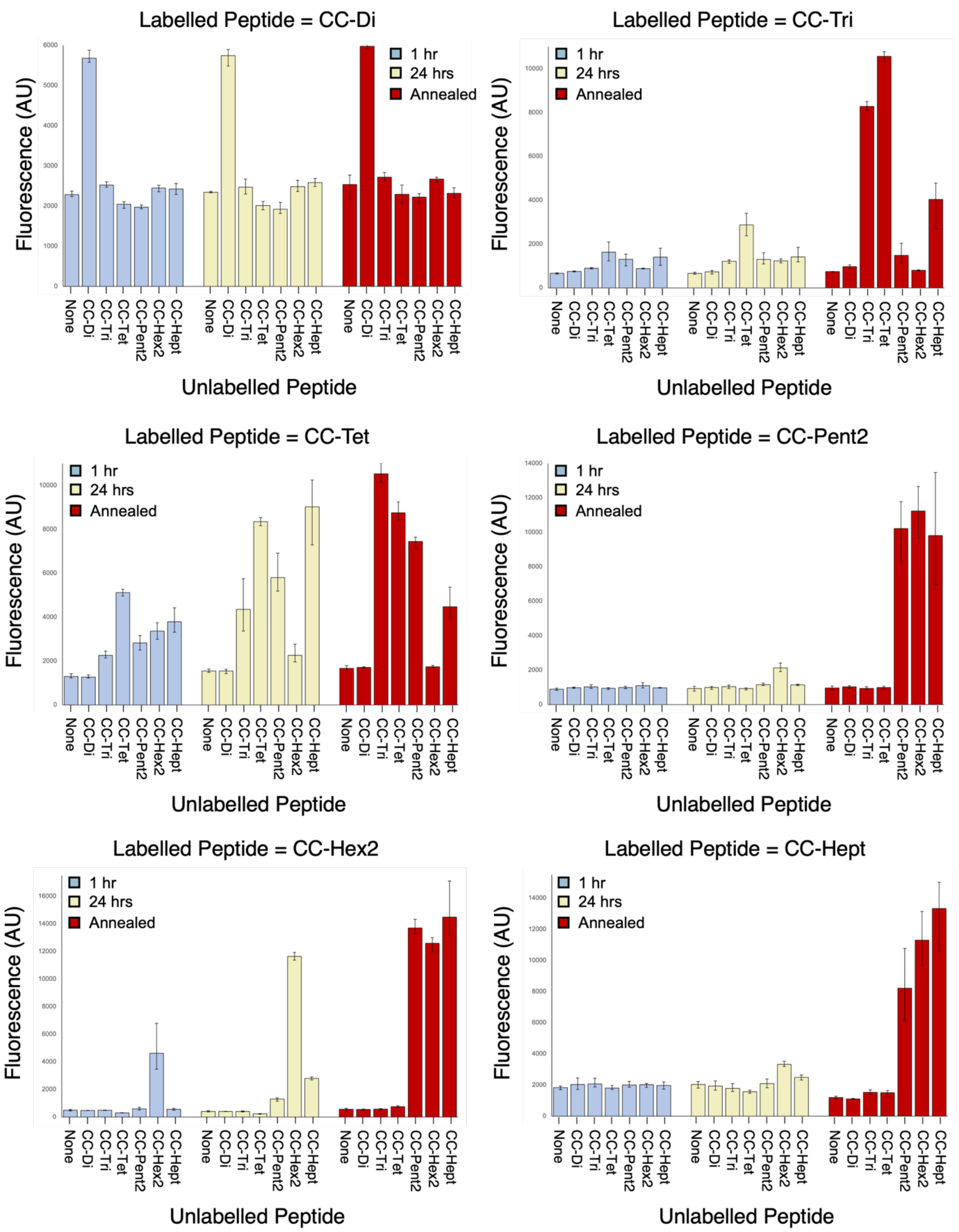

Figure S36. Histogram representation of CC Basis Set fluorescence exchange data. The raw fluorescence measurements of the Basis Set exchange studies are shown. The plotted fluorescence is the averaged value between three measurements. The top and bottom of the error bars are the maximum and minimum fluorescence values observed for each mixture respectively.

Table S6. Raw and Normalised CC Basis Set Fluorescence Data

FAM-labelled Peptide	Unlabelled Peptide	1 hr-raw			1 hr-normalised			Avg	Std Dev
FAM-CC-Di	-	2235	2372	2256	-0.087	-0.048	-0.081	-0.072	0.021
FAM-CC-Di	CC-Di	5577	5880	5582	0.884	0.972	0.885	0.914	0.050
FAM-CC-Di	CC-Tri	2470	2488	2603	-0.087	-0.048	-0.081	-0.072	0.021
FAM-CC-Di	CC-Tet	2106	2082	1944	-0.060	-0.069	-0.122	-0.084	0.033
FAM-CC-Di	CC-Pent2	2028	1959	1933	-0.090	-0.116	-0.126	-0.111	0.019
FAM-CC-Di	CC-Hex2	2354	2520	2471	-0.053	-0.005	-0.019	-0.025	0.025
FAM-CC-Di	CC-Hept	2420	2562	2289	0.060	0.114	0.010	0.061	0.052
FAM-CC-Tri	-	631	685	671	-0.015	-0.007	-0.009	-0.010	0.004
FAM-CC-Tri	CC-Di	758	728	769	0.002	-0.002	0.004	0.001	0.003
FAM-CC-Tri	CC-Tri	867	928	879	0.017	0.025	0.018	0.020	0.004
FAM-CC-Tri	CC-Tet	1231	1574	2094	0.027	0.068	0.131	0.075	0.052
FAM-CC-Tri	CC-Pent2	1008	1379	1536	0.000	0.045	0.064	0.036	0.033
FAM-CC-Tri	CC-Hex2	871	897	861	0.017	0.021	0.016	0.018	0.002
FAM-CC-Tri	CC-Hept	1031	1358	1812	0.003	0.042	0.097	0.047	0.047
FAM-CC-Tet	-	1242	1229	1420	-0.060	-0.062	-0.035	-0.053	0.015
FAM-CC-Tet	CC-Di	1221	1220	1366	-0.063	-0.063	-0.043	-0.057	0.012
FAM-CC-Tet	CC-Tri	2456	2141	2173	0.111	0.067	0.071	0.083	0.024
FAM-CC-Tet	CC-Tet	5114	5269	4964	0.486	0.508	0.465	0.487	0.022
FAM-CC-Tet	CC-Pent2	3162	2818	2500	0.211	0.162	0.117	0.163	0.047
FAM-CC-Tet	CC-Hex2	3742	3367	2991	0.293	0.240	0.187	0.240	0.053
FAM-CC-Tet	CC-Hept	4420	3623	3316	0.388	0.276	0.233	0.299	0.080
FAM-CC-Pent2	-	880	833	958	-0.008	-0.012	0.000	-0.007	0.006
FAM-CC-Pent2	CC-Di	938	1014	992	-0.002	0.006	0.004	0.003	0.004
FAM-CC-Pent2	CC-Tri	940	1150	997	-0.002	0.019	0.004	0.007	0.011
FAM-CC-Pent2	CC-Tet	976	877	985	0.002	-0.008	0.003	-0.001	0.006
FAM-CC-Pent2	CC-Pent2	916	982	1067	-0.004	0.003	0.011	0.003	0.008

FAM-CC-Pent2	CC-Hex2	966	1059	1269	0.001	0.010	0.031	0.014	0.016
FAM-CC-Pent2	CC-Hept	991	961	956	0.003	0.000	0.000	0.001	0.002
FAM-CC-Hex2	-	540	485	442	-0.002	-0.006	-0.010	-0.006	0.004
FAM-CC-Hex2	CC-Di	459	481	470	-0.008	-0.006	-0.007	-0.007	0.001
FAM-CC-Hex2	CC-Tri	503	492	477	-0.005	-0.006	-0.007	-0.006	0.001
FAM-CC-Hex2	CC-Tet	297	317	294	-0.037	-0.036	-0.038	-0.037	0.001
FAM-CC-Hex2	CC-Pent2	510	692	572	-0.019	-0.004	-0.014	-0.013	0.008
FAM-CC-Hex2	CC-Hex2	3468	6785	3593	0.242	0.518	0.252	0.337	0.156
FAM-CC-Hex2	CC-Hept	478	631	577	-0.022	-0.009	-0.014	-0.015	0.007
FAM-CC-Hept	-	1705	1859	1931	0.041	0.054	0.060	0.052	0.010
FAM-CC-Hept	CC-Di	2452	1929	1710	0.103	0.060	0.042	0.068	0.031
FAM-CC-Hept	CC-Tri	2426	1895	1878	0.101	0.057	0.056	0.071	0.026
FAM-CC-Hept	CC-Tet	1969	1721	1706	0.063	0.043	0.042	0.049	0.012
FAM-CC-Hept	CC-Pent2	1891	2231	1864	0.057	0.085	0.055	0.065	0.017
FAM-CC-Hept	CC-Hex2	2103	2099	1855	0.074	0.074	0.054	0.067	0.012
FAM-CC-Hept	CC-Hept	2201	1963	1739	0.082	0.063	0.044	0.063	0.019

**FAM-labelled Peptide**	**Unlabelled Peptide**	**24 hr-raw**			**24 hr-normalised**			**Avg**	**Std Dev**
FAM-CC-Di	-	2366	2328	2333	-0.049	-0.060	-0.059	-0.056	0.006
FAM-CC-Di	CC-Di	5900	5485	5847	0.978	0.857	0.962	0.932	0.066
FAM-CC-Di	CC-Tri	2445	2300	2675	-0.026	-0.069	0.040	-0.018	0.055
FAM-CC-Di	CC-Tet	2116	1907	2022	-0.056	-0.136	-0.092	-0.095	0.040
FAM-CC-Di	CC-Pent2	2093	1818	1859	-0.065	-0.170	-0.155	-0.130	0.057
FAM-CC-Di	CC-Hex2	2441	2363	2644	-0.028	-0.050	0.031	-0.015	0.042
FAM-CC-Di	CC-Hept	2687	2488	2574	0.162	0.086	0.119	0.122	0.038
FAM-CC-Tri	-	646	620	712	-0.013	-0.016	-0.004	-0.011	0.006
FAM-CC-Tri	CC-Di	767	629	794	0.003	-0.015	0.007	-0.001	0.012
FAM-CC-Tri	CC-Tri	1279	1118	1233	0.071	0.050	0.065	0.062	0.011

FAM-CC-Tri	CC-Tet	2849	3399	2378	0.222	0.288	0.165	0.225	0.062
FAM-CC-Tri	CC-Pent2	1194	1606	1095	0.022	0.072	0.010	0.035	0.033
FAM-CC-Tri	CC-Hex2	1232	1144	1329	0.065	0.054	0.078	0.066	0.012
FAM-CC-Tri	CC-Hept	1201	1852	1187	0.023	0.102	0.021	0.049	0.046
FAM-CC-Tet	-	1535	1483	1630	-0.019	-0.026	-0.006	-0.017	0.011
FAM-CC-Tet	CC-Di	1627	1425	1579	-0.006	-0.035	-0.013	-0.018	0.015
FAM-CC-Tet	CC-Tri	5748	3365	3939	0.576	0.239	0.320	0.379	0.176
FAM-CC-Tet	CC-Tet	8363	8161	8530	0.945	0.917	0.969	0.944	0.026
FAM-CC-Tet	CC-Pent2	6915	5299	5179	0.741	0.513	0.496	0.583	0.137
FAM-CC-Tet	CC-Hex2	2768	1957	2052	0.155	0.041	0.054	0.083	0.063
FAM-CC-Tet	CC-Hept	10242	7291	9528	1.210	0.794	1.110	1.038	0.217
FAM-CC-Pent2	-	939	782	1058	-0.002	-0.018	0.000	-0.006	0.010
FAM-CC-Pent2	CC-Di	886	1042	1044	-0.007	0.009	0.004	0.002	0.008
FAM-CC-Pent2	CC-Tri	931	1137	1040	-0.003	0.018	0.004	0.007	0.011
FAM-CC-Pent2	CC-Tet	958	847	968	0.000	-0.011	0.003	-0.003	0.007
FAM-CC-Pent2	CC-Pent2	1116	1113	1248	0.016	0.016	0.029	0.020	0.008
FAM-CC-Pent2	CC-Hex2	2082	1912	2409	0.113	0.096	0.146	0.119	0.025
FAM-CC-Pent2	CC-Hept	1185	1099	1157	0.023	0.014	0.020	0.019	0.004
FAM-CC-Hex2	-	460	391	373	-0.008	-0.014	-0.015	-0.013	0.004
FAM-CC-Hex2	CC-Di	416	396	415	-0.012	-0.014	-0.012	-0.012	0.001
FAM-CC-Hex2	CC-Tri	441	372	397	-0.010	-0.016	-0.013	-0.013	0.003
FAM-CC-Hex2	CC-Tet	197	239	251	-0.046	-0.042	-0.038	-0.042	0.004
FAM-CC-Hex2	CC-Pent2	1381	1371	1148	0.054	0.053	0.034	0.047	0.011
FAM-CC-Hex2	CC-Hex2	11610	11929	11367	0.919	0.945	0.899	0.921	0.023
FAM-CC-Hex2	CC-Hept	2681	2910	2798	0.164	0.183	0.174	0.174	0.010
FAM-CC-Hept	-	2225	1805	2066	0.084	0.050	0.071	0.068	0.017
FAM-CC-Hept	CC-Di	2264	1855	1671	0.088	0.054	0.039	0.060	0.025
FAM-CC-Hept	CC-Tri	2095	1656	1585	0.074	0.037	0.032	0.048	0.023
FAM-CC-Hept	CC-Tet	1675	1571	1474	0.039	0.030	0.022	0.031	0.008

FAM-CC-Hept	CC-Pent2	1820	2386	2075	0.051	0.098	0.072	0.074	0.023
FAM-CC-Hept	CC-Hex2	3257	3517	3190	0.169	0.191	0.164	0.175	0.014
FAM-CC-Hept	CC-Hept	2521	2637	2324	0.109	0.118	0.093	0.107	0.013

**FAM-labelled Peptide**	**Unlabelled Peptide**	**annealed-raw**			**annealed-normalised**			**Avg**	**Std Dev**
FAM-CC-Di	-	2170	2669	2769	-0.106	0.039	0.068	0.000	0.093
FAM-CC-Di	CC-Di	5943	6068	5920	0.990	1.026	0.983	1.000	0.023
FAM-CC-Di	CC-Tri	2837	2642	2676	0.087	0.031	0.041	0.053	0.030
FAM-CC-Di	CC-Tet	2297	2524	2064	0.013	0.100	-0.076	0.012	0.088
FAM-CC-Di	CC-Pent2	2272	2311	2084	0.003	0.018	-0.069	-0.016	0.046
FAM-CC-Di	CC-Hex2	2720	2703	2593	0.053	0.049	0.017	0.040	0.020
FAM-CC-Di	CC-Hept	2287	2455	2208	0.009	0.073	-0.021	0.020	0.048
FAM-CC-Tri	-	751	717	754	0.001	-0.003	0.002	0.000	0.003
FAM-CC-Tri	CC-Di	1046	888	975	0.041	0.020	0.031	0.030	0.010
FAM-CC-Tri	CC-Tri	8243	8496	8093	0.995	1.029	0.976	1.000	0.027
FAM-CC-Tri	CC-Tet	10332	10578	10772	1.125	1.155	1.178	1.153	0.027
FAM-CC-Tri	CC-Pent2	1133	1286	2033	0.015	0.033	0.124	0.057	0.058
FAM-CC-Tri	CC-Hex2	781	794	822	0.005	0.007	0.011	0.008	0.003
FAM-CC-Tri	CC-Hept	2700	4778	4634	0.204	0.455	0.438	0.366	0.140
FAM-CC-Tet	-	1599	1618	1791	-0.010	-0.007	0.017	0.000	0.015
FAM-CC-Tet	CC-Di	1723	1671	1740	0.008	0.000	0.010	0.006	0.005
FAM-CC-Tet	CC-Tri	11003	10434	10148	1.318	1.238	1.197	1.251	0.061
FAM-CC-Tet	CC-Tet	8405	9248	8601	0.951	1.070	0.979	1.000	0.062
FAM-CC-Tet	CC-Pent2	7101	7645	7586	0.767	0.844	0.835	0.815	0.042
FAM-CC-Tet	CC-Hex2	1708	1697	1800	0.005	0.004	0.018	0.009	0.008
FAM-CC-Tet	CC-Hept	5367	4066	3985	0.522	0.338	0.327	0.396	0.110
FAM-CC-Pent2	-	972	821	1076	0.002	-0.014	0.012	0.000	0.013
FAM-CC-Pent2	CC-Di	947	1045	1093	-0.001	0.009	0.014	0.007	0.007

FAM-CC-Pent2	CC-Tri	915	1039	852	-0.004	0.008	-0.010	-0.002	0.010
FAM-CC-Pent2	CC-Tet	1000	913	1063	0.004	-0.004	0.011	0.004	0.008
FAM-CC-Pent2	CC-Pent2	11785	10334	10570	1.089	0.943	0.967	1.000	0.078
FAM-CC-Pent2	CC-Hex2	12662	9654	11397	1.178	0.875	1.050	1.034	0.152
FAM-CC-Pent2	CC-Hept	13474	10814	8992	1.259	0.992	0.808	1.020	0.227
FAM-CC-Hex2	-	632	499	546	0.006	-0.005	-0.001	0.000	0.006
FAM-CC-Hex2	CC-Di	567	508	572	0.001	-0.004	0.001	-0.001	0.003
FAM-CC-Hex2	CC-Tri	615	519	592	0.005	-0.003	0.003	0.001	0.004
FAM-CC-Hex2	CC-Tet	698	822	759	-0.004	0.007	0.002	0.002	0.005
FAM-CC-Hex2	CC-Pent2	14317	13273	13498	1.148	1.059	1.078	1.095	0.046
FAM-CC-Hex2	CC-Hex2	13004	11976	12783	1.035	0.949	1.016	1.000	0.045
FAM-CC-Hex2	CC-Hept	17102	13215	13161	1.383	1.055	1.050	1.163	0.191
FAM-CC-Hept	-	2225	1805	2066	0.006	-0.007	0.001	0.000	0.007
FAM-CC-Hept	CC-Di	2264	1855	1671	-0.005	-0.007	-0.011	-0.008	0.003
FAM-CC-Hept	CC-Tri	2095	1656	1585	0.018	0.024	0.040	0.027	0.011
FAM-CC-Hept	CC-Tet	1675	1571	1474	0.006	0.034	0.037	0.026	0.017
FAM-CC-Hept	CC-Pent2	1820	2386	2075	0.789	0.408	0.537	0.578	0.194
FAM-CC-Hept	CC-Hex2	3257	3517	3190	0.984	0.701	0.812	0.832	0.143
FAM-CC-Hept	CC-Hept	2521	2637	2324	1.088	0.773	1.139	1.000	0.198

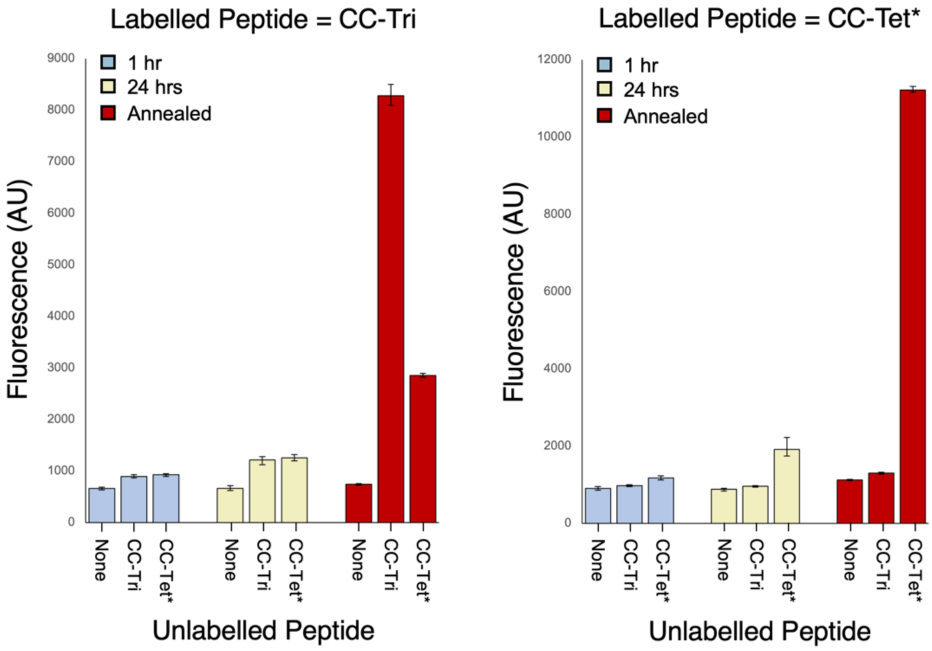

Figure S37. Histogram representation of CC-Tri and CC-Tet* fluorescence exchange data. The raw fluorescence measurements of the CC-Tri and CC-Tet* exchange studies are shown. The plotted fluorescence is the averaged value between three measurements. The top and bottom of the error bars are the maximum and minimum fluorescence values observed for each mixture respectively.

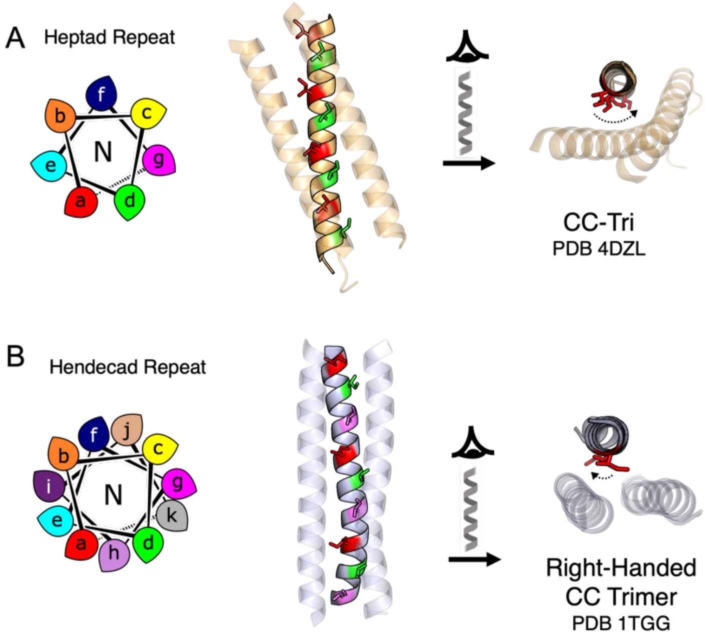

Figure S38. A comparison of the canonical CC heptad repeat and noncanonical hendecad repeat. A) Helical wheel representation of a heptad repeat where positions ***a*** and ***d*** (red and green, respectively) contribute to the coiled-coil interface. This is illustrated by CC-Tri,[1] which forms a left-handed coiled coil. (B) A similar helical wheel for a hendecad repeat where positions ***a***, ***d*** and ***h*** (coloured red, green, and lilac, respectively) define the coiled-coil interface. This is illustrated by the PDB entry 1tgg, which forms a straightened coiled-coil trimer.[21]

Table S7. Orthogonal Set Sequences

***g*-Register CC**	***gabcdef gabcdef gabcdef gabcdef***	**Monoistopic Mass**
CC-Di	Ac-G EIAALKQ EIAALKK ENAALKW EIAALKQ G-CONH2	3246.9
FAM-CC-Di	FAM-GGGG EIAALKQ EIAALKK ENAALKW EIAALKQ G-CONH2	3737.0
CC-Tri	Ac-G EIAAIKQ EIAAIKK EIAAIKW EIAAIKQ G-CONH2	3245.9
FAM-CC Tri	FAM-GGGG EIAAIKQ EIAAIKK EIAAIKW EIAAIKQ G-CONH2	3735.0
***c*-Register CC**	***cdefgab cdefgab cdefgab cdefgab***	
CC-Tet*	Ac-G EIQKQLK EIQKQLK EIQWQLK EIQKQLK G-CONH2	3703.1
FAM-CC-Tet*	FAM-GGGG EIQKQLK EIQKQLK EIQWQLK EIQKQLK G-CONH2	4192.3
CC-Hex2	Ac-G EIAQSLK EIAKSLK EIAWSLK EIAQSLK G-CONH2	3309.9
FAM-CC-Hex2	FAM-GGGG EIAQSLK EIAKSLK EIAWSLK EIAQSLK G-CONH2	3800.0
	cdefgab cdefgab cdefghijkab cdefgab	
CC-Pent2-hen3	Ac-G EIAQALK EIAKALK EIAWALAAALK EIAQALK G-CONH2	3692.1
FAM-CC-Pent2-hen3	FAM-GGGG EIAQALK EIAKALK EIAWALAAALK EIAQALK G-CONH2	4182.3
	cdefgab cdefghijkab cdefgab cdefgab	
CC-Hept-IV-hen2	Ac-G EVAQAIK EVAKAVAAAIK EVAWAIK EVAQAIK G-CONH2	3502.0
FAM-CC-Hept-IV-hen2	FAM-GGGG EVAQAIK EVAKAVAAAIK EVAWAIK EVAQAIK G-CONH2	3992.1

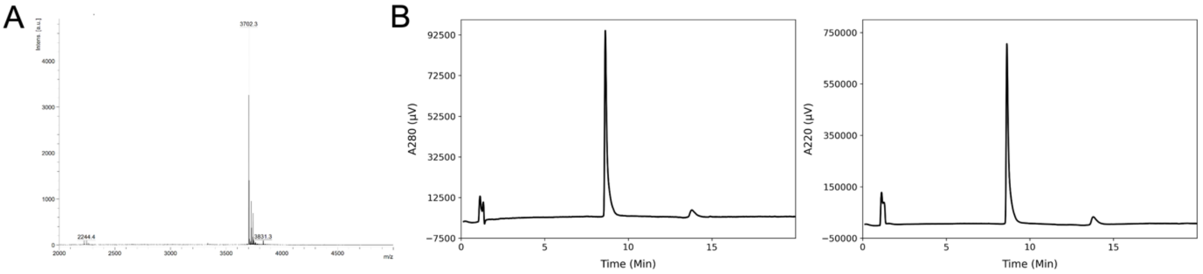

Figure S39. MALDI and AHPLC of CC-Tet*. (A) MALDI-TOF MS analysis of purified CC-Tet*. Monoisotopic [M+H] peaks were observed at 3702.3; calculated m/z is 3703.1. (B) Analytical HPLC trace of purified CC-Tet*. Chromatograms were monitored at 280 (left) and 220 (right) nm wavelengths.

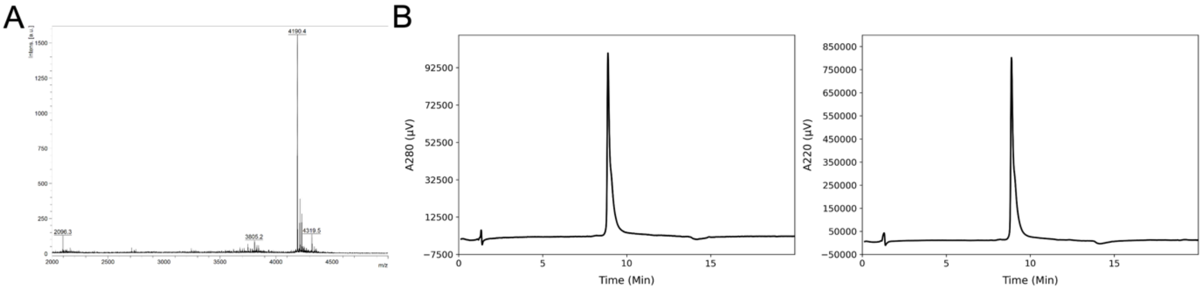

Figure S40. MALDI and AHPLC of FAM-CC-Tet*. (A) MALDI-TOF MS analysis of purified FAM-CC-Tet*. Monoisotopic [M+H] peaks were observed at 4190.4; calculated m/z is 4192.3. (B) Analytical HPLC trace of purified FAM-CC-Tet*. Chromatograms were monitored at 280 (left) and 220 (right) nm wavelengths.

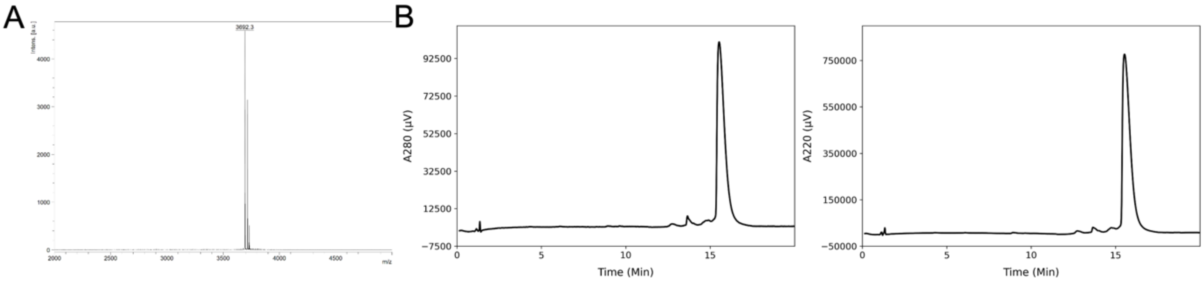

Figure S41. MALDI and AHPLC of CC-Pent2-hen3. (A) MALDI-TOF MS analysis of purified CC-Pent2-hen3. Monoisotopic [M+H] peaks were observed at 3692.3; calculated m/z is

3692.1. (B) Analytical HPLC trace of purified CC-Pent2-hen3. Chromatograms were monitored at 280 (left) and 220 (right) nm wavelengths.

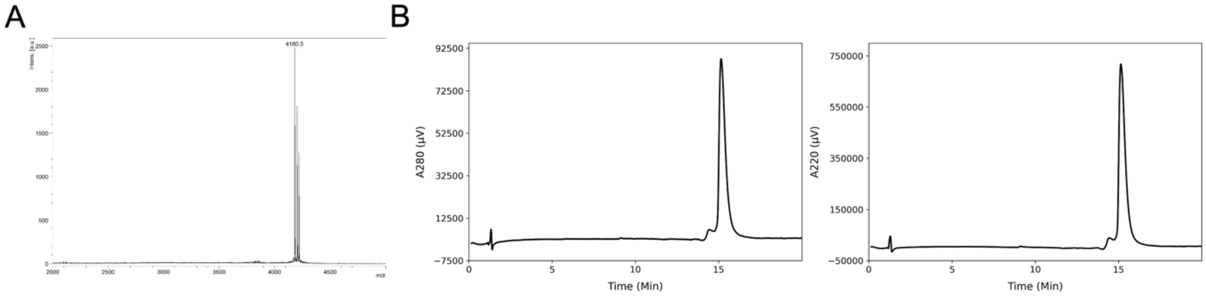

Figure S42. MALDI and AHPLC of FAM-CC-Pent2-hen3. (A) MALDI-TOF MS analysis of purified FAM-CC-Pent2-hen3. Monoisotopic [M+H] peaks were observed at 4180.5; calculated m/z is 4182.3. (B) Analytical HPLC trace of purified FAM-CC-Pent2-hen3. Chromatograms were monitored at 280 (left) and 220 (right) nm wavelengths.

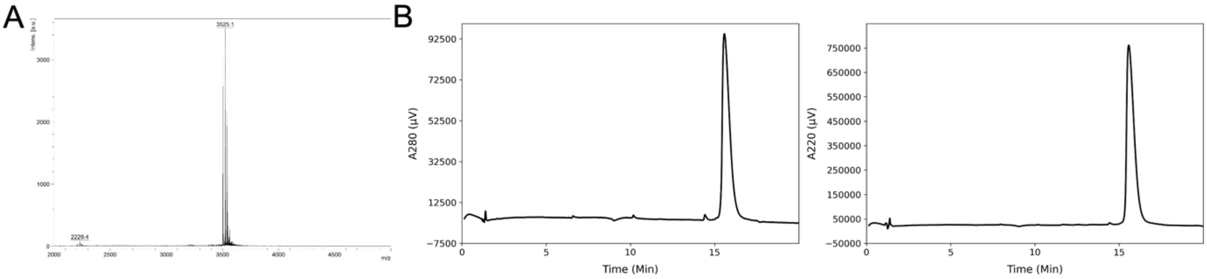

Figure S43. MALDI and AHPLC of CC-Hept-IV-hen2. (A) MALDI-TOF MS analysis of purified CC-Hept-IV-hen2. Sodium adduct [M+Na] peaks were observed at 3525.1; calculated m/z is 3525.0. (B) Analytical HPLC trace of purified CC-Hept-IV-hen2. Chromatograms were monitored at 280 (left) and 220 (right) nm wavelengths.

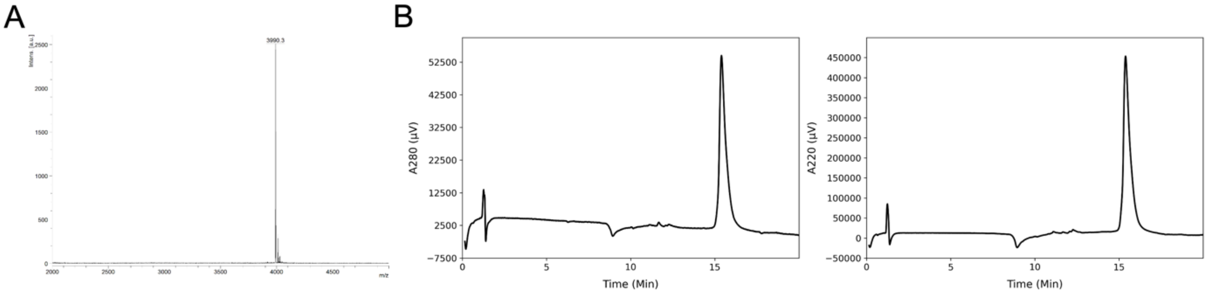

Figure S44. MALDI and AHPLC of FAM-CC-Hept-IV-hen2. (A) MALDI-TOF MS analysis of purified CC-Hept-IV-hen2. Sodium adduct [M+Na] peaks were observed at 3990.3; calculated m/z is 3992.1. (B) Analytical HPLC trace of purified CC-Hept-IV-hen2. Chromatograms were monitored at 280 (left) and 220 (right) nm wavelengths.

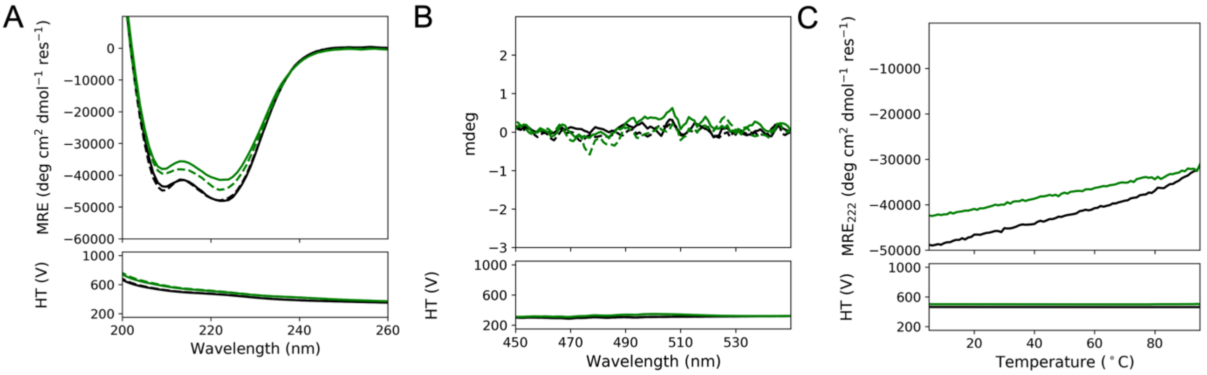

Figure S45. CD Characterization of CC-Tet* and FAM-CC-Tet*. CD spectra of 50 µM CC-Tet* (black) and FAM-CC-Tet* (green) in PBS at 5 °C are shown in (A) and (B). Samples were analysed prior to melting (solid lines) and post-melting (dotted lines). (A) shows the spectra between 260-200 nm and (B) shows the spectra between 550-450 nm. (C) shows the thermal melt of the two peptides from 5-95 °C. There is not a noticeable difference in the CD characterization of the unlabelled and labelled variants of CC-Tet*.

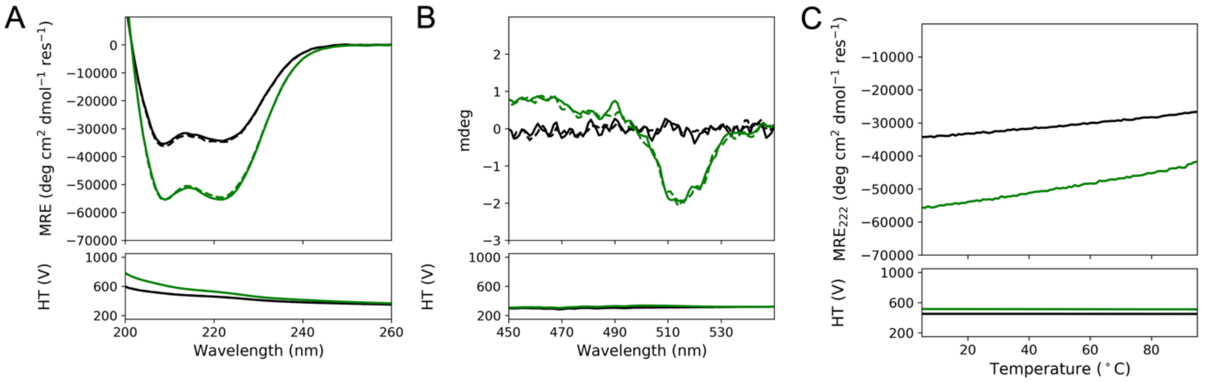

Figure S46. CD Characterization of CC-Pent2-hen3 and FAM- Pent2-hen3. CD spectra of 50 µM CC-Pent2-hen3 (black) and FAM-CC-Pent2-hen3 (green) in PBS at 5 °C are shown in (A) and (B). Samples were analysed prior to melting (solid lines) and post-melting (dotted lines). (A) shows the spectra between 260-200 nm and (B) shows the spectra between 550-450 nm. (C) shows the thermal melt of the two peptides from 5-95 °C. The labelled peptide shows an increase in CD signal (A) as well as evidence of the Cotton effect (B). However, the thermal melts are consistent between the samples, indicative that the FAM-labelled peptide has the same thermodynamic properties as the unlabelled peptide.

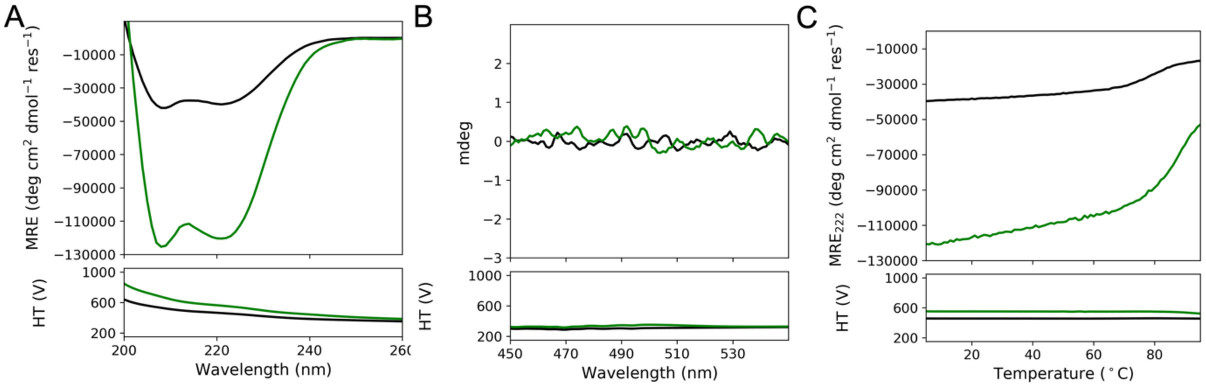

Figure S47. CD Characterization of CC-Hept-IV-hen2 and FAM-Hept-IV-hen2. CD spectra of 50 µM CC-Hept-IV-hen2 (black) and 25 µM FAM-CC-Hept-IV-hen2 (green) in PBS at 5 °C are shown in (A) and (B). Samples were analysed prior to melting (solid lines) and post-melting

(dotted lines). (A) shows the spectra between 260-200 nm and (B) shows the spectra between 550-450 nm. (C) shows the thermal melt of the two peptides from 5-95 °C. The labelled peptide shows an increase in CD signal (A). However, the thermal melts are consistent between the samples, indicative that the FAM-labelled peptide has the same thermodynamic properties as the unlabelled peptide.

Table S8. Table 1 of CC-Hex2-hen2 with Ala at ***h*****,** CC-Hept-hen2 with Ala at ***h**,* and CC-Hept-IV-hen2 structures.

	**CC-Hex2-hen2, Ala at *h* (PDB 9gf2)**	**CC-Hept-hen2, Ala at *h* (PDB 9gf3)**	**CC-Hept-IV-hen2 (PDB 9gf4)**
Wavelength (Å)	0.9795	0.9795	0.9795
Resolution Range	48.56 - 2.10 (2.31 - 2.1)	47.51-1.65 (1.67-1.65)	19.67-1.49 (1.52-1.49)
Space Group	P 2 21 21	P 21 21 21	P 2 21 21
Unit Cell	48.563 58.157 59.258 90 90 90	60.267 61.754 154.441 90 90 90	38.546 47.919 153.02 90 90 90
Unique Reflections	10246 (2510)	70117 (2831)	47044 (2595)
Completeness (%)	99.59 (99.84)	99.83 (100.00)	99.57 (95.65)
Wilson B-factor	33.40	21.79	20.26
Reflections used in refinement	10246 (2510)	70117 (2831)	47044 (2595)
Reflections used for R-free	532(114)	3455 (135)	2431 (148)
R-work	0.2252 (0.1904)	0.1939 (0.3003)	0.1715 (0.2311)
R-free	0.2220 (0.1944)	0.2148 (0.3255)	0.1972 (0.2912)
Number of non-hydrogen atoms	1426	4046	1999
macromolecules	1372	3474	1716
ligands	16	173	90
solvent	38	399	193
Protein Residues	190	476	238
RMS(bonds)	0.022	0.059	0.064
RMS(angles)	2.52	1.49	1.27
Ramachandran favoured(%)	96.63	100.00	100.00
Ramachandran allowed(%)	1.69	0.00	0.00
Ramachandran outliers(%)	1.69	0.00	0.00
Rotamer outliers(%)	7.81	0.00	0.00
Clashscore	7.22	2.88	1.33
Average B-factor	52.62	29.07	25.71
macromolecules	53.04	26.47	22.82
ligands	43.57	55.53	51.11
solvent	41.15	40.22	39.54

Table S9. Oligomeric states of CC-Pent2-hen3 and CC-Hept-IV-hen2 measured by AUC (SV and SE) and in crystal structures.

Peptide	Oligomeric state based on SV data	Oligomeric state based on SV data	Oligomeric state based on crystallography data
CC-Pent2-hen3	5.3	4.9	ND
CC-Hept-IV-hen2	6.1	6.0	7

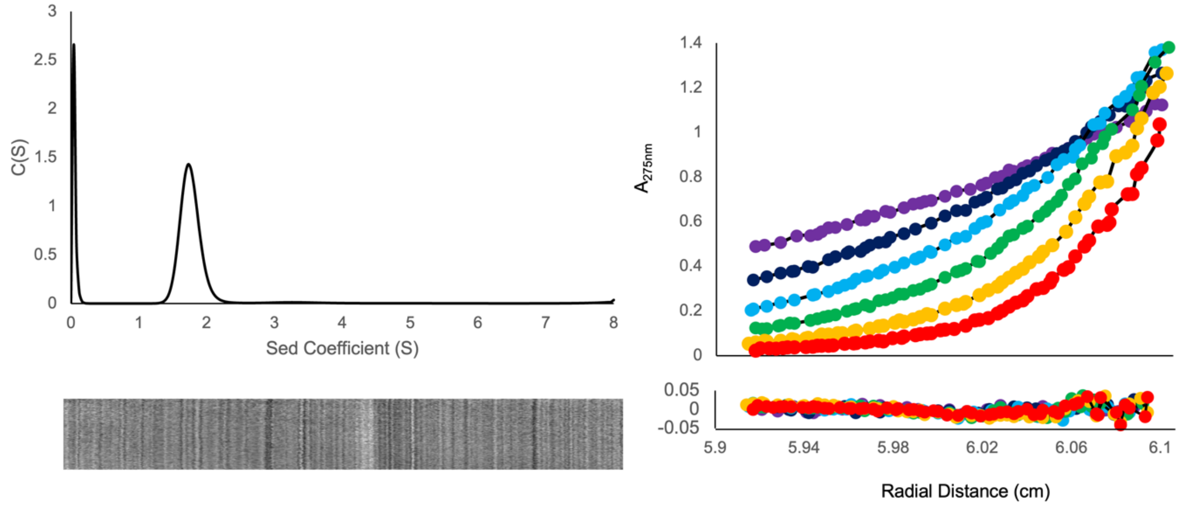

Figure S48. AUC data for CC-Pent2-hen3. Left shows the sedimentation velocity (SV) data acquired for CC-Pent2-hen3. The molecular weight calculated from the data is 19558 Da = 5.3 x the monomeric mass of CC-Pent2-hen3 (3693.4 Da) (f/f_0 = 1.225, s = 1.757, sw(20,w) = 1.798 S). Right shows the sedimentation equilibrium data acquired for CC-Pent2-hen3. The molecular weight derived from the data is 18098 Da = 4.9 x the monomeric mass of CC-Pent2-hen3 (χ^2 = 4.98).

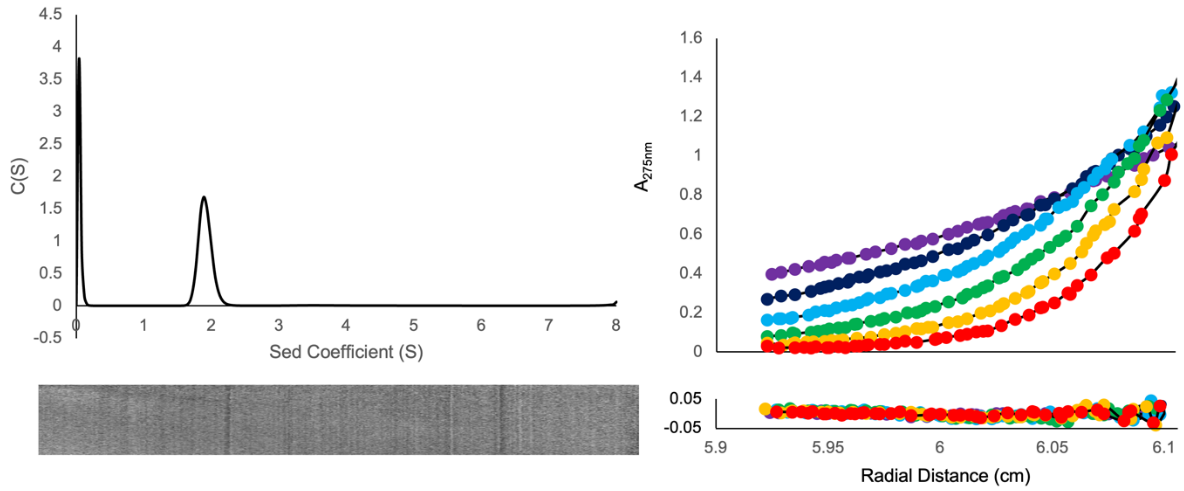

Figure S49. AUC data for CC-Hept-IV-hen2. Left shows the sedimentation velocity (SV) data acquired for CC-Hept-IV-hen2. The molecular weight calculated from the data is 21185 Da = 6.1 x the monomeric mass of CC-Hept-IV-hen2 (3503.1 Da) (f/f_0 = 1.204, s = 1.909, sw(20,w) = 1.953 S). Right shows the sedimentation equilibrium data acquired for CC-Hept-IV-hen2. The molecular weight derived from the data is 20904 Da = 6.0 x the monomeric mass of CC-Hept-IV-hen2 (χ^2 = 3.94).

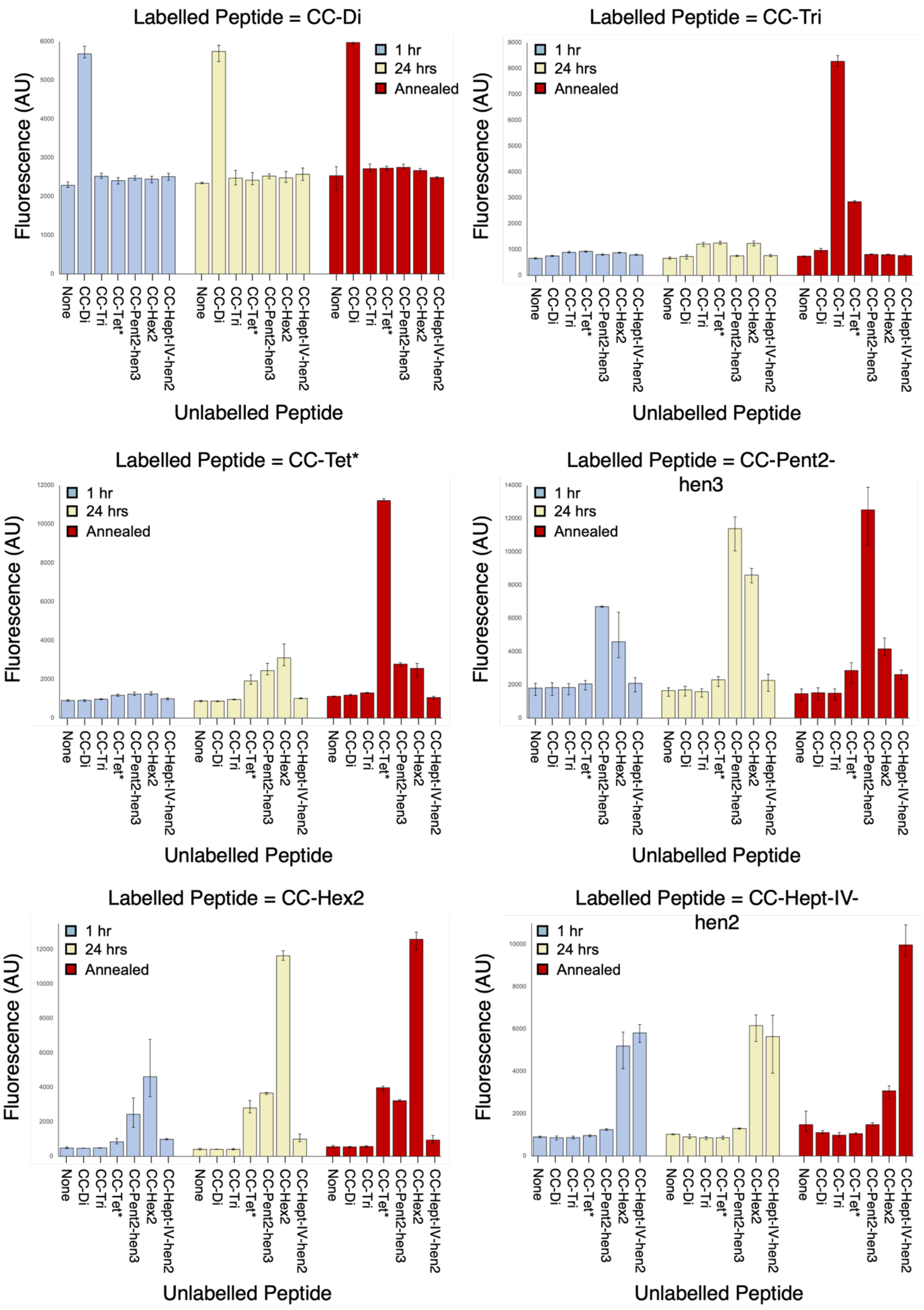

Figure S50. Histogram representation of Orthogonal CC Set fluorescence exchange data. The raw fluorescence measurements of the Orthogonal CC Set exchange studies are shown. The plotted fluorescence is the averaged value between three measurements. The

top and bottom of the error bars are the maximum and minimum fluorescence values observed for each mixture respectively.

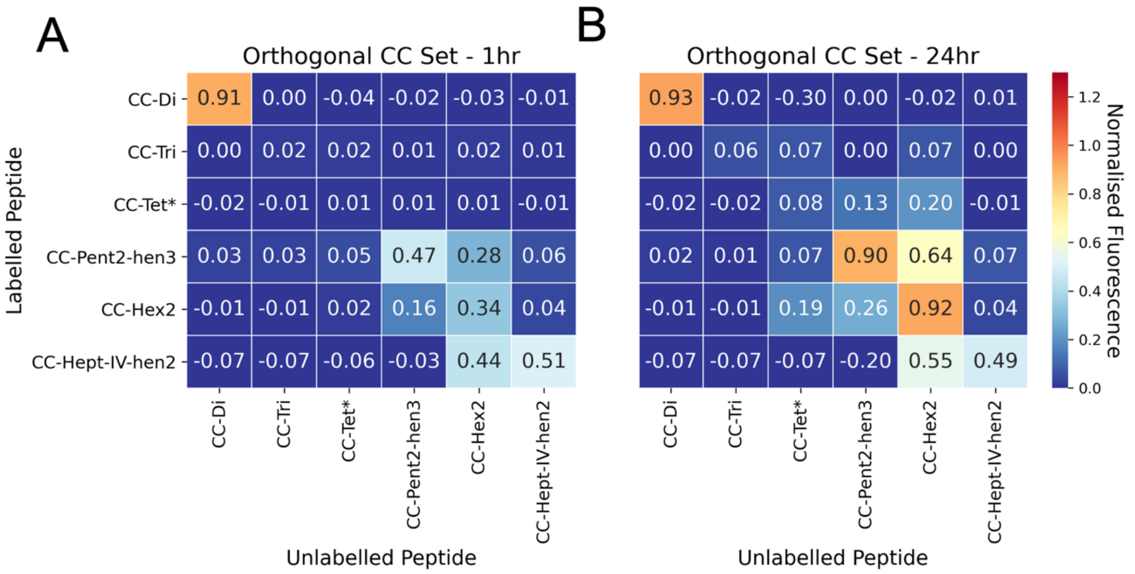

Figure S51. Heat map representation of Orthogonal CC Set fluorescence exchange data acquired at 1 hr and 24 hrs. The normalised values of the Orthogonal CC Set exchange studies are shown. Samples were incubated at 25°C in PBS buffer and measurements were taken 1 hr (A) and 24 hrs (B) post-mixing.

Table S10. Orthogonal CC Set Fluorescence Data

FAM-labelled Peptide	Unlabelled Peptide	1 hr-raw			1 hr-normalised			Avg	Std Dev
FAM-CC-Di	-	2235	2372	2256	-0.087	-0.048	-0.081	-0.072	0.021
FAM-CC-Di	CC-Di	5577	5880	5582	0.884	0.972	0.885	0.914	0.050
FAM-CC-Di	CC-Tri	2470	2488	2603	-0.019	-0.014	0.019	-0.005	0.021
FAM-CC-Di	CC-Tet*	2319	2486	2418	-0.063	-0.015	-0.034	-0.037	0.024
FAM-CC-Di	CC-Pent2-hen3	2467	2533	2419	-0.020	-0.001	-0.034	-0.018	0.017
FAM-CC-Di	CC-Hex2	2354	2520	2471	-0.053	-0.005	-0.019	-0.025	0.025
FAM-CC-Di	CC-Hept-IV-hen2	2421	2598	2523	-0.033	0.018	-0.004	-0.006	0.026
FAM-CC-Tri	-	631	685	671	-0.015	-0.007	-0.009	-0.010	0.004
FAM-CC-Tri	CC-Di	758	728	769	0.002	-0.002	0.004	0.001	0.003
FAM-CC-Tri	CC-Tri	867	928	879	0.017	0.025	0.018	0.020	0.004
FAM-CC-Tri	CC-Tet*	942	948	891	0.027	0.028	0.020	0.025	0.004
FAM-CC-Tri	CC-Pent2-hen3	778	829	804	0.005	0.012	0.008	0.008	0.003
FAM-CC-Tri	CC-Hex2	871	897	861	0.017	0.021	0.016	0.018	0.002
FAM-CC-Tri	CC-Hept-IV-hen2	823	776	783	0.011	0.005	0.006	0.007	0.003
FAM-CC-Tet*	-	860	947	908	-0.026	-0.017	-0.021	-0.021	0.004
FAM-CC-Tet*	CC-Di	843	943	953	-0.027	-0.017	-0.016	-0.020	0.006
FAM-CC-Tet*	CC-Tri	954	1000	960	-0.016	-0.012	-0.016	-0.015	0.002
FAM-CC-Tet*	CC-Tet*	1125	1227	1173	0.001	0.011	0.005	0.006	0.005
FAM-CC-Tet*	CC-Pent2-hen3	1209	1340	1167	0.009	0.022	0.005	0.012	0.009
FAM-CC-Tet*	CC-Hex2	1217	1351	1171	0.010	0.023	0.005	0.013	0.009
FAM-CC-Tet*	CC-Hept-IV-hen2	1025	1043	932	-0.009	-0.008	-0.019	-0.012	0.006
FAM-CC-Pent2-hen3	-	2096	1962	1353	0.056	0.044	-0.011	0.030	0.036
FAM-CC-Pent2-hen3	CC-Di	2028	2120	1356	0.050	0.058	-0.011	0.033	0.038
FAM-CC-Pent2-hen3	CC-Tri	2074	2053	1404	0.054	0.052	-0.006	0.033	0.034
FAM-CC-Pent2-hen3	CC-Tet*	2226	2257	1696	0.068	0.071	0.020	0.053	0.029
FAM-CC-Pent2-hen3	CC-Pent2-hen3	6715	6660	6742	0.474	0.469	0.477	0.474	0.004

FAM-CC-Pent2-hen3	CC-Hex2	3753	6373	3633	0.206	0.443	0.195	0.282	0.140
FAM-CC-Pent2-hen3	CC-Hept-IV-hen2	2430	2282	1581	0.086	0.073	0.010	0.056	0.041
FAM-CC-Hex2	-	937	916	854	-0.064	-0.066	-0.074	-0.068	0.005
FAM-CC-Hex2	CC-Di	904	946	749	-0.068	-0.063	-0.086	-0.072	0.012
FAM-CC-Hex2	CC-Tri	875	935	805	-0.071	-0.064	-0.079	-0.072	0.008
FAM-CC-Hex2	CC-Tet*	989	985	909	-0.058	-0.058	-0.067	-0.061	0.005
FAM-CC-Hex2	CC-Pent2-hen3	1292	1208	1249	-0.022	-0.032	-0.027	-0.027	0.005
FAM-CC-Hex2	CC-Hex2	4124	5615	5855	0.311	0.487	0.515	0.438	0.110
FAM-CC-Hex2	CC-Hept-IV-hen2	5873	6211	5365	0.517	0.557	0.458	0.511	0.050
FAM-CC-Hept-IV-hen2	-	1705	1859	1931	0.041	0.054	0.060	0.052	0.010
FAM-CC-Hept-IV-hen2	CC-Di	2452	1929	1710	0.103	0.060	0.042	0.068	0.031
FAM-CC-Hept-IV-hen2	CC-Tri	2426	1895	1878	0.101	0.057	0.056	0.071	0.026
FAM-CC-Hept-IV-hen2	CC-Tet*	1969	1721	1706	0.063	0.043	0.042	0.049	0.012
FAM-CC-Hept-IV-hen2	CC-Pent2-hen3	1891	2231	1864	0.057	0.085	0.055	0.065	0.017
FAM-CC-Hept-IV-hen2	CC-Hex2	2103	2099	1855	0.074	0.074	0.054	0.067	0.012
FAM-CC-Hept-IV-hen2	CC-Hept-IV-hen2	2201	1963	1739	0.082	0.063	0.044	0.063	0.019

**FAM-labelled Peptide**	**Unlabelled Peptide**	**24 hr-raw**			**24 hr-normalised**			**Avg**	**Std Dev**
FAM-CC-Di	-	2366	2328	2333	-0.049	-0.060	-0.059	-0.056	0.006
FAM-CC-Di	CC-Di	5900	5485	5847	0.978	0.857	0.962	0.932	0.066
FAM-CC-Di	CC-Tri	2445	2300	2675	-0.026	-0.069	0.040	-0.018	0.055
FAM-CC-Di	CC-Tet*	2304	2337	2617	-0.067	-0.058	0.024	-0.034	0.050
FAM-CC-Di	CC-Pent2-hen3	2583	2460	2533	0.014	-0.022	-0.001	-0.003	0.018
FAM-CC-Di	CC-Hex2	2441	2363	2644	-0.028	-0.050	0.031	-0.015	0.042
FAM-CC-Di	CC-Hept-IV-hen2	2571	2412	2732	0.010	-0.036	0.057	0.010	0.046
FAM-CC-Tri	-	646	620	712	-0.013	-0.016	-0.004	-0.011	0.006
FAM-CC-Tri	CC-Di	767	629	794	0.003	-0.015	0.007	-0.001	0.012
FAM-CC-Tri	CC-Tri	1279	1118	1233	0.071	0.050	0.065	0.062	0.011

FAM-CC-Tri	CC-Tet*	1251	1194	1316	0.068	0.060	0.076	0.068	0.008
FAM-CC-Tri	CC-Pent2-hen3	755	727	785	0.002	-0.002	0.006	0.002	0.004
FAM-CC-Tri	CC-Hex2	1232	1144	1329	0.065	0.054	0.078	0.066	0.012
FAM-CC-Tri	CC-Hept-IV-hen2	808	725	765	0.009	-0.002	0.003	0.003	0.006
FAM-CC-Tet*	-	903	836	894	-0.021	-0.028	-0.022	-0.024	0.004
FAM-CC-Tet*	CC-Di	836	888	890	-0.028	-0.023	-0.023	-0.025	0.003
FAM-CC-Tet*	CC-Tri	939	977	950	-0.018	-0.014	-0.017	-0.016	0.002
FAM-CC-Tet*	CC-Tet*	1743	2224	1775	0.062	0.109	0.065	0.079	0.027
FAM-CC-Tet*	CC-Pent2-hen3	2275	2827	2231	0.114	0.169	0.110	0.131	0.033
FAM-CC-Tet*	CC-Hex2	2807	3815	2698	0.167	0.267	0.156	0.197	0.061
FAM-CC-Tet*	CC-Hept-IV-hen2	987	1006	1042	-0.013	-0.011	-0.008	-0.011	0.003
FAM-CC-Pent2-hen3	-	1827	1818	1312	0.032	0.031	-0.015	0.016	0.027
FAM-CC-Pent2-hen3	CC-Di	1857	1915	1325	0.035	0.040	-0.014	0.020	0.029
FAM-CC-Pent2-hen3	CC-Tri	1749	1761	1262	0.025	0.026	-0.019	0.011	0.026
FAM-CC-Pent2-hen3	CC-Tet*	2496	2494	1906	0.092	0.092	0.039	0.075	0.031
FAM-CC-Pent2-hen3	CC-Pent2-hen3	12100	12029	10059	0.962	0.956	0.777	0.898	0.105
FAM-CC-Pent2-hen3	CC-Hex2	9002	8133	8658	0.681	0.603	0.650	0.645	0.040
FAM-CC-Pent2-hen3	CC-Hept-IV-hen2	2624	2577	1610	0.104	0.100	0.012	0.072	0.052
FAM-CC-Hex2	-	460	391	373	-0.008	-0.014	-0.015	-0.013	0.004
FAM-CC-Hex2	CC-Di	416	396	415	-0.012	-0.014	-0.012	-0.012	0.001
FAM-CC-Hex2	CC-Tri	441	372	397	-0.010	-0.016	-0.013	-0.013	0.003
FAM-CC-Hex2	CC-Tet*	2668	3237	2515	0.175	0.223	0.163	0.187	0.032
FAM-CC-Hex2	CC-Pent2-hen3	3710	3699	3584	0.262	0.261	0.251	0.258	0.006
FAM-CC-Hex2	CC-Hex2	11610	11929	11367	0.919	0.945	0.899	0.921	0.023
FAM-CC-Hex2	CC-Hept-IV-hen2	857	1287	850	0.025	0.061	0.024	0.036	0.021
FAM-CC-Hept-IV-hen2	-	999	1042	1033	-0.057	-0.051	-0.053	-0.054	0.003
FAM-CC-Hept-IV-hen2	CC-Di	862	1020	825	-0.073	-0.054	-0.077	-0.068	0.012
FAM-CC-Hept-IV-hen2	CC-Tri	886	909	772	-0.070	-0.067	-0.083	-0.073	0.009
FAM-CC-Hept-IV-hen2	CC-Tet*	869	939	796	-0.072	-0.064	-0.080	-0.072	0.008

FAM-CC-Hept-IV-hen2	CC-Pent2-hen3	1293	1271	1320	-0.022	-0.025	-0.019	-0.022	0.003
FAM-CC-Hept-IV-hen2	CC-Hex2	6400	5410	6664	0.579	0.463	0.611	0.551	0.078
FAM-CC-Hept-IV-hen2	CC-Hept-IV-hen2	6363	6653	3908	0.575	0.609	0.286	0.490	0.178

**FAM-labelled Peptide**	**Unlabelled Peptide**	**annealed-raw**			**annealed-normalised**			**Avg**	**Std Dev**
FAM-CC-Di	-	2170	2669	2769	-0.106	0.039	0.068	0.000	0.093
FAM-CC-Di	CC-Di	5943	6068	5920	0.990	1.026	0.983	1.000	0.023
FAM-CC-Di	CC-Tri	2837	2642	2676	0.087	0.031	0.041	0.053	0.030
FAM-CC-Di	CC-Tet*	2679	2730	2779	0.042	0.056	0.071	0.056	0.015
FAM-CC-Di	CC-Pent2-hen3	2835	2713	2711	0.087	0.051	0.051	0.063	0.021
FAM-CC-Di	CC-Hex2	2720	2703	2593	0.053	0.049	0.017	0.040	0.020
FAM-CC-Di	CC-Hept-IV-hen2	2511	2510	2448	-0.007	-0.008	-0.026	-0.013	0.010
FAM-CC-Tri	-	751	717	754	0.001	-0.003	0.002	0.000	0.003
FAM-CC-Tri	CC-Di	1046	888	975	0.041	0.020	0.031	0.030	0.010
FAM-CC-Tri	CC-Tri	8243	8496	8093	0.995	1.029	0.976	1.000	0.027
FAM-CC-Tri	CC-Tet*	2890	2818	2853	0.285	0.276	0.280	0.280	0.005
FAM-CC-Tri	CC-Pent2-hen3	828	805	800	0.012	0.009	0.008	0.009	0.002
FAM-CC-Tri	CC-Hex2	781	794	822	0.005	0.007	0.011	0.008	0.003
FAM-CC-Tri	CC-Hept-IV-hen2	804	738	746	0.008	0.000	0.001	0.003	0.005
FAM-CC-Tet*	-	1104	1118	1136	-0.002	0.000	0.002	0.000	0.002
FAM-CC-Tet*	CC-Di	1145	1224	1180	0.003	0.010	0.006	0.006	0.004
FAM-CC-Tet*	CC-Tri	1269	1319	1299	0.015	0.020	0.018	0.017	0.002
FAM-CC-Tet*	CC-Tet*	11307	11171	11160	1.009	0.996	0.995	1.000	0.008
FAM-CC-Tet*	CC-Pent2-hen3	2657	2853	2843	0.152	0.172	0.171	0.165	0.011
FAM-CC-Tet*	CC-Hex2	2129	2748	2815	0.100	0.161	0.168	0.143	0.037
FAM-CC-Tet*	CC-Hept-IV-hen2	1086	1121	969	-0.003	0.000	-0.015	-0.006	0.008
FAM-CC-Pent2-hen3	-	1579	1748	1097	0.009	0.025	-0.034	0.000	0.031
FAM-CC-Pent2-hen3	CC-Di	1658	1821	1084	0.017	0.031	-0.035	0.004	0.035

FAM-CC-Pent2-hen3	CC-Tri	1664	1748	1071	0.017	0.025	-0.037	0.002	0.033
FAM-CC-Pent2-hen3	CC-Tet*	3167	3332	2078	0.153	0.168	0.055	0.125	0.062
FAM-CC-Pent2-hen3	CC-Pent2-hen3	13302	13881	10377	1.071	1.123	0.806	1.000	0.170
FAM-CC-Pent2-hen3	CC-Hex2	4814	3926	3748	0.302	0.222	0.206	0.243	0.052
FAM-CC-Pent2-hen3	CC-Hept-IV-hen2	2886	2623	2349	0.128	0.104	0.079	0.104	0.024
FAM-CC-Hex2	-	632	499	546	0.006	-0.005	-0.001	0.000	0.006
FAM-CC-Hex2	CC-Di	567	508	572	0.001	-0.004	0.001	-0.001	0.003
FAM-CC-Hex2	CC-Tri	615	519	592	0.005	-0.003	0.003	0.001	0.004
FAM-CC-Hex2	CC-Tet*	4064	3828	4041	0.291	0.272	0.289	0.284	0.011
FAM-CC-Hex2	CC-Pent2-hen3	3295	3210	3175	0.227	0.220	0.217	0.222	0.005
FAM-CC-Hex2	CC-Hex2	13004	11976	12783	1.035	0.949	1.016	1.000	0.045
FAM-CC-Hex2	CC-Hept-IV-hen2	784	1213	859	0.019	0.054	0.025	0.033	0.019
FAM-CC-Hept-IV-hen2	-	1154	1172	2112	-0.038	-0.036	0.074	0.000	0.065
FAM-CC-Hept-IV-hen2	CC-Di	1056	1201	1053	-0.050	-0.033	-0.050	-0.044	0.010
FAM-CC-Hept-IV-hen2	CC-Tri	1103	907	937	-0.044	-0.067	-0.064	-0.059	0.012
FAM-CC-Hept-IV-hen2	CC-Tet*	1101	1086	991	-0.045	-0.046	-0.058	-0.049	0.007
FAM-CC-Hept-IV-hen2	CC-Pent2-hen3	1575	1419	1447	0.011	-0.007	-0.004	0.000	0.010
FAM-CC-Hept-IV-hen2	CC-Hex2	3246	2687	3311	0.208	0.142	0.216	0.189	0.040
FAM-CC-Hept-IV-hen2	CC-Hept-IV-hen2	9536	9460	10919	0.949	0.940	1.112	1.000	0.097

References

1. Fletcher, J. M.; Boyle, A. L.; Bruning, M.; Bartlett, G. J.; Vincent, T. L.; Zaccai, N. R.; Armstrong, C. T.; Bromley, E. H. C.; Booth, P. J.; Brady, R. L.; Thomson, A. R.; Woolfson, D. N A Basis Set of Coiled-Coil Peptide Oligomers for Rational Protein Design and Synthetic Biology. Acs Synth Biol 2012, 1 (6), 240-250. DOI: 10.1021/sb300028q.
2. Edgell, C. L.; Savery, N. J.; Woolfson, D. N. Robust de novo Designed Homotetrameric Coiled Coils. Biochemistry-Us 2020, 59 (10), 1087-1092. DOI: 10.1021/acs.biochem.0c00082.
3. Dawson, W. M.; Martin, F. J. O.; Rhys, G. G.; Shelley, K. L.; Brady, R. L.; Woolfson, D. N. Coiled coils 9-to-5: rational design of α-helical barrels with tunable oligomeric states. Chem. Sci. 2021, 12 (20), 6923-6928. DOI: 10.1039/d1sc00460c.
4. Martin, F. J. O. Exploring the Dynamic and Conformational Landscape of α-Helical Peptide Assemblies. Ph. D. Dissertation, University of Bristol, Bristol, UK, 2022.
5. Schuck, P. Size-distribution analysis of macromolecules by sedimentation velocity ultracentrifugation and lamm equation modeling. Biophys J 2000, 78 (3), 1606-1619. DOI: 10.1016/S0006-3495(00)76713-0.
6. Schuck, P. On the analysis of protein self-association by sedimentation velocity analytical ultracentrifugation. Anal Biochem 2003, 320 (1), 104-124. DOI: 10.1016/s0003-2697(03)00289-6.
7. Winter, G. xia2: an expert system for macromolecular crystallography data reduction. Journal of Applied Crystallography 2010, 43, 186-190. DOI: 10.1107/S0021889809045701.
8. Winter, G.; Waterman, D. G.; Parkhurst, J. M.; Brewster, A. S.; Gildea, R. J.; Gerstel, M.; Fuentes-Montero, L.; Vollmar, M.; Michels-Clark, T.; Young, I. D.; et al. DIALS: implementation and evaluation of a new integration package. Acta Crystallogr D Struct Biol 2018, 74 (Pt 2), 85-97. DOI: 10.1107/S2059798317017235.
9. Powell, H. R. The Rossmann Fourier autoindexing algorithm in MOSFLM. Acta Crystallogr D Biol Crystallogr 1999, 55 (Pt 10), 1690-1695. DOI: 10.1107/s0907444999009506.
10. Evans, P. R.; Murshudov, G. N. How good are my data and what is the resolution? Acta Crystallogr D 2013, 69, 1204-1214. DOI: 10.1107/S0907444913000061.
11. Winn, M. D.; Ballard, C. C.; Cowtan, K. D.; Dodson, E. J.; Emsley, P.; Evans, P. R.; Keegan, R. M.; Krissinel, E. B.; Leslie, A. G. W.; McCoy, A.; et al. Overview of the ccp4 suite and current developments. Acta Crystallographica Section D-Structural Biology 2011, 67, 235-242. DOI: 10.1107/S0907444910045749.
12. Kabsch, W. Xds. Acta Crystallogr D 2010, 66, 125-132. DOI: 10.1107/S0907444909047337.
13. Vonrhein, C.; Flensburg, C.; Keller, P.; Sharff, A.; Smart, O.; Paciorek, W.; Womack, T.; Bricogne, G. Data processing and analysis with the autoPROC toolbox. Acta Crystallogr D Biol Crystallogr 2011, 67 (Pt 4), 293-302. DOI: 10.1107/S0907444911007773.
14. Rodriguez, D. D.; Grosse, C.; Himmel, S.; Gonzalez, C.; de Ilarduya, I. M.; Becker, S.; Sheldrick, G. M.; Uson, I. Crystallographic ab initio protein structure solution below atomic resolution. Nat Methods 2009, 6 (9), 651-653. DOI: 10.1038/nmeth.1365.
15. Caballero, I.; Sammito, M.; Millan, C.; Lebedev, A.; Soler, N.; Uson, I. ARCIMBOLDO on coiled coils. Acta Crystallogr D Struct Biol 2018, 74 (Pt 3), 194-204. DOI: 10.1107/S2059798317017582.
16. McCoy, A. J.; Grosse-Kunstleve, R. W.; Adams, P. D.; Winn, M. D.; Storoni, L. C.; Read, R. J. Phaser crystallographic software. J Appl Crystallogr 2007, 40 (Pt 4), 658-674. DOI: 10.1107/S0021889807021206.
17. Mirdita, M.; Schutze, K.; Moriwaki, Y.; Heo, L.; Ovchinnikov, S.; Steinegger, M. ColabFold: making protein folding accessible to all. Nat Methods 2022, 19 (6), 679-682. DOI: 10.1038/s41592-022-01488-1.
18. Cowtan, K. The Buccaneer software for automated model building. 1. Tracing protein chains. Acta Crystallogr D Biol Crystallogr 2006, 62 (Pt 9), 1002-1011. DOI: 10.1107/S0907444906022116.

19. Casanal, A.; Lohkamp, B.; Emsley, P. Current developments in Coot for macromolecular model building of Electron Cryo-microscopy and Crystallographic Data. Protein Sci 2020, 29 (4), 1069-1078. DOI: 10.1002/pro.3791.
20. Murshudov, G. N.; Skubak, P.; Lebedev, A. A.; Pannu, N. S.; Steiner, R. A.; Nicholls, R. A.; Winn, M. D.; Long, F.; Vagin, A. A. REFMAC5 for the refinement of macromolecular crystal structures. Acta Crystallogr D Biol Crystallogr 2011, 67 (Pt 4), 355-367. DOI: 10.1107/S0907444911001314.
21. Plecs, J. J.; Harbury, P. B.; Kim, P. S.; Alber, T. Structural test of the parameterized-backbone method for protein design. J. Mol. Biol. 2004, 342 (1), 289-297. DOI: 10.1016/j.jmb.2004.06.051.
